# Divergent Evolutionary and Expression Patterns between Lineage Specific New Duplicate Genes and Their Parental Paralogs in *Arabidopsis thaliana*

**DOI:** 10.1371/journal.pone.0072362

**Published:** 2013-08-29

**Authors:** Jun Wang, Nicholas C. Marowsky, Chuanzhu Fan

**Affiliations:** Department of Biological Sciences, Wayne State University, Detroit, Michigan, United States of America; North Carolina State University, United States of America

## Abstract

Gene duplication is an important mechanism for the origination of functional novelties in organisms. We performed a comparative genome analysis to systematically estimate recent lineage specific gene duplication events in *Arabidopsis thaliana* and further investigate whether and how these new duplicate genes (NDGs) play a functional role in the evolution and adaption of *A. thaliana*. We accomplished this using syntenic relationship among four closely related species, *A. thaliana*, *A. lyrata*, *Capsella rubella* and *Brassica rapa*. We identified 100 NDGs, showing clear origination patterns, whose parental genes are located in syntenic regions and/or have clear orthologs in at least one of three outgroup species. All 100 NDGs were transcribed and under functional constraints, while 24% of the NDGs have differential expression patterns compared to their parental genes. We explored the underlying evolutionary forces of these paralogous pairs through conducting neutrality tests with sequence divergence and polymorphism data. Evolution of about 15% of NDGs appeared to be driven by natural selection. Moreover, we found that 3 NDGs not only altered their expression patterns when compared with parental genes, but also evolved under positive selection. We investigated the underlying mechanisms driving the differential expression of NDGs and their parents, and found a number of NDGs had different *cis*-elements and methylation patterns from their parental genes. Overall, we demonstrated that NDGs acquired divergent *cis*-elements and methylation patterns and may experience sub-functionalization or neo-functionalization influencing the evolution and adaption of *A. thaliana*.

## Introduction

Genes that have more recent origins, namely new genes, are merited with enormous evolutionary significance such as the origin of biological diversity and a source of novel functions. Lineage specific new genes are a class of genes defined as the coding genes that do not have orthologs in other species. It could be inferred that lineage specific new genes are just the results of missing annotation of genes between species. However, studies have shown that lineage specific new genes indeed exist, have originated in multiple organisms and play important roles in the evolution of genomes and organisms [1]–[3]. Many recent studies have also shown that new genes contribute to evolutionary changes and phenotypic adaptation in recently diverged lineages [4]–[14]. Using comparative genomics approaches between closely related species, genome wide identification of lineage specific new genes has been conducted in various animal and plant species [15]–[18].

Genome duplication, exon-shuffling, retroposition, horizontal gene transfer, *de novo* formation, and gene origination mediated by mobile elements have been ascribed as probable molecular mechanisms generating new genes. Among them, whole-genome duplication has played an important role in gene duplication and origination in plants [19]–[23]. However, DNA-based and RNA-based small-scale gene duplications such as tandem and dispersed duplication have also been demonstrated as common mechanisms for recent gene origination in plants [23]–[27]. Gene duplication can give rise to the extra copies of a sequence which can then evolve novel functions [28]–[37].

Both experimental (e.g. array-based comparative genomic hybridization CGH) and computational (e.g. blast-based comparative genomic sequence comparison) approaches have been applied to investigate gene duplication in *A. thaliana* [12], [24], [27], [38]–[43]. For the experimental approach, due to the limitation of available microarrays for non-model species and sequence divergence between species, application of array-based CGH is technically challenged to obtain reliable new gene candidates and often encountered high false positive rates [24]. Previous computational analyses using genomic sequences from multiple species compared all the annotated protein-coding genes in the *A. thaliana* genome to “as many existing sequences as possible” [38], [39]. Furthermore, Donoghue et al (2011) used the “position-specific methods” to detect weak homology between genes in different species [38]. There are two caveats for previous computational analysis. First, although they performed the comparison between *A. thaliana* and “as many existing genome sequences as possible”, due to the limitation of available genome sequences from closely related species at that moment, some false positive genes will be mistakenly annotated. Second, to reveal the weak homologous relationship between genomes, it is necessary to construct whole genome syntenic regions, which has not been employed in these previous analyses. Here, we aimed to investigate the scope, content and evolution of the new genes generated by gene duplication in *A. thaliana* lineage using comparative genomics among multiple closely related species. In addition to genome sequences from *A. lyrata* and *B. rapa*, we added the recently released *C. rubella* genome sequences to the genome comparison [44]. We further constructed whole genome syntenic regions between *A. thaliana* and *A. lyrata*/*C. rubella*/*B. rapa*, respectively. We tested the functionality, analyzed the expression pattern, and explored the *cis*-regulatory motifs and methylation patterns of these NDGs. Furthermore, by taking advantage of newly released SNP data from 80 wild *A. thaliana* accessions, we investigated and compared the underlying evolutionary forces of the NDGs and their parental genes with population genetic analyses, which has not be done before.

**Figure 1:**
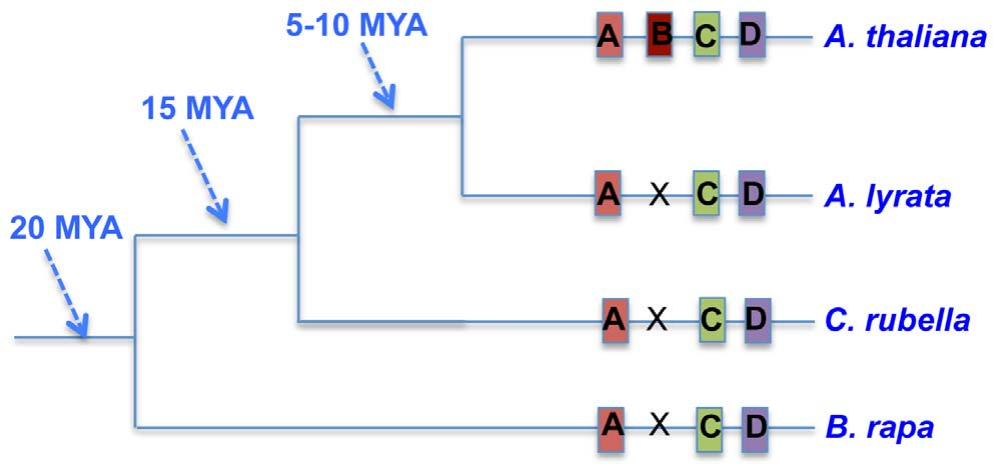
The phylogeny and divergent time among four species. Lineage specific new gene ‘B’ is identified using comparative genomics and syntenic relationship among four genomes.

*Arabidopsis thaliana* is a self-compatible annual flower plant. It is one of the most important model organisms due to its several research advantages including small size, short generation time, large number of seeds and relatively small genome. The 121 Mb sequenced genome size of *A. thaliana* is one of the smallest among angiosperm genomes. 27,416 protein-coding genes were annotated in *A. thaliana* genome [45]. For the other three closely related species used in our study, *B. rapa* has the largest sequenced genome about 290 Mb and contains 10 chromosomes [46], *A. lyrata* has the middle size sequenced genome about 210 Mb and contains 8 chromosomes [47] and *C. rubella* has relatively smaller sequenced genome size about 136 Mb and contains 8 chromosomes. Previous phylogenetic analysis estimated that *B. rapa* separated from *A. thaliana* about 13–17 million years ago (MYA) [48], [49]; *C. rubella* diverged from *A. thaliana* about 10–14 MYA [50]; and *A. lyrata* split from *A. thaliana* about 5–10 MYA [51]–[53] (Figure 1).

## Materials and Methods

### Plant Species Chosen and Genome Sequence Data Sets Selected

We selected four closely related species, *A. thaliana*, *A. lyrata*, *C. rubella*, and *B. rapa*, for comparative genomics analysis to identify *A. thaliana* specific new genes that originated through gene duplication. Given the short divergence time between *A. thaliana* and *A. lyrata*/*C. rubella*/*B. rapa*, we chose genome data of these three species to polarize our analysis and detect the well-conserved syntenies between species. We acquired the complete genome framework datasets including assembly and annotation from Phytozome v8.0 (http://www.phytozome.net/) with *A. thaliana* 167 (TAIR release 10 acquired from TAIR), *A. lyrata* 107 (JGI release v1.0), *C. rubella* 183 (JGI annotation v1.0 on assembly v1), *B. rapa* 197 (Annotation v1.2 on assembly v1.1 from brassicadb.org) genome data.

### Identification of *A. thaliana* Lineage Specific New Genes that Originated through Gene Duplication

To identify *A. thaliana* specific new genes, we selected new genes based on two criteria: first, the gene was not located in any of the syntenic regions between *A. thaliana* and the rest of three species *A. lyrata*, *C. rubella*, *B. rapa*; second, the gene did not have any reciprocal ortholog in *A. lyrata*, *C. rubella* and *B. rapa*.

Using the pipelines developed by UCSC genome browser [54], we constructed the reciprocal syntenic relationship between *A. thaliana* and *A. lyrata*/*C. rubella*/*B. rapa*. We followed five steps to construct the synteny: (1) we used Repeatmasker to mask the repeat regions of *A. thaliana*, *A. lyrata*, *C. rubella* and *B. rapa* genomes [55]. (2) We aligned refSeq of the four genomes with each other using blastz [56]. We then transformed the ‘lav’ output format of blastz to ‘axt’ format using lavToAxt. (3) We chained the ‘axt’ files using axtChain and generated chain format outputs. We further sorted and merged our chain file with chainMergeSort. (4) We netted our chain files generated from previous steps using chainPreNet, chainNet and netSyntenic to pick up the best and longest chain. We also used faSize to calculate the size of chromosomes or scaffolds involved the alignment. (5) We used faToTwoBit to switch the ‘fasta’ format of the chromosome or scaffold sequences into ‘2bit’ format. We transformed the ‘net’ format back to ‘axt’ format using netToAxt. We constructed the genome wide syntenic regions between two genomes by reading the headline of ‘axt’ format output. Overall, we used both genomes as query/hit, and hit/query, respectively, to construct reciprocal syntenic relationships between the *A. thaliana* genome and the other three species.

To identify orthologs, we used BLASTP to search for the reciprocal best hits between *A. thaliana* and *A. lyrata*/*C. rubella*/*B. rapa* [57]. We defined the genes with reciprocal best hits and the alignment e-value lower than 0.001 [38] in these species as orthologous genes. After the construction of synteny and identification of orthologs between *A. thaliana* and the other three species, we were able to identify the *A. thaliana* lineage specific genes that were evolved recently after *A. thaliana* diverged.

We analyzed the gene structure and genome context, and further performed the paralog search to identify the origination of *A. thaliana* new genes that were generated through gene duplication. To determine if a gene was generated through gene duplication, we performed BLAT for the peptide sequences of *A. thaliana* lineage specific new genes against all the peptide sequences of *A. thaliana* genome [58]. We chose the gene pairs satisfying ≥50% alignment identity and ≥70% alignment coverage at the amino acid level as the candidate paralog pairs generated through gene duplication. We then used the peptides of the two genes of paralog pairs in *A. thaliana* to blat against all the peptides in *A. lyrata*, *C. rubella*, and *B. rapa*. We also used the CDSs of the two genes of paralog pairs in *A. thaliana* to blat against the whole genomes of *A. lyrata*, *C. rubella*, and *B. rapa.* We determined the *A. thaliana* NDGs from the paralogous pairs with one of the following three situations: (1) no hits in other three species; (2) two paralogous genes sharing one best hit in other three species (namely, one ortholog in other species, and two duplicate copies in *A. thaliana*), (3) the new gene having no hit but the other gene having one hit.

To identify if a gene was formed with a chimeric gene structure by recruiting gene fragment(s) from other DNA sequence sources, we compared the gene structure and DNA sequences of paralogs to determine if NDGs were recruiting DNA sequences from target sites. We also blat the peptide sequences of *A. thaliana* lineage specific genes against all the peptide sequences of *A. thaliana* genome, and chose the gene pairs satisfying that the different regions of one lineage-specific gene aligned more than one gene. We compared the location of duplicate genes. We defined paralogs as generated by tandem duplication mechanism if both copies are adjacent to each other. We defined segmental gene duplication if two paralogous pairs were distanced within 10 genes and two copies of each pair in the segments are syntenic, respectively. This does not limit the length of one segment to contain only 10 genes (see Figure S1). To polarize the parent/daughter relationship of tandem duplicates, we used syntenic map and/or phylogeny analysis of paralogs and their orthologs in outgroup species. The gene copy with lower than 30% (in most case, it is 0) of the length in the syntenic regions was defined as NDG while the copy with higher than 30% (in most case, it is 100%) of the length in syntenic regions was defined as parental gene (see Figure S2). When both copies are located or not located in syntenic regions but have one orthologous gene in each of the outgroup species, we drew the gene tree (neighbor-joining tree with 1000 bootstraps) with two paralogous genes and their orthologs in outgroup species to determine the parental/daughter relationship. We defined the copy clustering with orthologs as the parental gene (see Figure S3).

### The Fixation of NDGs and Parental Genes in 18 Additional *A. thaliana* Accessions

Genome data of 18 accessions of *A. thaliana*, Bur-0, Can-0, Ct-1, Edi-0, Hi-0, Kn-0, Ler-0, Mt-0, No-0, Po-0, Oy-0, Rsch-4, Sf-2, Tsu-0, Wil-2, Ws-0, Wu-0 and Zu-0 were downloaded from http://mus.well.ox.ac.uk/19genomes/. We blat the peptides of 100 genes to all the peptides of 18 genomes. 63 gene pairs had both the parental and NDGs hits to the corresponding annotated genes in the 18 genomes. We further used the CDS of the remaining 37 pairs to blat the whole genome sequences of the 18 genomes. We found that the 37 pairs had either parental gene or NDG hits to the unannotated genome sequences or did not have hits in the 18 genomes. We further identified 23 of the 37 pairs that had NDGs and parental genes hit to different genomic locations, indicating both parental genes and offspring genes had homolog sequences in the 18 genomes. We used syntenic information or reciprocal best hits information to annotate the 14 of the 37 pairs whose parental genes or NDGs had the same genomic hits or lack genomic hits.

### Functionality Analysis Using Sequence Divergent Tests

To examine the functional constraints on these NDGs, we computed Ka/Ks ratios (ω) of the identified paralog pairs using PAML [59] and estimated whether ω was significantly smaller than 0.5 and 1 [60]. A Ka/Ks ratio higher than unity (ω = 1) indicates positive selection, and lower than unity indicates the functional constraint. Conservatively, we considered genes with Ka/Ks ratio significantly smaller than 0.5 as functional constraints on both paralogous genes [61]. Using MAFFT [62], we aligned the CDSs of each paralog pair according to their protein alignment. Then we performed Codeml of PAML with two models: model 1 fixing ω at 0.5 or 1, and model 2 estimating ω freely. We then conducted Likelihood Ratio Test (LRT), which tested whether the likelihood of model 2 was significantly smaller than that of model 1 with ω = 0.5 or with ω = 1 by comparing two times the log likelihood difference as 2L = 2(L_0.5_– L_0_) or 2L = 2(L_1_– L_0_). P values were calculated using a Chi-square distribution with one degree of freedom [63].

To compute the branch specific ω of these NDGs, we first collected available CDSs of the outgroup species, and aligned the duplicate genes and their outgroup orthologous sequences with MACSE [64]. Then we used Codeml of PAML with parameter “model = 2” to estimate the branch specific ω of NDG and background ω. To conduct the LRT of branch specific ω model, we compared the model with background and foreground ω varying freely to the model with background ω varying freely and foreground ω fixed to 1. Significance levels of likelihoods, as *p* values, from the two models, were calculated using Chi-square distribution with one degree of freedom.

### Population Genetics Analysis and MacDonald & Kreitman Test

We obtained the SNP data generated from a complete re-sequencing of 80 strains of *A. thaliana* using next-generation sequencing technology [65]. We then collected SNPs in the gene regions for both the NDGs and their parental genes. We used Perl scripts to compute the population parameters (e.g. π and θ) and test the frequency spectra of the polymorphism in both NDG and its parental gene with Tajima’s D [66] and Fu & Li’s D and F [67] methods. We assessed the significance (*p* value) of all the three tests by comparing the neutrality test values (e.g. Tajima’s D, Fu & Li’s D and F) of each NDG or its paralog to the empirical distribution of neutrality test values from large data set [68], [69]. The empirical distribution of these neutrality test statistic was generated from 1000 randomly picked loci distributed across the genome. Since linkage disequilibrium in *A. thaliana* decays on average within 25–50 Kb [70], we removed loci with <25 Kb distance between them to exclude loci bearing dependent evolutionary history [68]. After this selection process, a total of ∼800 loci were sampled to estimate empirical distribution. We then compared Tajima’s D, Fu and Li’s D and F for each NDG or its parental gene to the empirical distribution from this large data set. If the Tajimas’s D, Fu and Li’s D and F were negative, we computed the ‘p’ value as Proportion_empirical_(X_emp_≤ X_obs_); if those values were positive, we computed the ‘p’ value as Proportion_empirical_(X_emp_≥ X_obs_), where X_emp_ and X_obs_ are the empirical and observed values, respectively (Figure S4). Statistical significance was obtained from the statistic for each NDGs/parental gene using a 5% type I error for one tail (Figure S4). We used multiple testing correction procedure to adjust statistical confidence based on all NDGs and their parental genes tested. The basis of multiple-testing correction uses false discovery rate (FDR) estimation. Therefore, for each neutrality test, we pooled the ‘p’ values of all NDGs and parental genes together, and computed the corresponding FDR ‘q’ values for each gene. We took FDR ‘q’ value <0.05 and the neutrality test value (e.g. values of Tajima’s D or Fu and Li’s D) <0 as criteria to define if a gene is under natural selection. Lastly, using intraspecific sequence polymorphism and paralogous sequence divergence data, we then integrated DNA sequence polymorphism and divergence data to rigorous McDonald-Kreitman (MK) tests to infer if NDGs were driven by positive selection [71]. The tests were performed for both NDGs and their parental genes to detect if a differential evolution pattern existed between NDGs and parental genes. Comparison of fixed DNA sequence divergence of a NDG and its paralog and polymorphisms of a NDG was used to conduct MK tests on NDGs. Similarly, comparison of fixed DNA sequence divergence of the parental gene and its paralog along with polymorphism of the parental gene was used to conduct MK test on the parental genes. Fisher’s exact test was applied to test the significance level of the null hypothesis of neutrality in MK test.

### Expression Analysis of NDGs and their Parental Genes

We collected the expression data from several sources. First, we searched the NCBI Unigene database (http://www.ncbi.nlm.nih.gov/unigene) to detect if a NDG generated through duplication had been annotated as a Unigene with functions. We downloaded the UniGene EST expression profile with the four column information available: tissue pool name, transcript/EST number per million (TPM), expression enrichment based on TPM and EST number for this gene/EST number for the total pool (see Figure S5). We constructed the following contingency table: EST number in certain tissue for one gene of interest, total EST number for this gene minus EST number in certain tissue for one gene of interest, EST number in certain tissue for all genes, and total EST number for all genes minus EST number in certain tissue for all genes, to test the significance of EST enrichment in certain tissue for one gene. Additionally, we performed an EST-based expression search analysis. We downloaded the NCBI EST library collection of *A. thaliana* from http://www.ncbi.nlm.nih.gov/UniGene/lbrowse2.cgi?TAXID=3702&CUTOFF=0. The collection contains 406,024 ESTs from 111 EST libraries in 12 tissues including aerial organs, buds, cell culture, flower, inflorescence, leaf, root, seed, silique, stem, vegetative tissues, and whole plant. We used BLAT to identify the corresponding genes of the ESTs. The criteria to define the corresponding gene of an EST were as follows: 1) the CDS of this gene was the first best hit of the EST; 2) the alignment of the EST and this best hit gene satisfied ≥95% identity, ≤1e-20 E value, ≥100 blast score; and 3) the blat score of this first best gene hit was at least 5 points [72] higher than that of the second gene hit of the EST. Thus, the corresponding relation between ESTs and 18,550 (67.66% of 27,416 total *A. thaliana* annotated genes) current annotated genes were constructed.

Second, we downloaded the mRNA MPSS data for 17 libraries and Small RNA data for 40 libraries from http://mpss.udel.edu/at/mpss_index.php. Massively Parallel Signature Sequencing (MPSS) expression data were reported in the sum for the abundance of unique signatures in TPM (transcripts per million). Small RNA expression data were reported in the sum for the abundance of all the signatures in TPQ (transcripts per quarter million). Sequence match for small RNA is not required to be a unique signature, because small RNAs can be biologically active in more than one sequence that they match.

Third, we downloaded the processed expression data generated by the Affymetrix GeneChip Tiling 1.0R array from http://genomebiology.com/2008/9/7/R112/additional/. The tiling array contains one 25-base probe in each non-repetitive 35 bp window of the reference genome. RNA samples were collected from 11 different tissues at different stages of *A. thaliana* development. The probes that had duplicate copies and probes that had multiple hits in the genome were removed, and only the probe mapped to constitutive exons of the genes were kept. Robust multi-array average (RMA) method was applied to hybridization data for background correction, quantile normalization and expression estimation. We further defined the tissue specific genes based on the Z score of the gene expression in each of the 11 tissues. When the Z-score of one gene in a certain tissue was larger than 2.5, we defined this gene as a tissue specific gene [73].

Fourth, we added RNA-seq data from 3 tissues of *A. thaliana* from http://www.ncbi.nlm.nih.gov/geo/query/acc.cgi?acc=GSE30795. Because the processed data by Gan et al. (2011) [74] used earlier version refseq, we re-mapped RNA-seq reads to the current version *A. thaliana* refseq (TAIR 10 genome release). We used (1) Bowtie v0.12.8 [75] to map the reads to the genome; (2) picard-tools-1.79 (MarkDuplicates) to remove the duplicates that were generated by PCR, (3) Cufflinks v2.0.2 [76] to estimate gene-level relative abundance in Fragments Per Kilobase of exon model per Million mapped fragments (FPKM) format.

### Methylation Data Analysis

We downloaded the single-base resolution methylation data in *Arabidopsis* published by Lister et al. 2008 [77] through the NCBI short Read Archive accession numbers SRA000284. We re-analyzed the sequencing data using current *A. thaliana* reference genome (TAIR 10 genome release) with Bismark v0.7.7 [78]. The intermediate steps included (1) running quality control, (2) mapping the reads, (3) removing the duplication generated by PCR, (4) generating cytosine methylation reports. Because the chloroplast genome has no methylation activity, any methylation reads detected in chloroplast genome should be accounted for the error. Thus, the error rate (2.21%) that estimated from the chloroplast genome was used as the control. We conducted binomial test for each cytosine base based on methylation reads, non-methylation reads and error rate to test whether a cytosine is methylated. We analyzed the methylation conservation levels between NDGs and parental genes in genic regions and in gene regulatory regions. For genic region, we checked the methylation conservation pattern between NDGs and parental genes in the gene body for cytosine in all the three contexts, namely “CG”, “CHH”, “CHG” (H = A, C, or T). We used the methylation conservation degree of all duplicated genes as the frequency of binomial test. Based on the number of covered cytosines and the number of cytosines with conserved methylation pattern (including conserved methylation and conserved un-methylation), we conducted a binominal test to determine whether the degree of conservation between NDG and parental genes was higher than the degree of conservation for all the duplicated genes in gene body. For gene regulatory regions, we examined and compared the methylation level for NDGs and parental genes in promoter regions (200 bp upstream of the transcriptional start sites) and transcriptional termination regions (200 bp downstream of the transcriptional termination site) [79]. We used the methylation level of the promoter regions of all the genes as the frequency of a binomial test. Based on the number of covered cytosines and the number of methylated cytosines, we conducted a binominal test to estimate whether the methylation levels for NDGs and parental genes in promoter regions were higher than those for all the genes. The same binominal test for transcriptional termination regions was also conducted to determine whether the methylation level in transcriptional termination regions for NDGs and parental genes were higher than those for all the genes. All the intermediate steps were conducted by Perl scripts.

## Results

### Identification of *A. thaliana* Lineage Specific New Genes Through Gene Duplication

We identified 137 lineage specific duplicate genes generated from gene duplication, which satisfy two criteria (1) non-reciprocal orthologs based on Blastp search and (2) in the disruption of syntenic regions based on pipelines developed by UCSC genome browser between *A. thaliana* and the other three species, e.g. *A. lyrata*, *C. rubella* and *B. rapa*. Among the 137 paralogs, 23, 48, and 66 genes derived from tandem duplication, segmental duplication, and dispersed duplication, respectively. To examine the parental/NDG relationship between duplicate genes, we screened the 137 duplicate genes for those whose parental genes happened to be *A. thaliana* lineage-specific genes or had a shared ortholog among the other three species. We found that 37 of 137 paralogs were duplicated from *A. thaliana* lineage specific genes, and the remaining 100 paralogs were recently originated from duplication of non-lineage specific parental genes (Table S1). Therefore, we can define the parental/NDG relationship of the 100 paralogs. Among the 137 paralogs, 17 genes were generated through chimera fusion from one duplicate gene and the flanking region of the target site. Additionally, seven genes were originated through chimeric fusion from at least two duplicate genes, and 4 genes were generated through chimeric fusion of a duplicate gene and a transposable element (TE) (Table S2). By comparing the gene structures between NDGs and their parental genes, all NDGs were generated by DNA-based gene duplication.

We further checked whether the 100 NDGs are fixed in *A. thaliana* species by examining the presence of these NDGs in 18 additional *A. thaliana* accessions whose genomes were recently released [74]. We found a majority of NDGs and their parental genes were fixed except for ten NDGs and five parental genes that lack genomic hits in some of the 18 genomes, indicating they are still fluctuating in the *A. thaliana* species.

### Functionality Analysis of *A. thaliana* Lineage Specific NDGs Using Ka/Ks Test

The ratio of nonsynonymous substitutions per nonsynonymous site (Ka) to the synonymous substitutions per synonymous sites (Ks), ω = Ka/Ks, can be used as a test of natural selection. Positive selection is inferred if ω>1, purifying selection if ω<1, and neutral evolution if ω = 1. We computed the Ka/Ks ratio between the NDGs and their parental genes to determine whether they were under functional constraints. Because all the NDGs were duplicated and originated less than 10 MYA, we observed very low synonymous and non-synonymous substitution rates. The average Ks and Ka values were 0.0860 and 0.0290, respectively. 44 out of 137 paralogs did not have synonymous and non-synonymous substitutions. For the remaining 93 paralogs, 18 had Ka/Ks values greater than 1, and 75 had Ka/Ks values less than one (Table S1 and Table 1). LRT of Ka/Ks ratio further confirmed that 31 of 93 paralog pairs were significantly less than 0.5, and 50 of 93 paralog pairs are significantly less than 1 (Table 1), suggesting a majority of paralog pairs ((44+50)/137 = ∼70%) are under strong functional constraints.

**Table 1:** The proportions of NDGs and parental genes with different Ka/Ks.

	Ka and Ks = 0	Ka/Ks >1	Ka/Ks <1
number of paralogs	44	18	75(50^a^)
percentage	32.12%	13.14%	54.74%(36.50%^a^)

a Ka/Ks significantly less than 1 by LRT test.

Further, we wanted to test whether a paralog pair under strong functional constraints with low ω is due to the parental copy remaining under purifying selection and the new copy evolving neutrally as a pseudogene. To test this we estimated ω for the foreground branch leading to the *A. thaliana* lineage specific new gene and for background branches leading to the parental genes and their orthologous genes in outgroup species (*A. lyrata, C. rubella* and *B. rapa*). We first collected the available outgroup orthologous CDS sequences for 92 parental genes from *A. lyrata, C. rubella* and *B. rapa;* since NDGs are lineage specific and do not have ortholog, and some parental genes only have orthologous sequences but no orthologous CDSs. We then calculated *A. thaliana* branch specific ω for these 92 NDGs (Table S3). 52 of the 92 NDGs have branch specific ω <0.5; 16 of 92 NDGs have branch 0.5<ω <1; and the remaining 24 NDGs have ω >1. Further, LRT tests showed that one NDG has branch specific ω significantly greater than 1. Also, 35 NDGs have branch specific ω significantly smaller than 1. Therefore, branch model ω tests further demonstrated that a large proportion of NDGs are under functional constraints.

We also conducted a comparative analysis between NDGs and randomly selected duplicated genes. We randomly generated 10 data sets of non-redundant (each duplicate gene pair was only picked up once) duplicate gene pairs with each set containing 101 gene pairs, which satisfied the peptide sequence identity of the two genes ≥30%, and alignment coverage of the two proteins ≥70%. We computed the Ka/Ks for the 10 data sets and removed the outliers with Ks >5 whose substitutions are saturated (as shown in Table S4). The comparisons between NDGs and randomly selected duplicated genes suggested that NDGs originated more recently than most of random selected duplicate genes, as shown by lower average Ks, Ka values of the NDGs, and a higher number of cases with Ka and Ks = 0 of the NDGs. Larger number of NDGs were under positive selection as shown by higher number of the gene pairs with Ka/Ks >1. And NDGs may be under relaxed functional constraints, as shown by lower number of the NDG pairs with Ka/Ks significantly less than 0.5 and 1.

### Population Genetic Analysis of *A. thaliana* NDGs

To perform population genetics analysis, we collected SNPs for NDGs and their parental genes across 80 *A. thaliana* accessions. Of the 100 duplicate paralogs with clear origination relationship, in which the parental genes share orthologs and/or syntenic regions with other species and the NDGs are *A. thaliana* lineage specific, 67 NDGs and 68 parental genes have SNP data available, respectively. We computed the average nucleotide polymorphism (θ) and average nucleotide diversity (π) for all sites, synonymous sites, and non-synonymous sites, respectively. The averaged θ and π for NDGs were larger than those for parental genes in all sites, synonymous sites, and non-synonymous sites (except π values at synonymous sites for NDGs were smaller than those for parental genes. Table 2), suggesting the NDGs were evolving more rapidly than their parental genes. To further test whether elevated evolution rate of NDGs resulted from natural selection rather than a random process due to demographic effects, we compared polymorphism patterns between NDGs and randomly selected genes. We generated 10 gene datasets. In each gene dataset, we randomly picked up 100 non-redundant (each gene was picked up once) functional (no pseudogene) annotated *A. thaliana* genes and computed their population genetic statistics as shown in Table S5. We found the π_n_ (π value at the non-synonymous sites) and θ_n_ (θ value at non-synonymous sites) of the NDGs were larger than the randomly selected genes, suggesting the NDGs have a faster evolution rate. We conducted t-tests for θ and π between synonymous sites and non-synonymous sites of NDGs. We demonstrated that θ and π values for non-synonymous sites (θ_n_ and π_n_) were significantly smaller than those for synonymous sites (θ_s_ and π_s_), further indicating that these NDGs were under functional constraints (*p* value for θ_s_ vs. θ_n_ is 4.16E-09, and *p* value for π_s_ vs. π_n_ is 4.57E-07).

**Table 2:** The average values of π and θ for all the sites, synonymous and non-synonymous sites of NDGs and parental genes.

Average value	π_a_	π_s_	π_n_	θ_a_	θ_s_	θ_n_
NDG	0.0054	0.0069	0.0039	0.0082	0.0101	0.0063
Parental gene	0.0049	0.0071	0.0029	0.0071	0.0094	0.0047

π_a_ and θ_a_ for all sites; π_s_ and θ_s_ for synonymous sites; π_n_ and θ_n_ for non-synonymous sites.

To test whether the evolution of these NDGs was driven by natural selection, we conducted Tajima’s D test, Fu & Li’s F and D test, and MK test for all sites. We compared the three neutrality test results, namely Tajima’s D, Fu & Li’s F and D, of each NDG and its parental gene with the empirical distribution of ∼800 independent and randomly sampled genes across the genome to compute the ‘p’ values. If the skewed pattern (e.g. Tajima’s D) detected in a single NDG or its parental gene significantly deviated from the corresponding empirical distribution, it implied that this gene is most likely under positive selection rather than a genome-wide effect and we could eliminate the effect of population structure and demographic history on these tests. We computed the corresponding FDR ‘q’ value for each ‘p’ value and applied FDR ‘q’ value <0.05 to correct for the multiple-testing problem. To define whether a gene is driven by positive selection, we required the Tajima’s D test or Fu & Li’s F and D test values be negative and the ‘q’ values of these tests less than 0.05. Ten of the 67 (14.9%) NDGs, which have SNP data available, had at least one test which significantly deviated from neutrality (Table 3 and Table S6). Six of the 68 (8.8%) parental genes, which have SNP data available, had at least one test that significantly differed from neutrality. We looked at the corresponding parental genes of these 10 NDGs and found that none of these parental genes showed even one selective signature using these tests. Also, six NDGs had the ‘q’ value of MK test smaller than 0.05, and only one parental genes has the ‘q’ value less than 0.05. None of the parental genes corresponding to these six NDGs produced an MK test ‘q’ value smaller than 0.05. The significant ‘q’ value of the MK test can be due to the strong positive selection driving the divergence between the NDGs and the parental genes, or strong purifying selection deleting more polymorphisms from the NDGs than those of the parental genes [80]. If the observed patterns were due to stronger purifying selection deleting more polymorphisms of the NDGs, it would be expected that θ_n_ and π_n_ of the NDGs should be less than those of the parental genes. However, we found that the majority of the six NDGs had higher θ_n_ and π_n_ than most of the parental genes. Thus, our observed pattern should not be due to the polymorphism deletion by stronger purifying selection on the NDGs but due to the fixed divergence by stronger positive selection on the NDGs. Further, the significant MK test can exclude the effect of demographic changes and suggested that the evolution of the six NDGs were driven by positive selection. Overall, by comparing the selection pattern of the NDGs to that of the parental genes, we concluded that the NDGs experienced divergent evolution patterns from the parental genes.

**Table 3:** The number of NDGs showing selective signatures under population genetic tests.

Tajima’s D	Fu and Li’s F	Fu and Li’s D	MK test	# of gene
+	+	+	+	0
+	+	+	−	1
+	+	−	+	1
+	−	+	+	0
−	+	+	+	1
+	+	−	−	1
+	−	+	−	0
+	−	−	+	0
−	+	+	−	1
−	+	−	+	0
−	−	+	+	0
+	−	−	−	0
−	+	−	−	1
−	−	+	−	0
−	−	−	+	4

“+” yes; “−” no.

### Expression Analysis of *A. thaliana* Lineage Specific NDGs

To test whether sub-functionalization and neo-functionalization play roles in the evolution of *A. thaliana* lineage specific NDGs, we examined the expression pattern of 100 NDGs and their parental genes. Overall, 31 NDGs and 41 parental genes have EST data in GenBank (Table 1). 69 NDGs and 65 parental genes have UniGene annotation (Table 1). The presence of ESTs in UniGene allowed us to detect tissue specific profiles of mRNA accumulation. As shown in UniGene Profile Viewer [81], 24 of 69 NDGs had a tissue specific expression pattern. Furthermore, statistical analysis indicated 14 NDGs were significantly associated with ESTs derived from one tissue (Figure S5). By comparing the expression profiles of 17 paralogs that contained expression data in both NDGs and parental genes, we observed 10 NDGs showing expression patterns differing from their parental genes. For example, the inflorescence enriched NDG *At1g74290* came from the seed and root enriched parental gene *At1g74280*. The cell culture and flower enriched NDG *At2g04390* was changed from the root enriched parental gene *At5g04800*. The flower enriched NDG *At3g49420*, vegetative tissue enriched NDG *At4g21460* and the root enriched NDG *At3g05160* and *AT3G23510* came from parental genes which had non-specific expression. The root enriched parental gene *At4g23430*, the flower enriched parental gene *At2g05310*, the silique enriched parental gene *At5g25757*, and the bud enriched parental gene *At2g16530* gave rise to the non-specific NDGs *At4g23420*, *At4g13500*, *At5g25754*, and *At1g72590*, respectively.

We observed similar changes in expression patterns between NDGs and their parental genes using tilling array expression data. Overall, we extracted expression data for 62 NDGs and 62 parental genes from the tiling array expression data at http://genomebiology.com/2008/9/7/R112/additional/ (Tables S7 and S8) [73]. According to Z-score of the expression data based on tiling array, 11 NDGs and 7 parental genes were tissue-specifically expressed. We further detected 7 NDGs that were expressed differently to their parental genes. For example, four NDGs changed from non-tissue specific parental genes to root-specific; expression of NDG *At4g10860* was senescing-leaf specific compared to non-tissue specific expression of parental gene. Two parental genes with seedling specific and expanding-leaf specific changed to non-tissue specific in NDGs *At2g43440* and *At1g31670* (Tables S7 and S8).

We detected MPSS mRNA for 28 NDGs and 36 parental genes in 17 libraries. 25 of 28 NDGs and 34 of 36 parental genes expressed mRNA enrichment in at least one tissue (Tables S9 and S10). We examined the mRNA enrichment pattern for 17 paralog pairs that have MPSS mRNA data for both NDGs and parental genes. We identified that 11 of these 17 NDGs had different mRNA enrichment pattern compared to their parental genes (Table S9 and S10). 70 NDGs and 73 parental had small RNA data from 40 libraries (Tables S11 and S12).

We also analyzed the RNA-seq data from three tissues including seedling, root and flower bud for the 100 NDG and their parental genes. We found 74 of 100 gene pairs had both parental genes and NDGs expressed in at least one of the three tissues. Twenty NDGs and 14 parental genes were expressed in none of the three tissues. We identified that 2 of 75 gene pairs had NDGs with different expression pattern from the parental genes (Table S13). The NDG *At1g31670* changed from seedling specific parental gene *At1g31690* to non-tissue specific. The NDG *At3g02240* changed from non-tissue specific parental gene *At3g02242* to seedling specific.

In summary, all 100 NDGs were demonstrated as being transcribed from at least one expression data set (Table S14). The expression for 91 of 100 NDGs was supported by two or more expression data sources (Table S14). 45 NDGs had enriched expression in certain tissues. Among them, 24 NDGs were statistically significant in tissue-specific expression. 24 of 100 paralogs with expression data available for both NDGs and parental genes showed divergent expression patterns between NDGs and parental genes, indicating sub-functionalization or neo-functionalization (Table 4). We further examined the divergent functionalities of four NDGs based on the asymmetric expression and their physiological effects. (1) *At4g12620* and *At4g14700* have unrelated promoters. The parental gene, *At4g12620,* is restrictively expressed in proliferating cells while the NDG, *At4g14700*, is preferentially found in endoreplicating cells [82]. (2) Although the histochemical staining and GUS activity measurement suggested *At1g07780* (the parental gene) and *At1g29410* (the NDG) transgenic plants have similar expression levels and patterns, no functional *At1g29410* cDNA clones were found by using a functional complementation test [83]. (3) *At1g19080* (the NDG) was found to change in gene expression during pollen germination and tube growth [84] and played a role in embryo development [85], however *Ag3g55490* (the parental genes) did not share this pattern. (4) *At3g05160* (the NDG) has been demonstrated to play a part in an auxin regulatory circuit involved in the control of a hypo-sulphur stress [86], while *At3g05165* (the parental gene) has been found to change in gene expression during pollen germination and tube growth [84].

**Table 4:** The 24 paralog pairs having differential expression pattern between NDGs and parental genes.

NDG	Parental gene	Ka	NDG enriched tissue	Parental gene enriched tissue	Data source
*At1g19080*	*At3g55490*	0	Leave	Non specific	MPSS
*At1g29410*	*At1g07780*	0.1411	Silique	Inflorescence	MPSS
*At1g52270*	*At4g28310*	0.1369	Non specific	Root	MPSS
*At1g74290*	*At1g74280*	0.0549	Non specific	Root	MPSS
*At1g80700*	*At1g80980*	0.0019	Root	Inflorescence	MPSS
*At2g09990*	*At5g18380*	0.0029	Inflorescence	Seedlings	MPSS
*At4g14700*	*At4g12620*	0.0482	Inflorescence	Silique	MPSS
*At5g28900*	*At5g28850*	0.0015	Callus	Callus and root	MPSS
*At5g43620*	*At1g66500*	0.0363	Non specific	Callus	MPSS
*At1g21530*	*At1g21540*	0.0572	Root-specific	Non specific	Tiling array
*At1g29830*	*At1g29820*	0.079	Root-specific	Non specific	Tiling array
*At1g31670*	*At1g31690*	0.0978	Non specific	Expanding-leave specific/seedling	Tiling array/RNA-seq
*At2g43440*	*At2g43445*	0.0792	Non specific	Seedling specific	Tiling array
*At3g23510*	*At3g23530*	0.0138	Root-specific	Non specific	Tiling array
*At4g10860*	*At4g10880*	0.1353	Senescing-leave specific	Non specific	Tiling array
*At1g72590*	*At2g16530*	0.0582	Non specific	Bud	Unigene
*At2g04390*	*At5g04800*	0.0098	Cell culture	Root	Unigene
*At3g05160*	*At3g05165*	0.1104	Root	Non specific	Unigene
*At4g13500*	*At2g05310*	0.0337	Non specific	Flower	Unigene
*At5g25754*	*At5g25757*	0	Non specific	Silique	Unigene
*At3g49420*	*At5g01430*	0	Flower/Callus	Non specific	Unigene/MPSS
*At4g21460*	*At3g18240*	0.0244	Vegetative/Inflorescence	Non specific/callus	Unigene/MPSS
*At4g23420*	*At4g23430*	0.0513	Non-specific/seedling	Root/callus	Unigene/MPSS
*At3g02240*	*At3g02242*	0.2633	Seedling	Non specific	RNA-seq

### The Methylation Pattern of NDGs

We examined the degree of methylation conservation between NDGs and their parental genes in gene body. We also examined and compared the methylation level for NDGs and their parental genes in promoter regions (200 bp upstream of the transcriptional start sites) and transcriptional termination regions (200 bp downstream of the transcriptional termination site) [79]. We found 17 paralogs that had significantly low methylation conservation in gene body between the NDGs and parental genes compared with the methylation conservation of all the duplicated genes (binomial test with correcting multiple testing with FDR <0.05, Table S15). We found 5 paralogs which had different methylation levels in promoter regions between NDG and their parental genes. Three NDGs (*At1g30974, At1g45190, At2g13450*) showed higher methylation levels in the promoters and two parental genes (*At4g04030, At4g34080*) showed higher methylation levels in the promoters compared to the common methylation level in the promoters of all the genes (binomial test with correcting multiple testing with FDR <0.05).

### The Cis-regulatory Motif Pattern of NDGs

In addition to methylation pattern, we analyzed the *cis*-regulatory elements annotated on the 100 gene pairs. The data was downloaded from AGRIS http://arabidopsis.med.ohio-state.edu/downloads.html. 32 of our NDGs and parental genes had annotated *cis*-regulatory elements. Only 2 NDG possessed the same *cis* regulatory element as the parental gene, the majority of NDGs and their parental genes had divergent *cis*-elements: (1) Seven parental genes had additional unique *cis* regulatory elements besides the ones shared with the NDGs. (2) Two NDGs had additional unique *cis* regulatory elements besides the ones shared with the parental genes, (3) 21 pairs of NDGs and parental genes had different *cis* regulatory elements (Table S16). Among 24 paralogous gene pairs whose NDG and parental gene showed divergent expression patterns, 21 paralogous gene pairs had both parental gene and NDG annotated with *cis* regulatory elements. All these 21 paralogous gene pairs showed *cis*-elements divergence: (1) One parental gene had additional unique *cis* regulatory elements besides the ones shared with the NDG. (2) Three NDGs had additional unique *cis* regulatory elements besides the ones shared with the parental genes. (3) 17 pairs of NDG and parental gene had different *cis* regulatory elements.

## Discussion

### The Rapid Origination Rate of NDGs in *A. thaliana*

Gene duplication is a profound phenomenon in plant genome evolution. Using rigorous comparative genomics analysis, among closely related species, we identified 137 *A. thaliana* lineage specific duplicate genes accounted for 0.50% of *A. thaliana’s* total 27,416 protein-coding genes. The rate of duplicate genes in *Arabidopsis* (14∼27 duplication events/million years) is three fold higher than that in any animal species measured to date [26], [74], [87], [88]. This suggests that *Arabidopsis* genomes could have been shaped by a rapid evolution of duplicate genes as an adaptation to highly diverse environments.

However, compared with a previous study by Donoghue [38], which identified 417 *A. thaliana* lineage specific genes originating from duplication, 225 of them with significant BLASTP hits to a non-lineage specific genes and 180 with expression data support, these numbers from our analysis are reduced to 137, 100, and 100, respectively. This could be due to that we used both syntenic map and BLASTP search to identify orthologs. This combined approach increased the number of orthologs and thus decreased the number of lineage specific genes. Donoghue et al also used position-specific method, namely Position-Specific Iterated BLAST (PSIBLAST), to detect homologs. However, compared to the position-specific method, syntenic map approach based on whole genome comparison is likely to reveal more comprehensive orthologous information than PSIBLAST.

### Natural Selection Drives the Evolution of NDGs

The process by which duplicate genes evolve and become fixed in a genome is one of the central questions in molecular evolution [33]. When effective population size (*N_e_*) is small, a duplicate gene with neutral or slightly deleterious mutations may become fixed in the population due to genetic drift [89], [90]. In addition, the selectively neutral “duplication-degeneration-complementation” (DDC) model leading to a neutral sub-functionalization, hypothesized that both gene copies can be maintained in the genome due to complementary degenerate mutations. This process distributed the functionality of the original genes between the two duplicate copies through neutral mutations [30], [37], [91], [92]. Both models suggest that the lineage specific duplicate genes should be the product of passive fixation of gene duplication especially in the species with small *N_e_* rather than the product of positive adaptation to the environment.

In contrast, many empirical examples and theoretical studies demonstrated that the evolution of duplicate genes is driven by positive selection resulting in either sub-functionalization or neo-functionalization [93]–[96]. The classical escape from adaptive conflict (EAC) model leading to EAC sub-functionalization suggests that two genes can have specialized expressions in different tissues or different development stages [30], [97]. This model is different from DDC in that function is developed through adaptive (non-neutral) mutations. The EAC sub-functionalization model, involving selection, holds that multiple functions of the ancestral gene cannot be optimized at the same time by natural selection. After gene duplication, the two daughter genes can avoid this conflict through experiencing adaptive mutations, which leads them to specializing in different functions within the original set of functions thereby increasing the fitness of the organism [30], [98], [99]. Neo-functionalization occurs when one duplicate retains the original function and the other duplicate copy evolves a novel function [33]. Both EAC sub-functionalization and neo-functionalization involve duplicate genes evolving driven by natural selection.

*Arabidopsis thaliana* is a selfing plant species with relatively small *N_e_*. Previous studies reported its *N_e_* ranges from a few to a few thousands [100], [101]. To test whether NDGs identified were under functional constraints and were evolved under natural selection, we estimated their Ka/Ks ratio, conducted the ‘t’ test for the rate of substitution pattern and analyzed SNP data with various population genetics tests. We estimated that most of NDGs in *A. thaliana* were under functional constraint. Thus, neutral and/or slight deleterious mutation to NDGs and genetic drift due to small *N_e_* might not be able to explain the whole picture of the NDGs evolution in *A. thaliana.* Further, our polymorphism analysis showed that about 15% of the NDGs (10 out of 67 NDGs) with clear origination relationship and SNP data had a positive selection signature, revealing that the evolution of a large proportion of the NDGs in *A. thaliana* were driven by natural selection. Interestingly, when compared to their parental genes, evidence showed that 3 of the 24 NDGs that switched their tissue expression specificity also displayed selection signatures (Table S17). Moreover, all the three NDGs (Table S17) involved important biological functions in *A. thaliana*, suggesting that they might play an important role in the adaptation of *A. thaliana*, driven by natural selection.

### The Possible Mechanisms Causing the Divergent Expression Patterns of NDGs

Gene duplication is one of the most important mechanisms to generate biological diversity. In our studies, with available data from four data sources, we found 24 NDGs that showed expression patterns different from their parental genes (Table 3). Eight of 24 (∼33%) NDGs changed from non-tissue specific parental genes to certain tissue specific genes, and 7 out of the 8 genes changed to vegetative tissues (e.g. root and leaf). This was different from what was observed in fruit fly, silkworm and mammals where the NDGs through retrotransposition mechanisms tended to be expressed in male testis [60], [72], [102]–[104], or NDGs tended to be expressed in nervous systems in mammals [5], [105]. Surprisingly, the rate of nonsynonymous substitution between these 24 NDGs and their parental genes were very small with the average Ka of 0.0599 (Table 4). In addition to the replacement substitutions in coding regions, these NDGs may acquire differential expression patterns from their parental genes by obtaining new *trans-* or *cis-* regulatory motifs [106], or epigenetic regulation by change of methylation status [107], [108], as we showed in the results. Thus, the epigenetic and *cis*-regulatory pattern may play a role in driving the differential expression of the 24 NDGs from their parental genes.

### The Small-scale Gene Duplications have Higher Chance to Develop Divergent Expression Pattern

To test if the duplication mechanism is correlated with divergent expression pattern, we examined the expression pattern of NDGs derived from small-scale gene duplication (tandem or dispersed duplication) and large-scale gene duplication (segmental duplication). All 24 paralogous gene pairs of which the NDGs exhibited asymmetric expression pattern from the parental genes were derived through either tandem duplication or dispersed duplication. We further examined the *cis*-elements of 100 pairs of NDGs and parental genes. For the 32 gene pairs with both the parental gene and NDG having *cis* regulatory motif annotated, regardless of the motifs being the same or different between the two paralogous genes, all NDGs were generated from either tandem duplication or dispersed duplication. This conclusion is consistent with that of previous studies that small-scale duplication events have higher potential to generate the NDGs with different expression/function from the parental genes than do the large-scale duplication events [109].

## Supporting Information

Figure S1 **Illustration of segmental duplication.**
(TIF)

Figure S2 **Tandem duplication defined by synteny.**
(TIF)

Figure S3 **Tandem duplication defined phylogenetic analysis.**
(TIF)

Figure S4 **Example of emipirical distribution of Tajama’s D statistic values obtaied from a large data set.** The red line indicates the Tajima’s D values from a single NDG.
(TIF)

Figure S5 **The EST expression profile of 30 new genes and 33 parental genes from UniGene Profile Viewer.**
(PDF)

Table S1 **137 lineage-specific duplicated genes.**
(PDF)

Table S2 **Lineage-specific chimeric duplicated genes.**
(PDF)

Table S3 **92 NDG branch specific Ka/Ks and background Ka/Ks.**
(PDF)

Table S4 **Comparison of the Ka, Ks, and Ka/Ks values between NDGs and 10 simulated duplicated gene datasets.**
(PDF)

Table S5 **The population genetics statistics of 10 datasets of 100 duplicated genes.**
(PDF)

Table S6 **Ten new genes with selection signature.**
(PDF)

Table S7 **Tiling array data of 62 new genes.**
(PDF)

Table S8 **Tiling array data of 62 parental genes.**
(PDF)

Table S9 **The MPSS data of 100 new genes.**
(PDF)

Table S10 **The MPSS data of 100 old genes.**
(PDF)

Table S11 **Small RNA data of 100 new genes.**
(PDF)

Table S12 **Small RNA data of 100 old genes.**
(PDF)

Table S13 **RNA-seq data for 100 duplicated gene pairs.**
(PDF)

Table S14 **Gene expression sources of 100 new genes.**
(PDF)

Table S15 **17 gene pairs with low methylation conservation.**
(PDF)

Table S16 **The different cis-motifs in the 32 gene pairs with cis-motifs available for new genes and parental genes.**
(PDF)

Table S17 **Three new genes that not only switch their tissue expression specificity but also show selection signature.**
(PDF)

## References

[1] Khalturin K, Hemmrich G, Fraune S, Augustin R, Bosch TCG (2009) More than just orphans: are taxonomically-restricted genes important in evolution? Trends in Genetics 25: 404–413. 19716618 10.1016/j.tig.2009.07.006

[2] Siew N, Fischer D (2003) Unravelling the ORFan puzzle. Comparative and Functional Genomics 4: 432–441. 18629076 10.1002/cfg.311 PMC2447361

[3] Tautz D, Domazet-Loso T (2011) The evolutionary origin of orphan genes. Nat Rev Genet 12: 692–702. 21878963 10.1038/nrg3053

[4] Chen S, Zhang YE, Long M (2010) New genes in Drosophila quickly become essential. Science (New York, N Y ) 330: 1682–1685. 10.1126/science.1196380 PMC7211344 21164016

[5] Zhang YE, Landback P, Vibranovski MD, Long M (2011) Accelerated recruitment of new brain development genes into the human genome. PLoS Biol 9: e1001179. 22028629 10.1371/journal.pbio.1001179 PMC3196496

[6] Ding Y, Zhao L, Yang S, Jiang Y, Chen Y, et al (2010) A young Drosophila duplicate gene plays essential roles in spermatogenesis by regulating several Y-linked male fertility genes. PLoS Genetics 6: e1001255. 21203494 10.1371/journal.pgen.1001255 PMC3009665

[7] Zhang J, Zhang Y-p, Rosenberg HF (2002) Adaptive evolution of a duplicated pancreatic ribonuclease gene in a leaf-eating monkey. Nat Genet 30: 411–415. 11925567 10.1038/ng852

[8] Weng JK, Li Y, Mo H, Chapple C (2012) Assembly of an evolutionarily new pathway for alpha-pyrone biosynthesis in Arabidopsis. Science 337: 960–964. 22923580 10.1126/science.1221614

[9] Kliebenstein DJ (2008) A role for gene duplication and natural variation of gene expression in the evolution of metabolism. PLoS One 3: e1838. 18350173 10.1371/journal.pone.0001838 PMC2263126

[10] Ferrari S, Vairo D, Ausubel FM, Cervone F, De Lorenzo G (2003) Tandemly duplicated Arabidopsis genes that encode polygalacturonase-inhibiting proteins are regulated coordinately by different signal transduction pathways in response to fungal infection. Plant Cell 15: 93–106. 12509524 10.1105/tpc.005165 PMC143454

[11] Duarte JM, Cui L, Wall PK, Zhang Q, Zhang X, et al (2006) Expression pattern shifts following duplication indicative of subfunctionalization and neofunctionalization in regulatory genes of Arabidopsis. Mol Biol Evol 23: 469–478. 16280546 10.1093/molbev/msj051

[12] Hanada K, Zou C, Lehti-Shiu MD, Shinozaki K, Shiu SH (2008) Importance of lineage-specific expansion of plant tandem duplicates in the adaptive response to environmental stimuli. Plant Physiol 148: 993–1003. 18715958 10.1104/pp.108.122457 PMC2556807

[13] Bikard D, Patel D, Le Mette C, Giorgi V, Camilleri C, et al (2009) Divergent evolution of duplicate genes leads to genetic incompatibilities within A. thaliana. Science 323: 623–626. 19179528 10.1126/science.1165917

[14] Zou C, Lehti-Shiu MD, Thomashow M, Shiu SH (2009) Evolution of stress-regulated gene expression in duplicate genes of Arabidopsis thaliana. PLoS Genet 5: e1000581. 19649161 10.1371/journal.pgen.1000581 PMC2709438

[15] Cai JJ, Petrov DA (2010) Relaxed Purifying Selection and Possibly High Rate of Adaptation in Primate Lineage-Specific Genes. Genome Biol Evol 2: 393–409. 20624743 10.1093/gbe/evq019 PMC2997544

[16] Tay SK, Blythe J, Lipovich L (2009) Global discovery of primate-specific genes in the human genome. Proc Natl Acad Sci U S A 106: 12019–12024. 19581580 10.1073/pnas.0904569106 PMC2715485

[17] Schmid KJ, Aquadro CF (2001) The evolutionary analysis of “orphans” from the Drosophila genome identifies rapidly diverging and incorrectly annotated genes. Genetics 159: 589–598. 11606536 10.1093/genetics/159.2.589 PMC1461820

[18] Guo WJ, Li P, Ling J, Ye SP (2007) Significant comparative characteristics between orphan and nonorphan genes in the rice (Oryza sativa L.) genome. Comparative and Functional Genomics. 10.1155/2007/21676 PMC2216055 18273382

[19] Wendel JF (2000) Genome evolution in polyploids. Plant Mol Biol 42: 225–249. 10688139

[20] Vision TJ, Brown DG, Tanksley SD (2000) The origins of genomic duplications in Arabidopsis. Science 290: 2114–2117. 11118139 10.1126/science.290.5499.2114

[21] Blanc G, Hokamp K, Wolfe KH (2003) A recent polyploidy superimposed on older large-scale duplications in the Arabidopsis genome. Genome Res 13: 137–144. 12566392 10.1101/gr.751803 PMC420368

[22] Bekaert M, Edger PP, Pires JC, Conant GC (2011) Two-Phase Resolution of Polyploidy in the Arabidopsis Metabolic Network Gives Rise to Relative and Absolute Dosage Constraints. Plant Cell 23: 1719–1728. 21540436 10.1105/tpc.110.081281 PMC3123947

[23] Hudson CM, Puckett EE, Bekaert M, Pires JC, Conant GC (2011) Selection for Higher Gene Copy Number after Different Types of Plant Gene Duplications. Genome Biology and Evolution 3: 1369–1380. 22056313 10.1093/gbe/evr115 PMC3240960

[24] Fan C, Vibranovski MD, Chen Y, Long M (2007) A Microarray Based Genomic Hybridization Method for Identification of New Genes in Plants: Case Analyses of Arabidopsis and Oryza. Journal of Integrative Plant Biology 49.

[25] Zhang YJ, Wu YR, Liu YL, Han B (2005) Computational identification of 69 retroposons in Arabidopsis. Plant Physiol 138: 935–948. 15923328 10.1104/pp.105.060244 PMC1150409

[26] Wang W, Zheng H, Fan C, Li J, Shi J, et al (2006) High rate of chimeric gene origination by retroposition in plant genomes. Plant Cell 18: 1791–1802. 16829590 10.1105/tpc.106.041905 PMC1533979

[27] Rizzon C, Ponger L, Gaut BS (2006) Striking similarities in the genomic distribution of tandemly arrayed genes in Arabidopsis and rice. PLoS Comput Biol 2: e115. 16948529 10.1371/journal.pcbi.0020115 PMC1557586

[28] Kaessmann H, Vinckenbosch N, Long M (2009) RNA-based gene duplication: mechanistic and evolutionary insights. Nat Rev Genet 10: 19–31. 19030023 10.1038/nrg2487 PMC3690669

[29] Long M, Betran E, Thornton K, Wang W (2003) The origin of new genes: glimpses from the young and old. Nat Rev Genet 4: 865–875. 14634634 10.1038/nrg1204

[30] Conant GC, Wolfe KH (2008) Turning a hobby into a job: how duplicated genes find new functions. Nat Rev Genet 9: 938–950. 19015656 10.1038/nrg2482

[31] Cardoso-Moreira M, Long M (2012) The origin and evolution of new genes. Methods Mol Biol 856: 161–186. 22399459 10.1007/978-1-61779-585-5_7

[32] Ranz JM, Parsch J (2012) Newly evolved genes: Moving from comparative genomics to functional studies in model systems: How important is genetic novelty for species adaptation and diversification? Bioessays. 10.1002/bies.201100177 22461005

[33] Ohno S (1970) Evolution by gene duplication. Berlin, New York,: Springer-Verlag. xv, 160 p. p.

[34] Ohta T (1989) Role of gene duplication in evolution. Genome 31: 304–310. 2687099 10.1139/g89-048

[35] Lynch M, Conery JS (2000) The evolutionary fate and consequences of duplicate genes. Science 290: 1151–1155. 11073452 10.1126/science.290.5494.1151

[36] Maere S, De Bodt S, Raes J, Casneuf T, Van Montagu M, et al (2005) Modeling gene and genome duplications in eukaryotes. Proc Natl Acad Sci U S A 102: 5454–5459. 15800040 10.1073/pnas.0501102102 PMC556253

[37] Force A, Lynch M, Pickett FB, Amores A, Yan YL, et al (1999) Preservation of duplicate genes by complementary, degenerative mutations. Genetics 151: 1531–1545. 10101175 10.1093/genetics/151.4.1531 PMC1460548

[38] Donoghue MT, Keshavaiah C, Swamidatta SH, Spillane C (2011) Evolutionary origins of Brassicaceae specific genes in Arabidopsis thaliana. BMC Evol Biol 11: 47. 21332978 10.1186/1471-2148-11-47 PMC3049755

[39] Lin HN, Moghe G, Ouyang S, Iezzoni A, Shiu SH, et al.. (2010) Comparative analyses reveal distinct sets of lineage-specific genes within Arabidopsis thaliana. BMC Evol Biol 10. 10.1186/1471-2148-10-41 PMC2829037 20152032

[40] Yang X, Jawdy S, Tschaplinski TJ, Tuskan GA (2009) Genome-wide identification of lineage-specific genes in Arabidopsis, Oryza and Populus. Genomics 93: 473–480. 19442640 10.1016/j.ygeno.2009.01.002

[41] Ha M, Kim ED, Chen ZJ (2009) Duplicate genes increase expression diversity in closely related species and allopolyploids. Proc Natl Acad Sci U S A 106: 2295–2300. 19168631 10.1073/pnas.0807350106 PMC2650150

[42] Ganko EW, Meyers BC, Vision TJ (2007) Divergence in expression between duplicated genes in Arabidopsis. Mol Biol Evol 24: 2298–2309. 17670808 10.1093/molbev/msm158

[43] Casneuf T, De Bodt S, Raes J, Maere S, Van de Peer Y (2006) Nonrandom divergence of gene expression following gene and genome duplications in the flowering plant Arabidopsis thaliana. Genome Biol 7: R13. 16507168 10.1186/gb-2006-7-2-r13 PMC1431724

[44] Slotte T, Hazzouri KM, Agren JA, Koenig D, Maumus F, et al.. (2013) The Capsella rubella genome and the genomic consequences of rapid mating system evolution. Nat Genet. 10.1038/ng.2669 23749190

[45] Arabidopsis Genome Initiative (2000) Analysis of the genome sequence of the flowering plant Arabidopsis thaliana. Nature 408: 796–815. 11130711 10.1038/35048692

[46] Wang X, Wang H, Wang J, Sun R, Wu J, et al (2011) The genome of the mesopolyploid crop species Brassica rapa. Nat Genet 43: 1035–1039. 21873998 10.1038/ng.919

[47] Hu TT, Pattyn P, Bakker EG, Cao J, Cheng JF, et al (2011) The Arabidopsis lyrata genome sequence and the basis of rapid genome size change. Nat Genet 43: 476–481. 21478890 10.1038/ng.807 PMC3083492

[48] Yang YW, Lai KN, Tai PY, Li WH (1999) Rates of nucleotide substitution in angiosperm mitochondrial DNA sequences and dates of divergence between Brassica and other angiosperm lineages. J Mol Evol 48: 597–604. 10198125 10.1007/pl00006502

[49] Town CD, Cheung F, Maiti R, Crabtree J, Haas BJ, et al (2006) Comparative genomics of Brassica oleracea and Arabidopsis thaliana reveal gene loss, fragmentation, and dispersal after polyploidy. Plant Cell 18: 1348–1359. 16632643 10.1105/tpc.106.041665 PMC1475499

[50] Koch MA, Kiefer M (2005) Genome evolution among cruciferous plants: A lecture from the comparison of the genetic maps of three diploid species - Capsella rubella, Arabidopsis lyrata subsp Petraea, and A. thaliana. American Journal of Botany 92: 761–767. 21652456 10.3732/ajb.92.4.761

[51] Wright SI, Lauga B, Charlesworth D (2002) Rates and patterns of molecular evolution in inbred and outbred Arabidopsis. Mol Biol Evol 19: 1407–1420. 12200469 10.1093/oxfordjournals.molbev.a004204

[52] Koch MA, Haubold B, Mitchell-Olds T (2000) Comparative evolutionary analysis of chalcone synthase and alcohol dehydrogenase loci in Arabidopsis, Arabis, and related genera (Brassicaceae). Mol Biol Evol 17: 1483–1498. 11018155 10.1093/oxfordjournals.molbev.a026248

[53] Koch M, Haubold B, Mitchell-Olds T (2001) Molecular systematics of the Brassicaceae: evidence from coding plastidic matK and nuclear Chs sequences. Am J Bot 88: 534–544. 11250830

[54] Kent WJ, Sugnet CW, Furey TS, Roskin KM, Pringle TH, et al (2002) The human genome browser at UCSC. Genome Res 12: 996–1006. 12045153 10.1101/gr.229102 PMC186604

[55] Smit A, Hubley R, Green P (1996–2010) RepeatMasker Open-3.0.

[56] Schwartz S, Kent WJ, Smit A, Zhang Z, Baertsch R, et al (2003) Human-mouse alignments with BLASTZ. Genome Res 13: 103–107. 12529312 10.1101/gr.809403 PMC430961

[57] Altschul SF, Gish W, Miller W, Myers EW, Lipman DJ (1990) Basic local alignment search tool. J Mol Biol 215: 403–410. 2231712 10.1016/S0022-2836(05)80360-2

[58] Kent WJ (2002) BLAT - The BLAST-like alignment tool. Genome Res 12: 656–664. 11932250 10.1101/gr.229202 PMC187518

[59] Yang ZH (2007) PAML 4: Phylogenetic analysis by maximum likelihood. Mol Biol Evol 24: 1586–1591. 17483113 10.1093/molbev/msm088

[60] Betran E, Thornton K, Long M (2002) Retroposed new genes out of the X in Drosophila. Genome Res 12: 1854–1859. 12466289 10.1101/gr.604902 PMC187566

[61] Li W-H (1997) Molecular Evolution. Sunderland Massachusetts: Sinauer Associates.

[62] Katoh K, Kuma K, Toh H, Miyata T (2005) MAFFT version 5: improvement in accuracy of multiple sequence alignment. Nucleic Acids Res 33: 511–518. 15661851 10.1093/nar/gki198 PMC548345

[63] Yang ZH (1998) Likelihood ratio tests for detecting positive selection and application to primate lysozyme evolution. Mol Biol Evol 15: 568–573. 9580986 10.1093/oxfordjournals.molbev.a025957

[64] Ranwez V, Harispe S, Delsuc F, Douzery EJ (2011) MACSE: Multiple Alignment of Coding SEquences accounting for frameshifts and stop codons. PLoS One 6: e22594. 21949676 10.1371/journal.pone.0022594 PMC3174933

[65] Cao J, Schneeberger K, Ossowski S, Gunther T, Bender S, et al (2011) Whole-genome sequencing of multiple Arabidopsis thaliana populations. Nat Genet 43: 956–963. 21874002 10.1038/ng.911

[66] Tajima F (1989) Statistical-Method for Testing the Neutral Mutation Hypothesis by DNA Polymorphism. Genetics 123: 585–595. 2513255 10.1093/genetics/123.3.585 PMC1203831

[67] Fu YX, Li WH (1993) Statistical tests of neutrality of mutations. Genetics 133: 693–709. 8454210 10.1093/genetics/133.3.693 PMC1205353

[68] Ramos-Onsins SE, Puerma E, Balana-Alcaide D, Salguero D, Aguade M (2008) Multilocus analysis of variation using a large empirical data set: phenylpropanoid pathway genes in Arabidopsis thaliana. Molecular Ecology 17: 1211–1223. 18221273 10.1111/j.1365-294X.2007.03633.x

[69] Nordborg M, Hu TT, Ishino Y, Jhaveri J, Toomajian C, et al (2005) The pattern of polymorphism in Arabidopsis thaliana. Plos Biology 3: 1289–1299. 10.1371/journal.pbio.0030196 PMC1135296 15907155

[70] Nordborg M, Hu TT, Ishino Y, Jhaveri J, Toomajian C, et al (2005) The pattern of polymorphism in Arabidopsis thaliana. PLoS Biol 3: e196. 15907155 10.1371/journal.pbio.0030196 PMC1135296

[71] McDonald JH, Kreitman M (1991) Adaptive protein evolution at the Adh locus in Drosophila. Nature 351: 652–654. 1904993 10.1038/351652a0

[72] Wang J, Long M, Vibranovski MD (2012) Retrogenes moved out of the z chromosome in the silkworm. J Mol Evol 74: 113–126. 22535494 10.1007/s00239-012-9499-y

[73] Laubinger S, Zeller G, Henz SR, Sachsenberg T, Widmer CK, et al (2008) At-TAX: a whole genome tiling array resource for developmental expression analysis and transcript identification in Arabidopsis thaliana. Genome Biol 9: R112. 18613972 10.1186/gb-2008-9-7-r112 PMC2530869

[74] Gan X, Stegle O, Behr J, Steffen JG, Drewe P, et al (2011) Multiple reference genomes and transcriptomes for Arabidopsis thaliana. Nature 477: 419–423. 21874022 10.1038/nature10414 PMC4856438

[75] Langmead B, Trapnell C, Pop M, Salzberg SL (2009) Ultrafast and memory-efficient alignment of short DNA sequences to the human genome. Genome Biol 10: R25. 19261174 10.1186/gb-2009-10-3-r25 PMC2690996

[76] Trapnell C, Williams BA, Pertea G, Mortazavi A, Kwan G, et al (2010) Transcript assembly and quantification by RNA-Seq reveals unannotated transcripts and isoform switching during cell differentiation. Nat Biotechnol 28: 511–U174. 20436464 10.1038/nbt.1621 PMC3146043

[77] Lister R, O’Malley RC, Tonti-Filippini J, Gregory BD, Berry CC, et al (2008) Highly integrated single-base resolution maps of the epigenome in Arabidopsis. Cell 133: 523–536. 18423832 10.1016/j.cell.2008.03.029 PMC2723732

[78] Krueger F, Andrews SR (2011) Bismark: a flexible aligner and methylation caller for Bisulfite-Seq applications. Bioinformatics 27: 1571–1572. 21493656 10.1093/bioinformatics/btr167 PMC3102221

[79] Li X, Zhu J, Hu F, Ge S, Ye M, et al (2012) Single-base resolution maps of cultivated and wild rice methylomes and regulatory roles of DNA methylation in plant gene expression. BMC Genomics 13: 300. 22747568 10.1186/1471-2164-13-300 PMC3447678

[80] Hughes AL, Friedman R, Rivailler P, French JO (2008) Synonymous and nonsynonymous polymorphisms versus divergences in bacterial genomes. Mol Biol Evol 25: 2199–2209. 18667439 10.1093/molbev/msn166 PMC2734133

[81] Sayers EW, Barrett T, Benson DA, Bolton E, Bryant SH, et al (2012) Database resources of the National Center for Biotechnology Information. Nucleic Acids Res 40: D13–D25. 22140104 10.1093/nar/gkr1184 PMC3245031

[82] Diaz-Trivino S, del Mar Castellano M, de la Paz Sanchez M, Ramirez-Parra E, Desvoyes B, et al (2005) The genes encoding Arabidopsis ORC subunits are E2F targets and the two ORC1 genes are differently expressed in proliferating and endoreplicating cells. Nucleic Acids Res 33: 5404–5414. 16179646 10.1093/nar/gki854 PMC1236721

[83] He Y, Li J (2001) Differential expression of triplicate phosphoribosylanthranilate isomerase isogenes in the tryptophan biosynthetic pathway of Arabidopsis thaliana (L.) Heynh. Planta 212: 641–647. 11346937 10.1007/s004250000452

[84] Wang Y, Zhang WZ, Song LF, Zou JJ, Su Z, et al (2008) Transcriptome analyses show changes in gene expression to accompany pollen germination and tube growth in Arabidopsis. Plant Physiol 148: 1201–1211. 18775970 10.1104/pp.108.126375 PMC2577266

[85] Tzafrir I, Pena-Muralla R, Dickerman A, Berg M, Rogers R, et al (2004) Identification of genes required for embryo development in Arabidopsis. Plant Physiol 135: 1206–1220. 15266054 10.1104/pp.104.045179 PMC519041

[86] Nikiforova VJ, Daub CO, Hesse H, Willmitzer L, Hoefgen R (2005) Integrative gene-metabolite network with implemented causality deciphers informational fluxes of sulphur stress response. J Exp Bot 56: 1887–1896. 15911562 10.1093/jxb/eri179

[87] Rutter MT, Cross KV, Van Woert PA (2012) Birth, death and subfunctionalization in the Arabidopsis genome. Trends Plant Sci 17: 204–212. 22326563 10.1016/j.tplants.2012.01.006

[88] Zhou Q, Zhang G, Zhang Y, Xu S, Zhao R, et al (2008) On the origin of new genes in Drosophila. Genome Res 18: 1446–1455. 18550802 10.1101/gr.076588.108 PMC2527705

[89] Ohta T (1973) Slightly deleterious mutant substitutions in evolution. Nature 246: 96–98. 4585855 10.1038/246096a0

[90] Ohta T (1992) The nearly neutral theory of molecular evolution. Annu Rev Ecol Syst: 263–286.

[91] Lynch M, O’Hely M, Walsh B, Force A (2001) The probability of preservation of a newly arisen gene duplicate. Genetics 159: 1789–1804. 11779815 10.1093/genetics/159.4.1789 PMC1461922

[92] Lynch M, Conery JS (2003) The origins of genome complexity. Science 302: 1401–1404. 14631042 10.1126/science.1089370

[93] Harada E, Nakagawa J, Asano T, Taoka M, Sorimachi H, et al (2012) Functional evolution of duplicated odorant-binding protein genes, Obp57d and Obp57e, in Drosophila. PLoS One 7: e29710. 22238638 10.1371/journal.pone.0029710 PMC3253112

[94] Proulx SR (2012) Multiple routes to subfunctionalization and gene duplicate specialization. Genetics 190: 737–751. 22143920 10.1534/genetics.111.135590 PMC3276641

[95] Hittinger CT, Carroll SB (2007) Gene duplication and the adaptive evolution of a classic genetic switch. Nature 449: 677–681. 17928853 10.1038/nature06151

[96] Shiu SH, Byrnes JK, Pan R, Zhang P, Li WH (2006) Role of positive selection in the retention of duplicate genes in mammalian genomes. Proc Natl Acad Sci U S A 103: 2232–2236. 16461903 10.1073/pnas.0510388103 PMC1413713

[97] Ferris SD, Whitt GS (1979) Evolution of the differential regulation of duplicate genes after polyploidization. J Mol Evol 12: 267–317. 448746 10.1007/BF01732026

[98] Hughes AL (1994) The evolution of functionally novel proteins after gene duplication. Proc Biol Sci 256: 119–124. 8029240 10.1098/rspb.1994.0058

[99] Piatigorsky J, Wistow G (1991) The recruitment of crystal-lins: new functions precede gene duplication. Science 252: 1078–1079. 2031181 10.1126/science.252.5009.1078

[100] Lundemo S, Falahati-Anbaran M, Stenoien HK (2009) Seed banks cause elevated generation times and effective population sizes of Arabidopsis thaliana in northern Europe. Mol Ecol 18: 2798–2811. 19500249 10.1111/j.1365-294X.2009.04236.x

[101] Gomaa NH, Montesinos-Navarro A, Alonso-Blanco C, Pico FX (2011) Temporal variation in genetic diversity and effective population size of Mediterranean and subalpine Arabidopsis thaliana populations. Mol Ecol 20: 3540–3554. 21790818 10.1111/j.1365-294X.2011.05193.x

[102] Bai YS, Casola C, Feschotte C, Betran E (2007) Comparative genomics reveals a constant rate of origination and convergent acquisition of functional retrogenes in Drosophila. Genome Biol 8. 10.1186/gb-2007-8-1-r11 PMC1839131 17233920

[103] Emerson JJ, Kaessmann H, Betran E, Long MY (2004) Extensive gene traffic on the mammalian X chromosome. Science 303: 537–540. 14739461 10.1126/science.1090042

[104] Vinckenbosch N, Dupanloup I, Kaessmann H (2006) Evolutionary fate of retroposed gene copies in the human genome. Proc Natl Acad Sci U S A 103: 3220–3225. 16492757 10.1073/pnas.0511307103 PMC1413932

[105] Marques AC, Dupanloup I, Vinckenbosch N, Reymond A, Kaessmann H (2005) Emergence of young human genes after a burst of retroposition in primates. PLoS Biol 3: e357. 16201836 10.1371/journal.pbio.0030357 PMC1251493

[106] Udall JA, Swanson JM, Nettleton D, Percifield RJ, Wendel JF (2006) A novel approach for characterizing expression levels of genes duplicated by polyploidy. Genetics 173: 1823–1827. 16702424 10.1534/genetics.106.058271 PMC1526680

[107] Lukens LN, Zhan SH (2007) The plant genome’s methylation status and response to stress: implications for plant improvement. Current Opinion in Plant Biology 10: 317–322. 17468039 10.1016/j.pbi.2007.04.012

[108] Wang WS, Pan YJ, Zhao XQ, Dwivedi D, Zhu LH, et al (2011) Drought-induced site-specific DNA methylation and its association with drought tolerance in rice (Oryza sativa L.). Journal of Experimental Botany 62: 1951–1960. 21193578 10.1093/jxb/erq391 PMC3060682

[109] Carretero-Paulet L, Fares MA (2012) Evolutionary dynamics and functional specialization of plant paralogs formed by whole and small-scale genome duplications. Mol Biol Evol 29: 3541–3551. 22734049 10.1093/molbev/mss162

