# Supplementary figures and images for: Divergent Evolutionary and Expression Patterns between Lineage Specific New Duplicate Genes and Their Parental Paralogs in Arabidopsis thaliana
Source: PLoS One. 2013 Aug 29;8(8):e72362. doi: 10.1371/journal.pone.0072362 (PMC3756979; doi:10.1371/journal.pone.0072362)

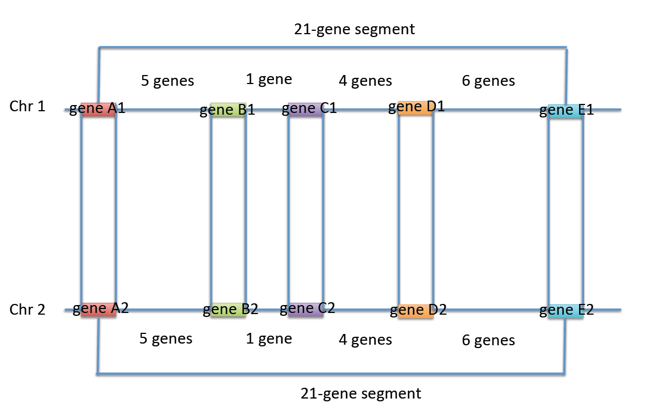

Supplement: Figure S1 — Illustration of segmental duplication. (TIF) [file pone.0072362.s001.tif]

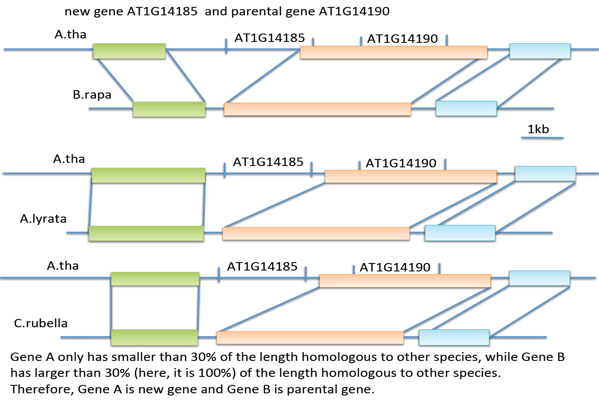

Supplement: Figure S2 — Tandem duplication defined by synteny. (TIF) [file pone.0072362.s002.tif]

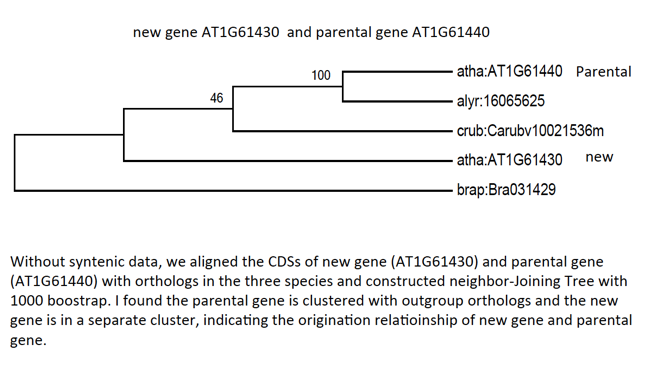

Supplement: Figure S3 — Tandem duplication defined phylogenetic analysis. (TIF) [file pone.0072362.s003.tif]

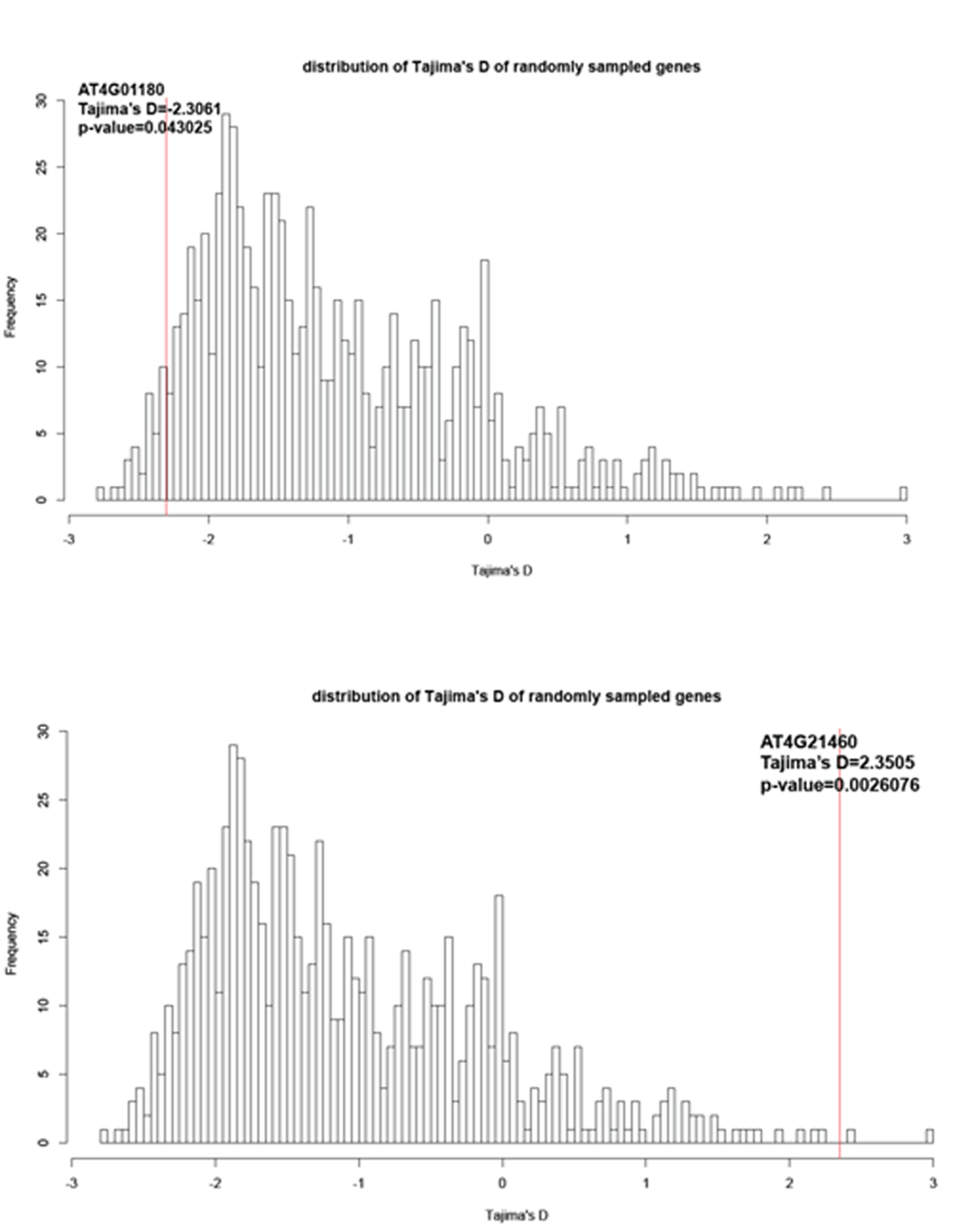

Supplement: Figure S4 — Example of emipirical distribution of Tajama’s D statistic values obtaied from a large data set. The red line indicates the Tajima’s D values from a single NDG. (TIF) [file pone.0072362.s004.tif]
