# Supplementary material for: Divergent Evolutionary and Expression Patterns between Lineage Specific New Duplicate Genes and Their Parental Paralogs in Arabidopsis thaliana
Source: PLoS One. 2013 Aug 29;8(8):e72362. doi: 10.1371/journal.pone.0072362 (PMC3756979; doi:10.1371/journal.pone.0072362)
Supplement: Figure S5 — The EST expression profile of 30 new genes and 33 parental genes from UniGene Profile Viewer. (PDF) [file pone.0072362.s005.pdf]

New gene

AT1G24880 p-value = 8.947e-15(bud)

AT1G25054

At.48022

bud	2540	16 / 6299
cell culture	0	0 / 1100
floral meristem	0	0 / 4229
flower	128	8 / 62048
inflorescence	0	0 / 2246
leaf	328	4 / 12182
root	20	1 / 49512
seed	123	2 / 16148
silique	76	1 / 13014
vegetative tissue	437	1 / 2284

- At.48022 representation biased toward **bud** [more like this]

AT1G30974 p-value = 0.367(flower)

AT1G30972

At.69331

bud	0	0 / 6299
cell culture	0	0 / 1100
floral meristem	0	0 / 4229
flower	16	1 / 62048
inflorescence	0	0 / 2246
leaf	0	0 / 12182
root	0	0 / 49512
seed	0	0 / 16148
silique	0	0 / 13014
vegetative tissue	0	0 / 2284

AT1G31670 p-value = 0.07698(silique)

AT1G31690

At.49932

bud	0	0 / 6299
cell culture	0	0 / 1100
floral meristem	0	0 / 4229
flower	0	0 / 62048
inflorescence	0	0 / 2246
leaf	0	0 / 12182
root	0	0 / 49512
seed	0	0 / 16148
silique	76	1 / 13014
vegetative tissue	0	0 / 2284

AT1G55980 p-value = 0.3052(flower)

At.48311

bud	0	0 / 6299
cell culture	0	0 / 1100
floral meristem	0	0 / 4229
flower	32	2 / 62048
inflorescence	0	0 / 2246
leaf	0	0 / 12182
root	20	1 / 49512
seed	0	0 / 16148
silique	0	0 / 13014
vegetative tissue	0	0 / 2284

AT1G56000 p-value = 0.367(flower)

At.71152

bud	0	0 / 6299
cell culture	0	0 / 1100
floral meristem	0	0 / 4229
flower	16	1 / 62048
inflorescence	0	0 / 2246
leaf	0	0 / 12182
root	0	0 / 49512
seed	0	0 / 16148
silique	0	0 / 13014
vegetative tissue	0	0 / 2284

AT1G62080 p-value = 4.422e-16(seed)

At.28667

bud	0	0 / 6299
cell culture	0	0 / 1100
floral meristem	0	0 / 4229
flower	0	0 / 62048
inflorescence	0	0 / 2246
leaf	0	0 / 12182
root	0	0 / 49512
seed	1114	18 / 16148
silique	230	3 / 13014
vegetative tissue	0	0 / 2284

- At.28667 representation biased toward **seed** [more like this]

AT1G62000 p-value < 2.2e-16(seed)

At.43775

bud	0	0 / 6299
cell culture	0	0 / 1100
floral meristem	0	0 / 4229
flower	0	0 / 62048
inflorescence	0	0 / 2246
leaf	0	0 / 12182
root	0	0 / 49512
seed	1176	19 / 16148
silique	230	3 / 13014
vegetative tissue	0	0 / 2284

- At.43775 representation biased toward **seed** [more like this]

AT1G72590 p-value = 0.2929(root)

At.35081

bud	0	0 / 6299
cell culture	0	0 / 1100
floral meristem	0	0 / 4229
flower	0	0 / 62048
inflorescence	0	0 / 2246
leaf	0	0 / 12182
root	20	1 / 49512
seed	0	0 / 16148
silique	0	0 / 13014
vegetative tissue	0	0 / 2284

AT2G16530 p-value = 0.004063(bud)

At.14490

bud	317	2 / 6299
cell culture	0	0 / 1100
floral meristem	0	0 / 4229
flower	16	1 / 62048
inflorescence	0	0 / 2246
leaf	0	0 / 12182
root	0	0 / 49512
seed	0	0 / 16148
silique	0	0 / 13014
vegetative tissue	0	0 / 2284

AT1G74290 p-value = 0.01329 (inflorescence)

At.34895

bud	0		0 / 6299
cell culture	0		0 / 1100
floral meristem	0		0 / 4229
flower	0		0 / 62048
inflorescence	445		1 / 2246
leaf	0		0 / 12182
root	0		0 / 49512
seed	0		0 / 16148
silique	0		0 / 13014
vegetative tissue	0		0 / 2284

AT1G74280 p-value = 0.022(seed), p-value = 0.03398(root)

At.18150

bud	0		0 / 6299
cell culture	0		0 / 1100
floral meristem	0		0 / 4229
flower	0		0 / 62048
inflorescence	0		0 / 2246
leaf	0		0 / 12182
root	141		7 / 49512
seed	247		4 / 16148
silique	0		0 / 13014
vegetative tissue	437		1 / 2284

AT1G80700 p-value = 0.04944(flower)

At.48404

bud	0		0 / 6299
cell culture	0		0 / 1100
floral meristem	0		0 / 4229
flower	48		3 / 62048
inflorescence	0		0 / 2246
leaf	0		0 / 12182
root	0		0 / 49512
seed	0		0 / 16148
silique	0		0 / 13014
vegetative tissue	0		0 / 2284

AT1G80980

AT2G04390 p-value = 0.009805(cell culture) p-value = 0.0014(flower)

At.63393

bud	0		0 / 6299
cell culture	1818		2 / 1100
floral meristem	0		0 / 4229
flower	257		16 / 62048
inflorescence	0		0 / 2246
leaf	82		1 / 12182
root	60		3 / 49512
seed	0		0 / 16148
silique	76		1 / 13014
vegetative tissue	0		0 / 2284

- At.63393 representation biased toward **cell culture** [more like this]

AT5G04800 p-value = 0.004134(root)

At.48984

bud	158		1 / 6299
cell culture	909		1 / 1100
floral meristem	0		0 / 4229
flower	64		4 / 62048
inflorescence	445		1 / 2246
leaf	82		1 / 12182
root	242		12 / 49512
seed	0		0 / 16148
silique	0		0 / 13014
vegetative tissue	0		0 / 2284

AT2G07715 p-value = 3.984e-08(flower)

ATMG00560

At.70402

bud	0	0 / 6299
cell culture	0	0 / 1100
floral meristem	0	0 / 4229
flower	273	17 / 62048
inflorescence	0	0 / 2246
leaf	0	0 / 12182
root	0	0 / 49512
seed	0	0 / 16148
silique	0	0 / 13014
vegetative tissue	0	0 / 2284

- At.70402 representation biased toward **flower** [more like this]

AT2G07725 p-value = 0.001997(flower)

ATMG00210

At.70267

bud	0	0 / 6299
cell culture	0	0 / 1100
floral meristem	0	0 / 4229
flower	128	8 / 62048
inflorescence	0	0 / 2246
leaf	0	0 / 12182
root	0	0 / 49512
seed	61	1 / 16148
silique	0	0 / 13014
vegetative tissue	0	0 / 2284

AT2G07727 p-value = 5.978e-06(flower)

ATMG00220

At.70126

bud	0	0 / 6299
cell culture	0	0 / 1100
floral meristem	0	0 / 4229
flower	193	12 / 62048
inflorescence	0	0 / 2246
leaf	0	0 / 12182
root	0	0 / 49512
seed	0	0 / 16148
silique	0	0 / 13014
vegetative tissue	0	0 / 2284

- At.70126 representation biased toward **flower** [more like this]

AT2G09990 p-value = 5.483e-08(flower)

At.48467

bud	0	0 / 6299
cell culture	0	0 / 1100
floral meristem	472	2 / 4229
flower	402	25 / 62048
inflorescence	0	0 / 2246
leaf	82	1 / 12182
root	20	1 / 49512
seed	0	0 / 16148
silique	0	0 / 13014
vegetative tissue	0	0 / 2284

AT5G18380 p-value = 1.358e-05(flower)

At.23476

bud	317	2 / 6299
cell culture	1818	2 / 1100
floral meristem	236	1 / 4229
flower	709	44 / 62048
inflorescence	0	0 / 2246
leaf	246	3 / 12182
root	282	14 / 49512
seed	123	2 / 16148
silique	230	3 / 13014
vegetative tissue	0	0 / 2284

AT2G14800 p-value = 0.367(flower)

At.40496

bud	0	0 / 6299
cell culture	0	0 / 1100
floral meristem	0	0 / 4229
flower	16	1 / 62048
inflorescence	0	0 / 2246
leaf	0	0 / 12182
root	0	0 / 49512
seed	0	0 / 16148
silique	0	0 / 13014
vegetative tissue	0	0 / 2284

AT3G44713

AT3G02620 p-value = 0.08577(root)

At.41106

bud	0	0 / 6299
cell culture	0	0 / 1100
floral meristem	0	0 / 4229
flower	0	0 / 62048
inflorescence	0	0 / 2246
leaf	0	0 / 12182
root	40	2 / 49512
seed	0	0 / 16148
silique	0	0 / 13014
vegetative tissue	0	0 / 2284

AT3G02610 p-value = 0.2071(root)

At.41109

bud	0	0 / 6299
cell culture	0	0 / 1100
floral meristem	0	0 / 4229
flower	0	0 / 62048
inflorescence	0	0 / 2246
leaf	0	0 / 12182
root	40	2 / 49512
seed	61	1 / 16148
silique	0	0 / 13014
vegetative tissue	0	0 / 2284

AT3G05160 p-value = 0.009936(root)

At.70075

bud	0	0 / 6299
cell culture	0	0 / 1100
floral meristem	0	0 / 4229
flower	32	2 / 62048
inflorescence	0	0 / 2246
leaf	0	0 / 12182
root	121	6 / 49512
seed	0	0 / 16148
silique	0	0 / 13014
vegetative tissue	0	0 / 2284

AT3G05165 p-value = 0.05173(root)

At.20337

bud	0	0 / 6299
cell culture	0	0 / 1100
floral meristem	0	0 / 4229
flower	64	4 / 62048
inflorescence	0	0 / 2246
leaf	246	3 / 12182
root	181	9 / 49512
seed	0	0 / 16148
silique	153	2 / 13014
vegetative tissue	0	0 / 2284

AT3G23510 p-value = 0.02512 (root)

At.48699

bud	0	0 / 6299
cell culture	0	0 / 1100
floral meristem	0	0 / 4229
flower	0	0 / 62048
inflorescence	0	0 / 2246
leaf	0	0 / 12182
root	60	3 / 49512
seed	0	0 / 16148
silique	0	0 / 13014
vegetative tissue	0	0 / 2284

AT3G23530 p-value = 0.07698(silique)

At.28014

bud	0	0 / 6299
cell culture	0	0 / 1100
floral meristem	0	0 / 4229
flower	0	0 / 62048
inflorescence	0	0 / 2246
leaf	0	0 / 12182
root	0	0 / 49512
seed	0	0 / 16148
silique	76	1 / 13014
vegetative tissue	0	0 / 2284

AT3G28956 p-value = 0.367(flower)

At.48717

bud	0	0 / 6299
cell culture	0	0 / 1100
floral meristem	0	0 / 4229
flower	16	1 / 62048
inflorescence	0	0 / 2246
leaf	0	0 / 12182
root	0	0 / 49512
seed	0	0 / 16148
silique	0	0 / 13014
vegetative tissue	0	0 / 2284

AT5G62950 p-value = 0.2542(root)

At.29025

bud	0	0 / 6299
cell culture	0	0 / 1100
floral meristem	0	0 / 4229
flower	64	4 / 62048
inflorescence	0	0 / 2246
leaf	82	1 / 12182
root	80	4 / 49512
seed	0	0 / 16148
silique	0	0 / 13014
vegetative tissue	0	0 / 2284

AT3G49420 p-value = 0.04944(flower)

At.73030

bud	0	0 / 6299
cell culture	0	0 / 1100
floral meristem	0	0 / 4229
flower	48	3 / 62048
inflorescence	0	0 / 2246
leaf	0	0 / 12182
root	0	0 / 49512
seed	0	0 / 16148
silique	0	0 / 13014
vegetative tissue	0	0 / 2284

AT5G01430 p-value = 0.1433(flower)

At.48968

bud	0	0 / 6299
cell culture	0	0 / 1100
floral meristem	0	0 / 4229
flower	48	3 / 62048
inflorescence	0	0 / 2246
leaf	0	0 / 12182
root	20	1 / 49512
seed	0	0 / 16148
silique	0	0 / 13014
vegetative tissue	0	0 / 2284

AT4G13500 p-value = 0.07206(leaf)

At.33367

bud	0	0 / 6299
cell culture	0	0 / 1100
floral meristem	0	0 / 4229
flower	0	0 / 62048
inflorescence	0	0 / 2246
leaf	82	1 / 12182
root	0	0 / 49512
seed	0	0 / 16148
silique	0	0 / 13014
vegetative tissue	0	0 / 2284

AT2G05310 p-value = 0.01815(flower)

At.41202

bud	0	0 / 6299
cell culture	0	0 / 1100
floral meristem	0	0 / 4229
flower	64	4 / 62048
inflorescence	0	0 / 2246
leaf	0	0 / 12182
root	0	0 / 49512
seed	0	0 / 16148
silique	0	0 / 13014
vegetative tissue	0	0 / 2284

AT4G21460 p-value = 0.003668(vegetative tissue)

At.32629

bud	158	1 / 6299
cell culture	0	0 / 1100
floral meristem	0	0 / 4229
flower	64	4 / 62048
inflorescence	0	0 / 2246
leaf	0	0 / 12182
root	0	0 / 49512
seed	0	0 / 16148
silique	0	0 / 13014
vegetative tissue	875	2 / 2284

AT3G18240 p-value = 0.06776(flower)

At.6712

bud	158	1 / 6299
cell culture	0	0 / 1100
floral meristem	236	1 / 4229
flower	80	5 / 62048
inflorescence	0	0 / 2246
leaf	0	0 / 12182
root	0	0 / 49512
seed	0	0 / 16148
silique	0	0 / 13014
vegetative tissue	0	0 / 2284

AT4G23420 p-value = 0.2929(root)

At.32429

bud	0	0 / 6299
cell culture	0	0 / 1100
floral meristem	0	0 / 4229
flower	0	0 / 62048
inflorescence	0	0 / 2246
leaf	0	0 / 12182
root	20	1 / 49512
seed	0	0 / 16148
silique	0	0 / 13014
vegetative tissue	0	0 / 2284

AT4G23430 p-value = 5.684e-05(root)

At.43783

bud	0	0 / 6299
cell culture	909	1 / 1100
floral meristem	0	0 / 4229
flower	0	0 / 62048
inflorescence	0	0 / 2246
leaf	82	1 / 12182
root	222	11 / 49512
seed	0	0 / 16148
silique	0	0 / 13014
vegetative tissue	0	0 / 2284

- At.43783 representation biased toward **cell culture** [more like this]

AT5G25754 p-value = 0.08026(seed)

At.21471

bud	0	0 / 6299
cell culture	0	0 / 1100
floral meristem	0	0 / 4229
flower	32	2 / 62048
inflorescence	0	0 / 2246
leaf	82	1 / 12182
root	100	5 / 49512
seed	185	3 / 16148
silique	0	0 / 13014
vegetative tissue	0	0 / 2284

AT5G25757 p-value = 4.977e-05(silique)

At.49062

bud	0	0 / 6299
cell culture	0	0 / 1100
floral meristem	0	0 / 4229
flower	0	0 / 62048
inflorescence	0	0 / 2246
leaf	0	0 / 12182
root	40	2 / 49512
seed	0	0 / 16148
silique	384	5 / 13014
vegetative tissue	0	0 / 2284

AT5G28900 p-value = 0.1729(bud)

At.49079

bud	158	1 / 6299
cell culture	0	0 / 1100
floral meristem	0	0 / 4229
flower	32	2 / 62048
inflorescence	0	0 / 2246
leaf	82	1 / 12182
root	0	0 / 49512
seed	61	1 / 16148
silique	0	0 / 13014
vegetative tissue	0	0 / 2284

AT5G28850 p-value = 0.06409(flower)

At.25597

bud	0	0 / 6299
cell culture	0	0 / 1100
floral meristem	0	0 / 4229
flower	64	4 / 62048
inflorescence	0	0 / 2246
leaf	0	0 / 12182
root	0	0 / 49512
seed	61	1 / 16148
silique	0	0 / 13014
vegetative tissue	0	0 / 2284

AT5G36670 p-value = 0.367(flower)

AT5G36740

At.55161

bud	0	0 / 6299
cell culture	0	0 / 1100
floral meristem	0	0 / 4229
flower	16	1 / 62048
inflorescence	0	0 / 2246
leaf	0	0 / 12182
root	0	0 / 49512
seed	0	0 / 16148
silique	0	0 / 13014
vegetative tissue	0	0 / 2284

AT5G36710 p-value = 0.1269(flower)

AT5G36800

At.30536

bud	0	0 / 6299
cell culture	0	0 / 1100
floral meristem	0	0 / 4229
flower	80	5 / 62048
inflorescence	0	0 / 2246
leaf	0	0 / 12182
root	60	3 / 49512
seed	0	0 / 16148
silique	0	0 / 13014
vegetative tissue	0	0 / 2284

AT5G36739 p-value = 0.367(flower)

AT5G36662

At.55165

bud	0	0 / 6299
cell culture	0	0 / 1100
floral meristem	0	0 / 4229
flower	16	1 / 62048
inflorescence	0	0 / 2246
leaf	0	0 / 12182
root	0	0 / 49512
seed	0	0 / 16148
silique	0	0 / 13014
vegetative tissue	0	0 / 2284

AT5G39160 p-value = 0.0004642(silique)

At.49106

bud	0	0 / 6299
cell culture	0	0 / 1100
floral meristem	0	0 / 4229
flower	0	0 / 62048
inflorescence	0	0 / 2246
leaf	0	0 / 12182
root	0	0 / 49512
seed	123	2 / 16148
silique	307	4 / 13014
vegetative tissue	0	0 / 2284

AT5G39190 p-value = 3.706e-09(silique)

At.65

bud	0	0 / 6299
cell culture	0	0 / 1100
floral meristem	0	0 / 4229
flower	0	0 / 62048
inflorescence	0	0 / 2246
leaf	82	1 / 12182
root	20	1 / 49512
seed	557	9 / 16148
silique	998	13 / 13014
vegetative tissue	0	0 / 2284

Parental gene
AT1G21530

AT1G21540 p-value = 0.01329(inflorescence)

At.41650

bud	0	0 / 6299
cell culture	0	0 / 1100
floral meristem	0	0 / 4229
flower	0	0 / 62048
inflorescence	445	1 / 2246
leaf	0	0 / 12182
root	0	0 / 49512
seed	0	0 / 16148
silique	0	0 / 13014
vegetative tissue	0	0 / 2284

AT1G29830

AT1G29820 p-value = 0.06495 (root)

At.40654

bud	0	0 / 6299
cell culture	0	0 / 1100
floral meristem	0	0 / 4229
flower	32	2 / 62048
inflorescence	0	0 / 2246
leaf	0	0 / 12182
root	80	4 / 49512
seed	0	0 / 16148
silique	0	0 / 13014
vegetative tissue	0	0 / 2284

AT1G53890

AT1G53870 p-value = 0.2929 (root)

At.37318

bud	0	0 / 6299
cell culture	0	0 / 1100
floral meristem	0	0 / 4229
flower	0	0 / 62048
inflorescence	0	0 / 2246
leaf	0	0 / 12182
root	20	1 / 49512
seed	0	0 / 16148
silique	0	0 / 13014
vegetative tissue	0	0 / 2284

AT1G68280

AT1G68260 p-value = 0.02817(root)

At.27391

bud	0	0 / 6299
cell culture	0	0 / 1100
floral meristem	0	0 / 4229
flower	0	0 / 62048
inflorescence	0	0 / 2246
leaf	0	0 / 12182
root	80	4 / 49512
seed	0	0 / 16148
silique	76	1 / 13014
vegetative tissue	0	0 / 2284

AT2G09970

AT1G72510 p-value = 0.06495 (root)

At.43372

bud	0	0 / 6299
cell culture	0	0 / 1100
floral meristem	0	0 / 4229
flower	0	0 / 62048
inflorescence	0	0 / 2246
leaf	82	1 / 12182
root	80	4 / 49512
seed	0	0 / 16148
silique	76	1 / 13014
vegetative tissue	0	0 / 2284

AT2G14378

AT4G35165 p-value = 2.194e-06(flower)

At.54610

bud	0	0 / 6299
cell culture	0	0 / 1100
floral meristem	0	0 / 4229
flower	209	13 / 62048
inflorescence	0	0 / 2246
leaf	0	0 / 12182
root	0	0 / 49512
seed	0	0 / 16148
silique	0	0 / 13014
vegetative tissue	0	0 / 2284

- At.54610 representation biased toward **flower** [more like this]

AT2G43440

AT2G43445 p-value = 0.02512 (root)

At.36896

bud	0	0 / 6299
cell culture	0	0 / 1100
floral meristem	0	0 / 4229
flower	0	0 / 62048
inflorescence	0	0 / 2246
leaf	0	0 / 12182
root	60	3 / 49512
seed	0	0 / 16148
silique	0	0 / 13014
vegetative tissue	0	0 / 2284

AT3G17712

AT3G17740 p-value = 0.03934(inflorescence)

At.38665

bud	0	0 / 6299
cell culture	0	0 / 1100
floral meristem	0	0 / 4229
flower	0	0 / 62048
inflorescence	445	1 / 2246
leaf	0	0 / 12182
root	20	1 / 49512
seed	61	1 / 16148
silique	0	0 / 13014
vegetative tissue	0	0 / 2284

AT3G29260

AT3G29250 p-value = 0.0003604 (root)

At.27790

bud	0	0 / 6299
cell culture	0	0 / 1100
floral meristem	0	0 / 4229
flower	0	0 / 62048
inflorescence	0	0 / 2246
leaf	82	1 / 12182
root	161	8 / 49512
seed	0	0 / 16148
silique	0	0 / 13014
vegetative tissue	0	0 / 2284

AT3G45700

AT3G45710 p-value = 0.0006311 (root)

At.35996

bud	0	0 / 6299
cell culture	0	0 / 1100
floral meristem	0	0 / 4229
flower	0	0 / 62048
inflorescence	0	0 / 2246
leaf	0	0 / 12182
root	121	6 / 49512
seed	0	0 / 16148
silique	0	0 / 13014
vegetative tissue	0	0 / 2284

AT4G01180

AT5G59390 p-value = 0.04944(flower)

At.55622

bud	0	0 / 6299
cell culture	0	0 / 1100
floral meristem	0	0 / 4229
flower	48	3 / 62048
inflorescence	0	0 / 2246
leaf	0	0 / 12182
root	0	0 / 49512
seed	0	0 / 16148
silique	0	0 / 13014
vegetative tissue	0	0 / 2284

AT5G06420

AT1G01350 p-value = 0.05533 (root)

At.48142

bud	0	0 / 6299
cell culture	0	0 / 1100
floral meristem	0	0 / 4229
flower	80	5 / 62048
inflorescence	0	0 / 2246
leaf	82	1 / 12182
root	141	7 / 49512
seed	0	0 / 16148
silique	0	0 / 13014
vegetative tissue	0	0 / 2284

AT5G43620

AT1G66500 p-value = 0.0004963 (vegetative tissue)

At.28299

bud	0	0 / 6299
cell culture	0	0 / 1100
floral meristem	0	0 / 4229
flower	16	1 / 62048
inflorescence	0	0 / 2246
leaf	328	4 / 12182
root	80	4 / 49512
seed	0	0 / 16148
silique	0	0 / 13014
vegetative tissue	1313	3 / 2284

- At.28299 representation biased toward **vegetative tissue** [more like this]
