# Supplementary material for: Divergent Evolutionary and Expression Patterns between Lineage Specific New Duplicate Genes and Their Parental Paralogs in Arabidopsis thaliana
Source: PLoS One. 2013 Aug 29;8(8):e72362. doi: 10.1371/journal.pone.0072362 (PMC3756979; doi:10.1371/journal.pone.0072362)
Supplement: Table S1 — 137 lineage-specific duplicated genes. (PDF) [file pone.0072362.s006.pdf]

Table S1 137 lineage-specific duplicated genes

Two genes are lineage_specific_genes	class	new_gene	parental_gene	new_gene_exon	parental_gene_exon
N	tandom	AT1G14185	AT1G14190	q_e:2	h_e:1
N	duplication	AT1G19080	AT3G55490	q_e:8	h_e:7
N	tandom	AT1G21530	AT1G21540	q_e:2	h_e:2
Y	duplication	AT1G24240	AT4G11630	q_e:5	h_e:5
Y	segmental	AT1G24822	AT1G25097	q_e:4	h_e:4
N	segmental	AT1G24880	AT1G25054	q_e:10	h_e:10
N	segmental	AT1G25112	AT1G25025	q_e:1	h_e:1
N	duplication	AT1G29410	AT1G07780	q_e:5	h_e:7
N	duplication	AT1G29620	AT1G32720	q_e:5	h_e:5
N	tandom	AT1G29830	AT1G29820	q_e:9	h_e:8
N	tandom	AT1G30974	AT1G30972	q_e:2	h_e:2
N	tandom	AT1G31670	AT1G31690	q_e:7	h_e:5
N	duplication	AT1G33607	AT5G08055	q_e:2	h_e:2
N	segmental	AT1G34795	AT1G34815	q_e:1	h_e:1
N	tandom/segmental	AT1G34820	AT1G34825	q_e:1	h_e:1
N	segmental	AT1G34830	AT1G34815	q_e:1	h_e:1
N	tandom/segmental	AT1G34850	AT1G34840	q_e:1	h_e:1
N	segmental	AT1G34930	AT1G34825	q_e:1	h_e:1
N	tandom	AT1G43100	AT1G43090	q_e:4	h_e:4
N	duplication	AT1G45190	AT3G11990	q_e:1	h_e:1
Y	tandom	AT1G47690	AT1G47700	q_e:1	h_e:1
Y	duplication	AT1G49680	AT4G28870	q_e:1	h_e:1

N	duplication	AT1G52270	AT4G28310	q_e:1	h_e:1
N	duplication	AT1G53890	AT1G53870	q_e:5	h_e:4
N	duplication	AT1G55980	AT1G56000	q_e:14	h_e:10
N	segmental	AT1G59077	AT1G58766	q_e:5	h_e:5
N	segmental	AT1G59406	AT1G58725	q_e:5	h_e:5
N	duplication	AT1G61200	AT1G20280	q_e:2	h_e:2
N	tandom	AT1G61430	AT1G61440	q_e:8	h_e:7
N	duplication	AT1G62080	AT1G62000	q_e:1	h_e:1
N	duplication	AT1G68280	AT1G68260	q_e:5	h_e:5
N	duplication	AT1G70320	AT1G55860	q_e:15	h_e:18
N	duplication	AT1G72590	AT2G16530	q_e:6	h_e:7
N	duplication	AT1G73607	AT1G49715	q_e:2	h_e:2
N	tandom	AT1G74290	AT1G74280	q_e:3	h_e:2
N	duplication	AT1G80700	AT1G80980	q_e:4	h_e:4
N	duplication	AT2G02840	AT2G06904	q_e:1	h_e:2
N	duplication	AT2G04390	AT5G04800	q_e:2	h_e:2
Y	duplication	AT2G05185	AT5G22608	q_e:3	h_e:3
Y	duplication	AT2G07672	ATMG01050	q_e:1	h_e:1
Y	segmental	AT2G07691	ATMG01310	q_e:1	h_e:1
N	segmental	AT2G07692	ATMG01300	q_e:1	h_e:1
Y	segmental	AT2G07695	ATMG01280	q_e:1	h_e:1
Y	segmental	AT2G07708	ATMG00500	q_e:2	h_e:1
N	segmental	AT2G07713	ATMG00540	q_e:1	h_e:1
N	segmental	AT2G07715	ATMG00560	q_e:3	h_e:2
Y	segmental	AT2G07718	ATMG00590	q_e:1	h_e:1
N	segmental	AT2G07725	ATMG00210	q_e:1	h_e:1
N	segmental	AT2G07727	ATMG00220	q_e:1	h_e:1
Y	segmental	AT2G07734	ATMG00290	q_e:1	h_e:1
N	duplication	AT2G07741	ATMG00410	q_e:1	h_e:1
Y	duplication	AT2G07749	ATMG01110	q_e:1	h_e:1
N	segmental	AT2G07771	ATMG00900	q_e:1	h_e:1
Y	segmental	AT2G07773	ATMG00910	q_e:1	h_e:1
N	duplication	AT2G07776	ATMG00530	q_e:1	h_e:1
N	duplication	AT2G09970	AT1G72510	q_e:1	h_e:3

N	duplication	AT2G09990	AT5G18380	q_e:1	h_e:2
N	duplication	AT2G13450	AT4G02000	q_e:1	h_e:1
N	duplication	AT2G14378	AT4G35165	q_e:1	h_e:1
N	duplication	AT2G14800	AT3G44713	q_e:3	h_e:3
N	duplication	AT2G19850	AT4G04030	q_e:2	h_e:2
N	tandom	AT2G20130	AT2G20120	q_e:7	h_e:7
N	duplication	AT2G31300	AT2G30910	q_e:15	h_e:16
N	tandom	AT2G43440	AT2G43445	q_e:2	h_e:2
N	tandom	AT3G02240	AT3G02242	q_e:3	h_e:3
N	tandom	AT3G02620	AT3G02610	q_e:3	h_e:4
N	tandom	AT3G05160	AT3G05165	q_e:18	h_e:19
N	duplication	AT3G10113	AT1G18330	q_e:5	h_e:6
Y	tandom	AT3G14660	AT3G14650	q_e:5	h_e:5
N	duplication	AT3G17712	AT3G17740	q_e:6	h_e:9
N	tandom	AT3G23510	AT3G23530	q_e:23	h_e:23
N	duplication	AT3G25960	AT3G55650	q_e:1	h_e:1
N	duplication	AT3G27503	AT2G14282	q_e:2	h_e:2
N	duplication	AT3G28300	AT3G28290	q_e:2	h_e:2
Y	duplication	AT3G28674	AT5G04853	q_e:1	h_e:1
N	duplication	AT3G28956	AT5G62950	q_e:5	h_e:5
N	duplication	AT3G29255	AT5G36150	q_e:13	h_e:13
N	duplication	AT3G29260	AT3G29250	q_e:2	h_e:2
N	tandom	AT3G45700	AT3G45710	q_e:4	h_e:4
N	tandom	AT3G47760	AT3G47750	q_e:15	h_e:16
N	duplication	AT3G49420	AT5G01430	q_e:7	h_e:7
N	duplication	AT4G00020	AT5G01630	q_e:22	h_e:20
N	duplication	AT4G01180	AT5G59390	q_e:7	h_e:7
N	duplication	AT4G10860	AT4G10880	q_e:1	h_e:1
N	duplication	AT4G13500	AT2G05310	q_e:3	h_e:3
N	duplication	AT4G14700	AT4G12620	q_e:1	h_e:1
N	tandom	AT4G15230	AT4G15215	q_e:24	h_e:24
N	tandom	AT4G19760	AT4G19750	q_e:3	h_e:2
N	duplication	AT4G21460	AT3G18240	q_e:4	h_e:5

N	tandom	AT4G23420	AT4G23430	q_e:10	h_e:8
N	duplication	AT4G33320	AT4G34080	q_e:1	h_e:1
N	tandom	AT4G34900	AT4G34890	q_e:14	h_e:14
N	duplication	AT4G38320	AT4G37680	q_e:4	h_e:5
N	duplication	AT5G06420	AT1G01350	q_e:4	h_e:4
N	tandom	AT5G25754	AT5G25757	q_e:5	h_e:5
N	duplication	AT5G28900	AT5G28850	q_e:13	h_e:12
Y	duplication	AT5G36310	AT5G36340	q_e:1	h_e:1
Y	tandom/segmental	AT5G36330	AT5G36340	q_e:2	h_e:1
Y	segmental	AT5G36350	AT5G36440	q_e:1	h_e:1
Y	duplication	AT5G36370	AT5G36440	q_e:1	h_e:1
Y	duplication	AT5G36380	AT5G36440	q_e:1	h_e:1
Y	duplication	AT5G36390	AT5G36440	q_e:1	h_e:1
Y	duplication	AT5G36400	AT5G36440	q_e:1	h_e:1
Y	duplication	AT5G36410	AT5G36440	q_e:1	h_e:1
Y	duplication	AT5G36420	AT5G36440	q_e:1	h_e:1
Y	tandom/segmental	AT5G36430	AT5G36440	q_e:1	h_e:1
Y	segmental	AT5G36440	AT5G36460	q_e:1	h_e:1
Y	tandom/segmental	AT5G36450	AT5G36460	q_e:1	h_e:1
Y	tandom/segmental	AT5G36460	AT5G36470	q_e:1	h_e:1
Y	segmental	AT5G36470	AT5G36490	q_e:1	h_e:1
Y	tandom/segmental	AT5G36480	AT5G36490	q_e:1	h_e:1
Y	tandom/segmental	AT5G36490	AT5G36500	q_e:1	h_e:1
Y	tandom/segmental	AT5G36520	AT5G36540	q_e:1	h_e:1
Y	segmental	AT5G36550	AT5G36661	q_e:1	h_e:1

Y	segmental	AT5G36661	AT5G36738	q_e:1	h_e:1
N	segmental	AT5G36670	AT5G36740	q_e:9	h_e:8
N	segmental	AT5G36710	AT5G36800	q_e:1	h_e:1
Y	segmental	AT5G36720	AT5G36805	q_e:1	h_e:1
N	segmental	AT5G36722	AT5G36810	q_e:3	h_e:3
N	duplication	AT5G36738	AT3G42565	q_e:1	h_e:1
N	segmental	AT5G36739	AT5G36662	q_e:1	h_e:1
N	segmental	AT5G36780	AT5G36690	q_e:6	h_e:6
N	duplication	AT5G37270	AT5G37230	q_e:1	h_e:1
N	segmental	AT5G39140	AT5G39200	q_e:2	h_e:2
N	segmental	AT5G39160	AT5G39190	q_e:2	h_e:2
N	duplication	AT5G43620	AT1G66500	q_e:1	h_e:1
N	segmental	AT5G50530	AT5G50640	q_e:15	h_e:15
N	segmental	AT5G50600	AT5G50700	q_e:6	h_e:6
Y	duplication	ATMG00170	ATMG00620	q_e:1	h_e:1
N	duplication	ATMG00200	AT2G07724	q_e:1	h_e:1
Y	duplication	ATMG00430	ATMG01150	q_e:1	h_e:1
N	duplication	ATMG00440	AT2G07702	q_e:1	h_e:1
N	segmental	ATMG00550	AT2G07714	q_e:1	h_e:2
N	segmental	ATMG00620	AT2G07722	q_e:1	h_e:1
N	duplication	ATMG01090	AT2G07777	q_e:1	h_e:1
N	segmental	ATMG01140	AT2G07702	q_e:1	h_e:1
N	segmental	ATMG01150	AT2G07701	q_e:1	h_e:1

new_gene_unigene	parental_gene_unigene
q_unigene:Glucose-methanol-choline_(GMC)_oxidoreductase-like_protein	h_unigene:Glucose-methanol-choline_oxidoreductase-like_protein
q_unigene:	h_unigene:GINS_complex_protein
q_unigene:PMF8-like_amp-binding_protein	h_unigene:Putative_AMP-binding_protein
q_unigene:Ribosomal_protein_L19_family_protein	h_unigene:Ribosomal_protein_L19
q_unigene:Hypothetical_protein	h_unigene:
q_unigene:UDP-3-O-[3-hydroxymyristoyl]_N-acetylglucosamine_deacetylase	h_unigene:
q_unigene:	h_unigene:
q_unigene:	h_unigene:
q_unigene:Cytochrome_C_oxidase_polypeptide_VIB_family_protein	h_unigene:Cytochrome_C_oxidase_polypeptide_VIB_family_protein
q_unigene:Magnesium_transporter_CorA-like_family_protein	h_unigene:Magnesium_transporter_CorA-like_family_protein
q_unigene:Plant_thionin_family_protein	h_unigene:Plant_thionin_family_protein
q_unigene:Copper_amine_oxidase-like_protein	h_unigene:Putative_copper_amine_oxidase
q_unigene:Putative_defensin-like_protein_26	h_unigene:Putative_defensin-like_protein_25
q_unigene:Plant_thionin_family_protein	h_unigene:
q_unigene:	h_unigene:Plant_thionin_family_protein
q_unigene:Plant_thionin_family_protein	h_unigene:
q_unigene:Plant_thionin_family_protein	h_unigene:
q_unigene:Plant_thionin_family_protein	h_unigene:Plant_thionin_family_protein
q_unigene:Glycoside_hydrolase_family_28_protein_/_polygalacturonase_(pectinase)_family_protein	h_unigene:Glycoside_hydrolase_family_28_protein_/_polygalacturonase_(pectinase)_family_protein
q_unigene:	h_unigene:Hypothetical_protein
q_unigene:Hypothetical_protein	h_unigene:Hypothetical_protein
q_unigene:Hypothetical_protein	h_unigene:Hypothetical_protein

q_unigene:Hypothetical_protein	h_unigene:Hypothetical_protein
q_unigene:LURP-one-related_3_protein	h_unigene:LURP-one-related_3_protein
q_unigene:FAD/NAD(P)-binding_oxidoreductase_domain-containing_protein	h_unigene:FAD/NAD(P)-binding_oxidoreductase_domain-containing_protein
q_unigene:	h_unigene:Hypothetical_protein
q_unigene:GDSL_esterase/lipase	h_unigene:
q_unigene:	h_unigene:
q_unigene:Putative_S-locus_protein_kinase	h_unigene:Putative_S-locus_protein_kinase
q_unigene:Hypothetical_protein	h_unigene:Hypothetical_protein
q_unigene:Thioesterase-like_protein	h_unigene:Thioesterase_family_protein
q_unigene:	h_unigene:
q_unigene:3-oxo-5-alpha-steroid_4-dehydrogenase_3	h_unigene:Putative_polyprenol_reductase_2
q_unigene:	h_unigene:Defensin-like_protein_175
q_unigene:Esterase/lipase/thioesterase_family_protein	h_unigene:Alpha/beta-hydrolase_domain-containing_protein
q_unigene:Hypothetical_protein	h_unigene:Hypothetical_protein
q_unigene:Hypothetical_protein	h_unigene:Nucleic_acid_/_zinc_ion_binding_protein
q_unigene:40S_ribosomal_protein_S17-1	h_unigene:40S_ribosomal_protein_S17-4
q_unigene:Hypothetical_protein	h_unigene:Hypothetical_protein
q_unigene:Hypothetical_protein	h_unigene:
q_unigene:Hypothetical_protein	h_unigene:
q_unigene:Hypothetical_protein	h_unigene:
q_unigene:Cytochrome_c_oxidase_subunit_II	h_unigene:
q_unigene:	h_unigene:
q_unigene:	h_unigene:
q_unigene:60S_ribosomal_protein_L2	h_unigene:
q_unigene:Cytochrome_b/b6_protein	h_unigene:
q_unigene:60S_ribosomal_protein_L5	h_unigene:
q_unigene:Cytochrome_b	h_unigene:
q_unigene:Ribosomal_protein_S4	h_unigene:
q_unigene:ATP_synthase_subunit_a-1	h_unigene:
q_unigene:Mitovirus_RNA-dependent_RNA_polymerase	h_unigene:
q_unigene:	h_unigene:
q_unigene:Hypothetical_protein	h_unigene:
q_unigene:Hypothetical_protein	h_unigene:
q_unigene:Hypothetical_protein	h_unigene:Hypothetical_protein

q_unigene:40S_ribosomal_protein_S16-1	h_unigene:40S_ribosomal_protein_S16-3
q_unigene:Hypothetical_protein	h_unigene:Hypothetical_protein
q_unigene:Hypothetical_protein	h_unigene:Hypothetical_protein
q_unigene:Hypothetical_protein	h_unigene:Hypothetical_protein
q_unigene:Hypothetical_protein	h_unigene:
q_unigene:	h_unigene:
q_unigene:	h_unigene:
q_unigene:F-box_protein	h_unigene:F-box/kelch-repeat_protein
q_unigene:	h_unigene:Hypothetical_protein
q_unigene:Acyl-[acyl-carrier-protein]_desaturase	h_unigene:Acyl-[acyl-carrier-protein]_desaturase
q_unigene:Sugar_transporter_ERD6-like_10	h_unigene:Sugar_transporter_ERD6-like_11
q_unigene:Myb_family_transcription_factor	h_unigene:
q_unigene:	h_unigene:
q_unigene:Hypothetical_protein	h_unigene:Hypothetical_protein
q_unigene:Cyclopropane-fatty-acyl-phospholipid_synthase	h_unigene:Cyclopropane-fatty-acyl-phospholipid_synthase
q_unigene:Pyruvate_kinase	h_unigene:Pyruvate_kinase
q_unigene:	h_unigene:
q_unigene:	h_unigene:
q_unigene:Hypothetical_protein	h_unigene:Hypothetical_protein
q_unigene:RNA_polymerase_II,_Rpb4,_core_protein	h_unigene:RNA_polymerase_II,_Rpb4,_core_protein
q_unigene:Putative_pentacyclic_triterpene_synthase_7	h_unigene:
q_unigene:Rossmann-fold_NAD(P)-binding_domain-containing_protein	h_unigene:Rossmann-fold_NAD(P)-binding_domain-containing_protein
q_unigene:Major_facilitator_protein	h_unigene:Major_facilitator_protein
q_unigene:	h_unigene:
q_unigene:Got1/Sft2-like_vescicle_transport-like_protein	h_unigene:Got1/Sft2-like_vescicle_transport_protein
q_unigene:	h_unigene:
q_unigene:XH/XS_domain-containing_protein	h_unigene:XH/XS_domain-containing_protein
q_unigene:Hypothetical_protein	h_unigene:Hypothetical_protein
q_unigene:Hypothetical_protein	h_unigene:Hypothetical_protein
q_unigene:	h_unigene:
q_unigene:	h_unigene:
q_unigene:Glycosyl_hydrolase_family_protein_with_chitinase_insertion_domain	h_unigene:Glycosyl_hydrolase_family_protein_with_chitinase_insertion_domain
q_unigene:Ribosomal_protein_S24/S35	h_unigene:Ribosomal_protein_S24/S35

q_unigene:Rossmann-fold_NAD(P)-binding_domain-containing_protein	h_unigene:Rossmann-fold_NAD(P)-binding_domain-containing_protein
q_unigene:Hypothetical_protein	h_unigene:
q_unigene:	h_unigene:
q_unigene:	h_unigene:
q_unigene:Zinc_finger_CCCH_domain-containing_protein_51	h_unigene:Zinc_finger_CCCH_domain-containing_protein_1
q_unigene:RNA_polymerase_I-associated_factor_PAF67	h_unigene:RNA_polymerase_I-associated_factor_PAF67
q_unigene:Protein_phosphatase_2_(formerly_2A),_regulatory_subunit_B''	h_unigene:Protein_phosphatase_2_(formerly_2A),_regulatory_subunit_B''
q_unigene:ECA1_gametogenesis_related_family_protein	h_unigene:ECA1_gametogenesis_related_family_protein
q_unigene:ECA1_gametogenesis_related_family_protein	h_unigene:ECA1_gametogenesis_related_family_protein
q_unigene:ECA1_gametogenesis_related_family_protein	h_unigene:ECA1_gametogenesis_related_family_protein
q_unigene:ECA1_gametogenesis_related_family_protein	h_unigene:ECA1_gametogenesis_related_family_protein
q_unigene:	h_unigene:ECA1_gametogenesis_related_family_protein
q_unigene:	h_unigene:ECA1_gametogenesis_related_family_protein
q_unigene:	h_unigene:ECA1_gametogenesis_related_family_protein
q_unigene:	h_unigene:ECA1_gametogenesis_related_family_protein
q_unigene:	h_unigene:ECA1_gametogenesis_related_family_protein
q_unigene:	h_unigene:ECA1_gametogenesis_related_family_protein
q_unigene:ECA1_gametogenesis_related_family_protein	h_unigene:ECA1_gametogenesis_related_family_protein
q_unigene:	h_unigene:ECA1_gametogenesis_related_family_protein
q_unigene:ECA1_gametogenesis_related_family_protein	h_unigene:ECA1_gametogenesis_related_family_protein
q_unigene:ECA1_gametogenesis_related_family_protein	h_unigene:ECA1_gametogenesis_related_family_protein
q_unigene:ECA1_gametogenesis_related_family_protein	h_unigene:ECA1_gametogenesis_related_family_protein
q_unigene:ECA1_gametogenesis_related_family_protein	h_unigene:ECA1_gametogenesis_related_family_protein
q_unigene:ECA1_gametogenesis_related_family_protein	h_unigene:ECA1_gametogenesis_related_family_protein
q_unigene:ECA1_gametogenesis_related_family_protein	h_unigene:ECA1_gametogenesis_related_family_protein

q_unigene:ECA1_gametogenesis_related_family_protein	h_unigene:ECA1_gametogenesis_related_family_protein
q_unigene:RING/FYVE/PHD_zinc_finger-containing_protein	h_unigene:Acyl-CoA_N-acyltransferase_with_RING/FYVE/PHD-type_zinc_finger_domain
q_unigene:Hypothetical_protein	h_unigene:Hypothetical_protein
q_unigene:Plant_thionin_family_protein	h_unigene:Plant_thionin_family_protein
q_unigene:Hypothetical_protein	h_unigene:Hypothetical_protein
q_unigene:ECA1_gametogenesis_related_family_protein	h_unigene:ECA1_gametogenesis_related_family_protein
q_unigene:ECA1_gametogenesis_related_family_protein	h_unigene:ECA1_gametogenesis_related_family_protein
q_unigene:	h_unigene:Hypothetical_protein
q_unigene:RING/U-box_domain-containing_protein	h_unigene:RING/U-box_domain-containing_protein
q_unigene:Hypothetical_protein	h_unigene:Hypothetical_protein
q_unigene:Germin-like_protein_subfamily_1_member_18	h_unigene:
q_unigene:Pre-mRNA_cleavage_complex_II	h_unigene:Pre-mRNA_cleavage_complex_II
q_unigene:CBS_/_octicosapeptide/Phox/Bemp1_domain-containing_protein	h_unigene:
q_unigene:	h_unigene:
q_unigene:	h_unigene:
q_unigene:	h_unigene:
q_unigene:	h_unigene:
q_unigene:	h_unigene:Hypothetical_protein
q_unigene:	h_unigene:Transcription_factor-related_protein
q_unigene:	h_unigene:Hypothetical_protein
q_unigene:	h_unigene:ATP_synthase_9
q_unigene:	h_unigene:Hypothetical_protein
q_unigene:	h_unigene:Hypothetical_protein

new_gene_est	parental_gene_est
q_est:wp:1;	h_est:
q_est:leaf:2;	h_est:
q_est:	h_est:Inflorescence:1;wp:1;
q_est:buds:1;wp:2;	h_est:Inflorescence:1;wp:1;
q_est:	h_est:
q_est:Inflorescence:2;buds:2;flower:8;leaf:1; seed:3;siliques:1;wp:2;	h_est:
q_est:	h_est:
q_est:wp:2;	h_est:root:1;wp:2;
q_est:	h_est:
q_est:	h_est:flower:1;root:4;wp:2;
q_est:	h_est:
q_est:siliques:1;	h_est:
q_est:	h_est:
q_est:	h_est:
q_est:	h_est:
q_est:	h_est:
q_est:	h_est:
q_est:	h_est:
q_est:	h_est:
q_est:flower:6;	h_est:flower:7;
q_est:	h_est:
q_est:	h_est:

q_est:	h_est:flower:6;
q_est:	h_est:
q_est:	h_est:wp:2;
q_est:	h_est:
q_est:	h_est:
q_est:	h_est:
q_est:	h_est:
q_est:seed:17;siliques:2;wp:3;	h_est:seed:18;siliques:3;wp:5;
q_est:	h_est:root:4;siliques:1;wp:2;
q_est:Inflorescence:1;flower:8;leaf:1;root:4;seed:4;wp:3;	h_est:flower:9;root:3;seed:6;siliques:3;
q_est:root:1;	h_est:buds:2;wp:2;
q_est:flower:5;	h_est:
q_est:	h_est:Inflorescence:1;flower:3;root:7;seed:2;vegetative:1;
q_est:flower:2;wp:1;	h_est:
q_est:	h_est:
q_est:cellCulture:3;flower:6;leaf:1;root:3;siliques:1;wp:6;	h_est:buds:1;cellCulture:1;flower:6;leaf:1;root:12;wp:6;
q_est:	h_est:
q_est:	h_est:
q_est:	h_est:
q_est:	h_est:
q_est:	h_est:
q_est:flower:7;	h_est:
q_est:	h_est:
q_est:flower:1;	h_est:flower:3;
q_est:	h_est:
q_est:	h_est:
q_est:	h_est:
q_est:	h_est:
q_est:	h_est:
q_est:	h_est:
q_est:	h_est:
q_est:	h_est:
q_est:	h_est:
q_est:	h_est:leaf:1;root:1;siliques:1;wp:2;

q_est:Inflorescence:2;flower:10;inflorescence:2;root:1;	h_est:buds:2;cellCulture:1;flower:32;inflorescence:1;leaf:3;root:14;seed:3;siliques:2;wp:21;
q_est:	h_est:seed:1;
q_est:flower:23;	h_est:flower:13;
q_est:	h_est:
q_est:	h_est:
q_est:flower:1;leaf:1;root:3;siliques:1;wp:2;	h_est:buds:1;flower:2;root:4;seed:1;siliques:1;wp:5;
q_est:siliques:1;wp:1;	h_est:leaf:1;root:6;wp:3;
q_est:	h_est:root:3;
q_est:	h_est:
q_est:root:2;	h_est:root:2;seed:1;
q_est:flower:2;root:6;wp:5;	h_est:flower:3;leaf:4;root:8;siliques:2;wp:5;
q_est:	h_est:siliques:1;vegetative:1;
q_est:flower:2;root:1;seed:3;wp:8;	h_est:root:1;siliques:1;
q_est:wp:1;	h_est:Inflorescence:1;flower:1;root:1;seed:1;
q_est:root:3;wp:1;	h_est:siliques:1;wp:11;
q_est:	h_est:
q_est:	h_est:
q_est:	h_est:
q_est:	h_est:
q_est:wp:2;	h_est:flower:5;leaf:1;root:4;
q_est:	h_est:
q_est:	h_est:leaf:1;root:9;wp:3;
q_est:	h_est:root:6;
q_est:	h_est:flower:2;
q_est:	h_est:root:1;wp:4;
q_est:flower:1;wp:3;	h_est:
q_est:	h_est:flower:3;
q_est:	h_est:
q_est:flower:3;leaf:1;	h_est:flower:6;wp:2;
q_est:leaf:2;	h_est:siliques:1;
q_est:	h_est:
q_est:	h_est:
q_est:flower:2;vegetative:2;wp:2;	h_est:buds:1;inflorescence:1;wp:2;

q_est:root:1;	h_est:cellCulture:1;leaf:1;root:10;wp:1;
q_est:	h_est:
q_est:	h_est:flower:1;leaf:1;root:4;seed:2;siliques:1;wp:3;
q_est:root:16;wp:1;	h_est:leaf:1;root:1;wp:5;
q_est:wp:1;	h_est:flower:6;leaf:1;root:6;
q_est:root:2;wp:1;	h_est:root:1;wp:1;
q_est:buds:1;flower:1;leaf:1;seed:1;wp:4;	h_est:flower:5;
q_est:	h_est:
q_est:	h_est:
q_est:	h_est:
q_est:	h_est:
q_est:	h_est:
q_est:	h_est:
q_est:	h_est:
q_est:	h_est:
q_est:	h_est:
q_est:	h_est:
q_est:	h_est:flower:2;
q_est:	h_est:flower:2;
q_est:flower:2;	h_est:
q_est:	h_est:
q_est:	h_est:
q_est:	h_est:
q_est:	h_est:
q_est:	h_est:

q_est:	h_est:
q_est:	h_est:
q_est:	h_est:
q_est:	h_est:
q_est:	h_est:
q_est:	h_est:
q_est:	h_est:
q_est:	h_est:
q_est:	h_est:
q_est:	h_est:
q_est:	h_est:
q_est:	h_est:flower:1;leaf:2;root:4;vegetative:3; wp:2;
q_est:	h_est:
q_est:	h_est:
q_est:	h_est:
q_est:	h_est:
q_est:	h_est:
q_est:	h_est:
q_est:	h_est:
q_est:	h_est:
q_est:	h_est:
q_est:	h_est:
q_est:	h_est:

ka/ks	ks	ka	P(ka/ks=0.5)	P(ka/ks=1)
0.1562	0.2459	0.0384	7.81E-09	4.77E-20
0.001	0.0112	0	0.09055655	0.03360179
0.1041	0.5495	0.0572	1.04E-25	6.02E-52
0.1732	0.1631	0.0282	0.003238752	1.56E-06
0.2127	0	0	0.9977432	0.9970146
0.4918	0	0	1	0.9977432
0.51	0	0	1	0.9980456
0.4284	0.3294	0.1411	0.4880086	0.000240484
3.1295	0.0092	0.0287	0.0392282	0.2288025
0.2536	0.3116	0.079	3.04E-05	1.54E-16
0.7628	0.116	0.0885	0.4391869	0.6419707
0.1749	0.5588	0.0978	7.29E-17	5.78E-43
0.5447	0.1858	0.1012	0.8551178	0.2088711
0.001	0.0234	0	0.1064914	0.05440555
0.5105	0	0	1	0.9984042
0.3934	0	0	0.9988716	0.9984042
0.4158	0	0	0.9988716	0.9984042
1.8425	0.016	0.0295	0.1791193	0.5554419
2.2083	0.0025	0.0055	0.1239684	0.4375094
0.4519	0.0902	0.0408	0.8087033	0.06335603
0.4712	0	0	1	0.9988716
0.7894	0.0119	0.0094	0.7219837	0.8572878

0.4907	0.2789	0.1369	0.9526225	0.02865835
0.4635	0	0	0.9988716	0.9980456
0.001	0	0	0.9938197	0.9933245
0.4301	0	0	0.9972361	0.9959316
0.001	0	0	0.9937175	0.9934205
0.1926	0.0994	0.0191	0.2140845	0.03184689
0.293	0.2533	0.0742	0.00012656	7.66E-18
1.7829	0.0337	0.06	0.01215365	0.2789066
0.3211	0.2943	0.0945	0.09216939	2.17E-05
0.2324	0.1272	0.0296	3.72E-17	3.07E-56
0.2455	0.2371	0.0582	0.001736686	1.05E-09
0.4008	0.0278	0.0112	0.8272184	0.3738922
0.2927	0.1877	0.0549	0.02211656	2.83E-07
0.0668	0.0286	0.0019	0.05548155	0.01029762
0.427	0.3525	0.1505	0.5548454	0.001936005
0.0818	0.1203	0.0098	0.002045029	1.40E-05
0.2688	0	0	0.9988716	0.9988716
99	0	0.0033	0.2184157	0.3442872
0.4107	0	0	1	0.9984042
0.4686	0	0	1	0.9988716
0.2414	0.0059	0.0014	0.610713	0.3338303
0.4098	0	0	1	0.9984042
0.4324	0	0	1	1
0.5997	0.1302	0.0781	0.4810208	0.05965767
0.2512	0	0	0.9984042	0.9977432
0.4073	0	0	0.9988716	0.9977432
0.001	0.0046	0	0.126558	0.06584944
0.4835	0	0	1	0.9988716
0.001	0	0	0.9950815	0.9945885
0.4436	0.009	0.004	0.9065659	0.4304185
0.4832	0	0	1	0.9980456
0.8353	0.0422	0.0352	0.2896116	0.7239706
0.4269	0	0	1	1
0.2591	0.2339	0.0606	0.04906377	8.25E-05

0.0336	0.0858	0.0029	0.001415518	4.11E-05
0.2653	0.0819	0.0217	0.09828563	0.000777269
0.3042	0.065	0.0198	0.4216794	0.05703797
0.6753	0.1998	0.1349	0.1109542	0.04684464
99	0	0.0039	0.2412937	0.371222
0.3382	0.0723	0.0245	0.3432498	0.01006406
0.1725	0.0757	0.0131	0.005175085	4.41E-06
0.3422	0.2313	0.0792	0.06310416	3.36E-07
1.0375	0.2538	0.2633	0.05218257	0.9250669
0.2209	0.1495	0.033	0.001303232	3.92E-09
0.4098	0.2695	0.1104	0.2488732	5.06E-07
0.3038	0.0186	0.0057	0.5194587	0.1360541
0.2658	0.1732	0.046	0.001821754	1.42E-10
0.4072	0.0593	0.0242	0.4344273	0.000987879
0.1652	0.0838	0.0138	5.13E-06	1.61E-13
0.1842	0.2058	0.0379	8.39E-07	9.98E-17
0.7661	0.308	0.2359	0.3030082	0.5530486
0.001	0	0	0.9949538	0.9947075
0.6127	0.0915	0.0561	0.746924	0.4422699
0.1913	0.1406	0.0269	0.05258251	0.001237114
0.2285	0.4182	0.0955	3.45E-09	1.04E-27
0.184	0.384	0.0706	8.05E-06	5.94E-14
0.3302	0.2685	0.0887	0.008546771	4.13E-12
0.1841	0.3887	0.0715	1.01E-15	3.48E-41
0.001	0.0412	0	0.008716361	0.001672401
0.6191	0.0688	0.0426	0.2298845	0.009611662
0.4423	0.139	0.0615	0.5949722	0.000644331
1.2152	0.1114	0.1353	0.03290179	0.6687957
0.1518	0.2221	0.0337	0.003620894	4.34E-06
0.1537	0.3136	0.0482	5.44E-14	2.11E-31
0.2825	0.2155	0.0609	3.62E-07	2.11E-28
0.3709	0.1955	0.0725	0.1839897	1.73E-05
0.1651	0.148	0.0244	0.000130415	8.13E-10

0.2472	0.2074	0.0513	0.003963246	1.99E-08
1.2619	0.0243	0.0307	0.08428271	0.6814251
0.1486	0.2293	0.0341	1.84E-22	1.20E-52
0.8762	0.01	0.0087	0.4162375	0.8535707
0.1984	0.1333	0.0265	0.004702561	1.01E-06
0.001	0.0098	0	0.009516427	0.001069233
0.0203	0.076	0.0015	2.11E-09	5.50E-14
0.4518	0	0	1	0.9984042
99	0.0001	0.0126	0.059934	0.1547348
0.4562	0	0	1	0.9984042
99	0	0.004	0.2846357	0.4197111
0.4702	0	0	1	0.9984042
0.504	0	0	1	0.9984042
0.3901	0	0	0.9988716	0.9980456
0.4284	0	0	1	0.9984042
0.001	0	0	0.9970146	0.9966149
0.3738	0	0	0.9988716	0.9980456
0.001	0.0103	0	0.200671	0.1120454
0.001	0.0103	0	0.200671	0.1120454
0.001	0.0103	0	0.200671	0.1120454
99	0	0.0036	0.3608307	0.4981366
99	0.0001	0.0072	0.1985834	0.3406642
99	0	0.004	0.2827684	0.4176856
0.7628	0.0106	0.0081	0.7265167	0.8287029
0.3402	0.0709	0.0241	0.5168123	0.07439243

0.4459	0	0	1	0.9984042
0.5329	0.0173	0.0092	0.8836082	0.1794337
0.001	0	0	0.9964318	0.9960912
0.4488	0	0	1	0.9988716
0.4822	0	0	1	0.9988716
0.3527	0.1083	0.0382	0.4786253	0.03992893
0.4048	0	0	1	0.9984042
0.4488	0	0	0.9988716	0.9988716
0.387	0.0757	0.0293	0.5918851	0.05588102
0.001	0.007	0	0.2254881	0.1288096
99	0	0.0023	0.2319045	0.3603744
0.3014	0.1204	0.0363	0.06266982	1.72E-05
0.3998	0	0	0.9974769	0.9962576
0.3671	0	0	0.9988716	0.9977432
0.3654	0	0	0.9988716	0.9980456
99	0	0.0047	0.2383598	0.3678088
0.4649	0	0	1	0.9984042
0.4809	0	0	1	0.9984042
0.6165	0.0779	0.048	0.6502683	0.316005
99	0	0.0038	0.2279925	0.3556859
99	0	0.0032	0.1973597	0.3393816
0.4249	0	0	1	0.9984042
0.4877	0	0	0.9988716	0.9984042
