# Supplementary material for: Divergent Evolutionary and Expression Patterns between Lineage Specific New Duplicate Genes and Their Parental Paralogs in Arabidopsis thaliana
Source: PLoS One. 2013 Aug 29;8(8):e72362. doi: 10.1371/journal.pone.0072362 (PMC3756979; doi:10.1371/journal.pone.0072362)
Supplement: Table S2 — Lineage-specific chimeric duplicated genes. (PDF) [file pone.0072362.s007.pdf]

Table S2 Lineage-specific chimeric duplicated genes

7 chimera genes from multiple parental gene

new gene	parental gene 1	parental gene 2	parental gene 3
AT1G20290	AT1G20370	AT1G20240	no
AT1G55980	AT4G33620	AT1G56000	no
AT2G07698	ATMG01200	ATMG01190	no
AT3G03350	AT3G03330	AT2G47220	no
AT3G30200	AT1G07110	AT1G40087	no
AT3G62500	AT3G61740	AT3G61730	no
ATMG00040	ATMG01090	ATMG01080	ATMG01020

4 chimera genes from parental gene and Tranposable element

new gene	parental gene 1	TE
AT1G34095	AT2G45403	AT1TE40360
AT2G26120	AT2G31590	SSR102702
AT4G19270	AT3G50250	SSR205636
AT5G48205	AT2G41835	SSR271695
