# Supplementary material for: Divergent Evolutionary and Expression Patterns between Lineage Specific New Duplicate Genes and Their Parental Paralogs in Arabidopsis thaliana
Source: PLoS One. 2013 Aug 29;8(8):e72362. doi: 10.1371/journal.pone.0072362 (PMC3756979; doi:10.1371/journal.pone.0072362)
Supplement: Table S3 — 92 NDG branch specific Ka/Ks and background Ka/Ks. (PDF) [file pone.0072362.s008.pdf]

Table S3 92 NDG branch specific Ka/Ks and background Ka/Ks

paralog pairs	NDG branch specific	background Ka/Ks	LRT P value
AT1G14185_AT1G14190	0.32714	0.12994	4.83E-05
AT1G19080_AT3G55490	0.0001	0.08242	0.08745772
AT1G21530_AT1G21540	0.10831	0.06676	8.09E-40
AT1G24880_AT1G25054	0.23673	0.28191	0.9970146
AT1G29410_AT1G07780	0.62481	0.19684	0.06877022
AT1G29620_AT1G32720	999	0.1673	0.4019108
AT1G29830_AT1G29820	0.41067	0.11529	2.74E-06
AT1G31670_AT1G31690	0.22345	0.18314	1.76E-31
AT1G33607_AT5G08055	0.80394	0.43896	0.7452731
AT1G34795_AT1G34815	8.1605	0.64908	0.9960912
AT1G34820_AT1G34825	8.34638	0.63079	0.9966149
AT1G34830_AT1G34815	3.64115	0.64652	0.9970146
AT1G34850_AT1G34840	7.44734	0.64881	0.997236
AT1G34930_AT1G34825	999	0.53095	0.214304
AT1G43100_AT1G43090	1.41648	0.19235	0.758374
AT1G45190_AT3G11990	0.20489	0.35256	0.01915317
AT1G53890_AT1G53870	33.35325	0.47187	0.9970146
AT1G55980_AT1G56000	0.0001	0.15222	0.9898449
AT1G59077_AT1G58766	0.0001	0.36442	0.9901633
AT1G59406_AT1G58725	79.97275	0.1086	0.9944721
AT1G61200_AT1G20280	0.56182	0.20384	0.7561468
AT1G61430_AT1G61440	0.39035	0.27305	2.64E-07
AT1G62080_AT1G62000	0.87081	0.96122	0.8676318
AT1G68280_AT1G68260	0.42217	0.17332	0.01502618
AT1G70320_AT1G55860	0.29232	0.21747	8.79E-25
AT1G72590_AT2G16530	0.29542	0.23895	5.14E-06
AT1G73607_AT1G49715	0.0001	0.72634	0.9988716
AT1G74290_AT1G74280	0.1768	0.27649	2.67E-07
AT1G80700_AT1G80980	0.13452	0.24903	0.0952239
AT2G02840_AT2G06904	999	0.07517	0.2246263
AT2G04390_AT5G04800	0.2651	0.05058	0.1999516
AT2G07713_ATMG00540	26.5938	2.21592	0.9988716
AT2G07715_ATMG00560	0.69389	0.68873	0.1865295
AT2G07727_ATMG00220	0.0001	0.00997	0.7837203
AT2G07741_ATMG00410	4.81232	0.59756	0.9932298
AT2G07771_ATMG00900	0.0001	999	0.993617
AT2G07776_ATMG00530	999	999	0.03219326
AT2G09970_AT1G72510	0.41743	0.11836	0.04435506
AT2G09990_AT5G18380	0.04365	0.13028	0.000692969
AT2G13450_AT4G02000	0.72179	0.08126	0.9474382
AT2G14378_AT4G35165	0.32563	0.1578	0.2044997
AT2G14800_AT3G44713	2.1539	0.31554	0.3550337
AT2G19850_AT4G04030	999	0.56395	0.4053384
AT2G20130_AT2G20120	0.58326	0.06903	0.3393948
AT2G31300_AT2G30910	0.26456	0.07722	0.011968
AT2G43440_AT2G43445	0.32544	0.42548	6.86E-05
AT3G02240_AT3G02242	0.56353	1.39927	0.1487881

AT3G02620_AT3G02610	0.37162	0.14033	0.002290511
AT3G05160_AT3G05165	0.17989	0.36761	5.47E-13
AT3G10113_AT1G18330	0.51332	0.29264	0.4654949
AT3G14660_AT3G14650	0.23398	0.16347	3.19E-07
AT3G17712_AT3G17740	0.57107	0.23112	0.03658901
AT3G23510_AT3G23530	0.26049	0.09946	9.37E-05
AT3G25960_AT3G55650	0.13938	0.1391	7.71E-12
AT3G27503_AT2G14282	1.21341	0.38396	0.8484456
AT3G28300_AT3G28290	121.91735	0.08647	0.9970146
AT3G28956_AT5G62950	0.32635	0.24013	0.2725902
AT3G29255_AT5G36150	0.29593	0.1908	5.28E-07
AT3G29260_AT3G29250	0.11832	0.15264	2.64E-09
AT3G45700_AT3G45710	0.35455	0.29651	5.97E-07
AT3G47760_AT3G47750	0.19104	0.24264	1.53E-28
AT3G49420_AT5G01430	0.0001	0.03329	0.000524946
AT4G00020_AT5G01630	0.74971	0.33275	0.2170339
AT4G01180_AT5G59390	0.6707	0.25741	0.1555245
AT4G10860_AT4G10880	3.10958	0.36162	0.2786599
AT4G13500_AT2G05310	0.07545	0.18091	1.28E-06
AT4G14700_AT4G12620	0.16129	0.11155	2.90E-20
AT4G15230_AT4G15215	0.34204	0.2134	2.52E-14
AT4G19760_AT4G19750	0.38001	0.42784	0.003391784
AT4G21460_AT3G18240	0.22805	0.14624	8.41E-05
AT4G23420_AT4G23430	0.25356	0.15881	5.43E-06
AT4G33320_AT4G34080	1.26918	0.1006	0.6799466
AT4G34900_AT4G34890	0.18341	0.09858	1.07E-31
AT4G38320_AT4G37680	0.91424	0.03689	0.9261474
AT5G06420_AT1G01350	0.34507	0.08026	0.001008709
AT5G25754_AT5G25757	0.0001	0.07799	0.005455417
AT5G28900_AT5G28850	0.02232	0.07569	1.83E-07
AT5G36670_AT5G36740	0.73216	0.43402	0.4931427
AT5G36710_AT5G36800	28.83444	0.11127	0.995778
AT5G36722_AT5G36810	1.16276	0.40517	1
AT5G36738_AT3G42565	0.29096	0.25546	0.1141949
AT5G36739_AT5G36662	14.46475	0.20702	0.9960912
AT5G36780_AT5G36690	0.0001	0.37672	0.9897824
AT5G37270_AT5G37230	0.3021	0.42669	0.07925312
AT5G39140_AT5G39200	0.0001	0.28805	0.09308956
AT5G39160_AT5G39190	999	0.1435	0.4250909
AT5G43620_AT1G66500	0.46189	0.26107	0.08193959
AT5G50530_AT5G50640	0.0001	0.0726	0.9864132
AT5G50600_AT5G50700	0.0001	0.12481	0.1029145
ATMG00550_AT2G07714	0.68343	0.16973	0.4240304
AT1G30974_AT1G30972	1.78679	0.4473	0.6017824
AT1G52270_AT4G28310	0.58931	0.24407	0.1976082
