# Supplementary material for: Divergent Evolutionary and Expression Patterns between Lineage Specific New Duplicate Genes and Their Parental Paralogs in Arabidopsis thaliana
Source: PLoS One. 2013 Aug 29;8(8):e72362. doi: 10.1371/journal.pone.0072362 (PMC3756979; doi:10.1371/journal.pone.0072362)
Supplement: Table S4 — Comparison of the Ka, Ks, and Ka/Ks values between NDGs and 10 simulated duplicated gene datasets. (PDF) [file pone.0072362.s009.pdf]

Table S4 Comparison of the Ka, Ks, and Ka/Ks values between NDGs and 10 simulated duplicated gene datasets

	average Ks	average Ka	Ks,Ka=0	Ka/Ks > 1	Ka/Ks < 1	Ka/Ks < 0.5*	Ka/Ks < 1*
sample 1	1.08739	0.134019	1	1	85	66	79
sample 2	0.952406	0.136602	1	3	92	77	89
sample 3	0.984306	0.134788	2	2	87	71	83
sample 4	1.04995	0.13196	3	3	82	71	77
sample 5	0.899707	0.140662	1	0	92	76	82
sample 6	1.05846	0.144813	0	4	83	68	80
sample 7	1.02956	0.131068	2	1	93	72	85
sample 8	0.96318	0.141251	1	1	81	66	80
sample 9	0.939748	0.139921	1	2	90	66	81
sample 10	0.928966	0.143247	1	2	89	74	86
NDGs and parental genes	0.112318	0.037401	25	12	89	30	49
