# Supplementary material for: Divergent Evolutionary and Expression Patterns between Lineage Specific New Duplicate Genes and Their Parental Paralogs in Arabidopsis thaliana
Source: PLoS One. 2013 Aug 29;8(8):e72362. doi: 10.1371/journal.pone.0072362 (PMC3756979; doi:10.1371/journal.pone.0072362)
Supplement: Table S5 — The population genetics statistics of 10 datasets of 100 duplicated genes. (PDF) [file pone.0072362.s010.pdf]

Table S5 The population genetics statistics of 10 datasets of 100 duplicated genes

Average value	π_a	π_s	π_n	θ_a	θ_s	θ_n
New gene	0.0054	0.0069	0.0039	0.0082	0.0101	0.0063
Parental gene	0.0049	0.0071	0.0029	0.0071	0.0094	0.0047
sample 1	0.00449173	0.00594974	0.00228627	0.00686558	0.0087851	0.00411626
sample 2	0.00515072	0.00739725	0.00273935	0.00753609	0.0101769	0.0046137
sample 3	0.00490263	0.0069705	0.00250598	0.00647769	0.00875752	0.00359099
sample 4	0.00495274	0.00704691	0.00264337	0.00705613	0.0093396	0.00432585
sample 5	0.00457513	0.00632639	0.0026556	0.00643609	0.00838442	0.00422199
sample 6	0.00581255	0.00835327	0.00307861	0.00793308	0.0108007	0.00489298
sample 7	0.00518072	0.00716777	0.0028966	0.00672731	0.00883408	0.00400616
sample 8	0.00482176	0.00704361	0.00293806	0.0073318	0.00992737	0.00488768
sample 9	0.00444942	0.00622852	0.0023335	0.00690004	0.00915023	0.00426901
sample 10	0.00508905	0.00706294	0.00281498	0.00738926	0.00977141	0.00459003
