# Supplementary material for: Divergent Evolutionary and Expression Patterns between Lineage Specific New Duplicate Genes and Their Parental Paralogs in Arabidopsis thaliana
Source: PLoS One. 2013 Aug 29;8(8):e72362. doi: 10.1371/journal.pone.0072362 (PMC3756979; doi:10.1371/journal.pone.0072362)
Supplement: Table S6 — Ten new genes with selection signature. (PDF) [file pone.0072362.s011.pdf]

Table S6 Ten new genes with selection signature

new_gene	pi	syn_pi	nonsyn_pi	theta	syn_theta	nonsyn_theta	td	q_td	fl_f	q_fl_f	fl_d	p_fl_d
AT4G01180	0.002986063	0.003986334	0.00242725	0.009481959	0.014191817	0.006854125	-2.30607613	0.04946487*	-3.71019248	0.04758338*	-3.63307448	0.05372182
AT4G14700	0.003036724	0.003937716	0.002745315	0.009336817	0.01507685	0.007485539	-2.28364439	0.04946487*	-3.8443781	0.04758338*	-3.84305532	0.05372182
AT1G29410	0.002247296	0.002353841	0.001946034	0.007017615	0.006566858	0.008262758	-2.26163432	0.04946487*	-4.57968562	0.04758338*	-4.86669801	0.04722797*
AT1G61430	0.002798901	0.003488551	0.002138087	0.007546592	0.008389142	0.006737085	-2.13892989	0.05077916	-4.20537159	0.04758338*	-4.46153216	0.04722797*
AT4G15230	0.001551573	0.002072224	0.001001041	0.004309824	0.005337821	0.003222447	-2.17785025	0.05077916	-4.06283908	0.04758338*	-4.23358124	0.04722797*
AT2G02840	0.013562041	0.012129683	0.017235533	0.034010498	0.035112815	0.031061336	-2.06391977	0.05971629	-3.82356239	0.04758338*	-3.98772532	0.05009773
AT3G29255	0.008393907	0.009276123	0.00699859	0.012560302	0.013276784	0.01142369	-1.14209807	0.0828926	-1.83260145	0.08612592	-1.80356278	0.08729796
AT1G21530	0.003880392	0.007412014	0.002431817	0.003843536	0.005446207	0.003184522	0.030316039	0.05925402	0.003777311	0.0528022	-0.0137296	0.09173043
AT3G25960	0.005644519	0.008254295	0.004850759	0.00864894	0.013382334	0.007210667	-1.14758462	0.0828926	-1.99012828	0.08612592	-2.0004557	0.08729796
AT1G31670	0.012489262	0.017353477	0.005177303	0.014168329	0.017698155	0.008859296	-0.40866397	0.08540648	-0.45295145	0.08862859	-0.35466691	0.08879271

q_MK_test
1
0.04247955*
0.8349005
0.01781932*
0.1203755
0.923286
0.008356982*
0.008356982*
0.0008517093*
0.02333683*
