# Supplementary material for: Divergent Evolutionary and Expression Patterns between Lineage Specific New Duplicate Genes and Their Parental Paralogs in Arabidopsis thaliana
Source: PLoS One. 2013 Aug 29;8(8):e72362. doi: 10.1371/journal.pone.0072362 (PMC3756979; doi:10.1371/journal.pone.0072362)
Supplement: Table S7 — Tiling array data of 62 new genes. (PDF) [file pone.0072362.s012.pdf]

Table S7 Tiling array data of 62 new genes

| new_gene | roots | seedlings | expanding_leaves | senescing_leaves | stem |
|---|---|---|---|---|---|
| AT1G14185 | 8.553966667 | 6.233233333 | 5.785053333 | 5.672416667 | 6.506983333 |
| AT1G19080 | 7.909793333 | 8.153746667 | 8.125816667 | 8.02381 | 8.164693333 |
| AT1G21530 | 7.371356667 | 6.40036 | 6.241876667 | 6.312413333 | 6.165766667 |
| AT1G29410 | 9.106366667 | 9.487803333 | 9.464066667 | 8.809966667 | 8.99678 |
| AT1G29830 | 6.44521 | 5.834813333 | 5.737933333 | 5.6991 | 5.735593333 |
| AT1G30974 | 5.342196667 | 5.541056667 | 5.638396667 | 6.12602 | 5.741876667 |
| AT1G31670 | 5.88975 | 5.947476667 | 6.146226667 | 5.721623333 | 5.738986667 |
| AT1G33607 | 5.18571 | 5.48151 | 4.82028 | 5.29995 | 5.1189 |
| AT1G34930 | 3.85445 | 4.350216667 | 5.121256667 | 3.8486 | 4.19688 |
| AT1G43100 | 7.04551 | 7.430156667 | 7.369593333 | 7.52279 | 7.279626667 |
| AT1G45190 | 5.18823 | 4.851123333 | 4.78442 | 4.837303333 | 4.896043333 |
| AT1G52270 | 7.023516667 | 8.421496667 | 8.240156667 | 7.108686667 | 9.19958 |
| AT1G55980 | 5.573843333 | 5.575823333 | 5.376496667 | 5.75336 | 5.4317 |
| AT1G61200 | 9.330706667 | 8.72818 | 8.45237 | 9.640213333 | 8.288846667 |
| AT1G61430 | 6.932383333 | 7.099643333 | 7.420583333 | 7.426823333 | 6.79913 |
| AT1G62080 | 6.27956 | 6.202523333 | 6.139666667 | 6.38511 | 5.966236667 |
| AT1G68280 | 6.274136667 | 6.571226667 | 6.86288 | 6.0481 | 6.867046667 |
| AT1G70320 | 6.38757 | 6.290233333 | 6.34432 | 6.550293333 | 6.360866667 |
| AT1G72590 | 6.782366667 | 6.12647 | 6.034646667 | 6.272726667 | 6.48083 |
| AT1G74290 | 6.34862 | 6.168006667 | 6.296786667 | 6.985703333 | 5.78432 |
| AT1G80700 | 10.32293333 | 10.6125 | 10.50663333 | 10.26301 | 10.52063333 |
| AT2G02840 | 5.861523333 | 5.82221 | 6.116666667 | 6.549286667 | 6.193216667 |
| AT2G04390 | 9.672753333 | 10.05098333 | 10.11259333 | 9.149786667 | 9.622743333 |
| AT2G09970 | 7.56254 | 7.09348 | 7.52142 | 7.69132 | 6.511313333 |
| AT2G09990 | 11.40606667 | 11.5054 | 11.27446667 | 10.38781333 | 11.23136667 |
| AT2G13450 | 4.713903333 | 4.818386667 | 4.996696667 | 5.38484 | 4.650456667 |
| AT2G14378 | 4.734053333 | 5.245693333 | 4.741486667 | 4.67693 | 5.347783333 |
| AT2G14800 | 5.86436 | 5.99262 | 5.689733333 | 5.844553333 | 5.720346667 |
| AT2G19850 | 5.6021 | 5.637803333 | 5.842136667 | 5.927296667 | 6.262083333 |
| AT2G20130 | 9.92921 | 9.444313333 | 9.461996667 | 9.484583333 | 9.404906667 |
| AT2G31300 | 10.29036667 | 9.687113333 | 9.87696 | 9.582413333 | 10.28033333 |
| AT2G43440 | 6.552166667 | 7.27297 | 7.108696667 | 7.460006667 | 6.756186667 |
| AT3G02240 | 7.20432 | 3.915093333 | 4.100216667 | 4.045946667 | 3.90154 |
| AT3G02620 | 10.23335667 | 7.168696667 | 7.168833333 | 7.42691 | 6.937926667 |
| AT3G05160 | 8.944916667 | 10.21186667 | 10.5495 | 10.94253333 | 8.051506667 |
| AT3G10113 | 3.939516667 | 4.18467 | 4.958433333 | 4.561723333 | 4.653376667 |
| AT3G23510 | 7.92571 | 6.525186667 | 6.945443333 | 6.339506667 | 6.32872 |
| AT3G25960 | 5.392423333 | 5.557366667 | 5.814496667 | 5.93062 | 5.513613333 |
| AT3G27503 | 4.435396667 | 4.592073333 | 4.375273333 | 4.52759 | 4.50874 |
| AT3G28956 | 9.58523 | 9.390306667 | 9.405573333 | 8.910123333 | 9.551883333 |
| AT3G29255 | 5.061056667 | 5.21782 | 5.122263333 | 5.435356667 | 5.261136667 |
| AT3G29260 | 8.078543333 | 6.539133333 | 6.685216667 | 7.352113333 | 6.51586 |
| AT3G45700 | 9.062146667 | 5.852686667 | 5.414593333 | 5.62262 | 5.305693333 |
| AT3G47760 | 6.339816667 | 6.274103333 | 5.93193 | 6.45734 | 6.113746667 |
| AT3G49420 | 7.1784 | 6.92008 | 6.601603333 | 6.379516667 | 6.4012 |

| | | | | | |
|---|---|---|---|---|---|
| AT4G00020 | 6.159853333 | 6.39919 | 6.274283333 | 5.812453333 | 6.26825 |
| AT4G01180 | 6.55135 | 6.364453333 | 6.604263333 | 6.59954 | 6.51953 |
| AT4G10860 | 5.101356667 | 4.52207 | 6.344496667 | 7.636323333 | 4.683406667 |
| AT4G13500 | 7.261906667 | 9.914783333 | 10.46093333 | 9.77245 | 9.284863333 |
| AT4G14700 | 6.453663333 | 6.242186667 | 5.71221 | 5.527563333 | 6.72165 |
| AT4G15230 | 6.225703333 | 5.881346667 | 5.740056667 | 5.639263333 | 5.685833333 |
| AT4G19760 | 5.846173333 | 5.84389 | 5.743743333 | 6.161353333 | 5.51609 |
| AT4G21460 | 8.14149 | 8.26743 | 7.931383333 | 7.334553333 | 8.089583333 |
| AT4G23420 | 8.20458 | 7.311573333 | 6.823473333 | 6.265013333 | 6.508933333 |
| AT4G33320 | 6.393636667 | 6.463066667 | 6.18173 | 6.48675 | 6.5037 |
| AT4G34900 | 6.414646667 | 7.305853333 | 6.904913333 | 6.516173333 | 6.806606667 |
| AT4G38320 | 10.602 | 10.4536 | 10.55636667 | 10.63146667 | 10.95803333 |
| AT5G06420 | 6.152626667 | 6.396356667 | 5.793393333 | 5.90773 | 5.822003333 |
| AT5G25754 | 9.415083333 | 9.323293333 | 8.89269 | 8.4459 | 8.829916667 |
| AT5G28900 | 9.985623333 | 9.735153333 | 9.739726667 | 9.322216667 | 9.457013333 |
| AT5G37270 | 5.939793333 | 5.983996667 | 6.273913333 | 6.25749 | 6.043706667 |
| AT5G43620 | 7.436973333 | 6.90219 | 6.741903333 | 6.801366667 | 7.613396667 |

| vegetative_shoot_meristem | inflorescence_shoot_meristem | whole_inflorescences | whole_inflorescences_(clv3-7) | flowers | fruits |
|---|---|---|---|---|---|
| 6.30704 | 5.850233333 | 5.795143333 | 5.897746667 | 5.625643333 | 5.77372 |
| 7.923986667 | 8.394703333 | 8.12138 | 8.472316667 | 8.17326 | 8.876093333 |
| 6.48558 | 6.294903333 | 6.479853333 | 6.497233333 | 6.594063333 | 6.383973333 |
| 8.91214 | 9.628396667 | 9.142416667 | 9.134093333 | 8.970566667 | 9.09846 |
| 5.631403333 | 5.496333333 | 5.82577 | 5.859143333 | 5.970876667 | 5.932163333 |
| 5.243753333 | 5.1766 | 5.51078 | 5.80264 | 7.884593333 | 9.3087 |
| 5.852486667 | 5.579406667 | 5.55394 | 5.61937 | 5.602103333 | 5.31297 |
| 5.462766667 | 5.374003333 | 5.809206667 | 5.78477 | 6.38226 | 6.51134 |
| 4.362656667 | 4.6165 | 5.118356667 | 4.14369 | 5.173343333 | 5.609333333 |
| 8.116033333 | 7.44199 | 7.80538 | 7.515026667 | 7.791813333 | 7.93196 |
| 4.718476667 | 4.736543333 | 4.826146667 | 4.78718 | 7.4644 | 8.566436667 |
| 8.473946667 | 8.339473333 | 7.47615 | 7.35723 | 7.789956667 | 7.539943333 |
| 5.736513333 | 5.427946667 | 5.565903333 | 5.89019 | 5.617056667 | 5.699893333 |
| 7.84812 | 7.78372 | 7.94256 | 8.649826667 | 9.252306667 | 7.86604 |
| 6.755306667 | 7.062193333 | 6.603573333 | 6.563416667 | 6.580576667 | 6.538016667 |
| 6.6544 | 6.054993333 | 6.317686667 | 6.380306667 | 6.079576667 | 6.509056667 |
| 7.061083333 | 6.71551 | 6.143756667 | 6.759793333 | 6.684506667 | 6.40814 |
| 6.23312 | 6.252603333 | 6.062986667 | 6.111916667 | 6.042366667 | 6.021016667 |
| 6.02596 | 6.297926667 | 6.453463333 | 6.14129 | 6.3285 | 6.510683333 |
| 6.500916667 | 6.248643333 | 5.779776667 | 6.338656667 | 6.232603333 | 6.37948 |
| 10.20242333 | 11.36823333 | 10.6342 | 10.75876667 | 10.78386667 | 11.3475 |
| 5.87699 | 6.235363333 | 5.8972 | 6.0617 | 5.794523333 | 5.945203333 |
| 9.84784 | 10.01367333 | 9.689346667 | 9.735803333 | 10.11593333 | 10.66156667 |
| 6.71383 | 6.519966667 | 6.87991 | 6.578163333 | 7.447403333 | 6.955626667 |
| 11.60433333 | 11.39143333 | 11.41383333 | 11.4115 | 11.66516667 | 12.0976 |
| 4.77729 | 4.53126 | 4.5537 | 4.68841 | 4.449463333 | 4.36795 |
| 5.50167 | 4.854526667 | 4.921523333 | 5.677663333 | 9.612323333 | 9.944616667 |
| 5.833343333 | 6.063683333 | 5.79521 | 6.010483333 | 5.65292 | 5.75739 |
| 5.9898 | 6.538286667 | 6.365326667 | 6.53107 | 6.909403333 | 6.887526667 |
| 9.078703333 | 9.33558 | 9.196143333 | 9.49482 | 9.570793333 | 9.417843333 |
| 9.792506667 | 10.3146 | 9.76625 | 10.061 | 9.91421 | 10.06011333 |
| 6.89253 | 6.464133333 | 6.739976667 | 6.782573333 | 6.355073333 | 6.617963333 |
| 4.18419 | 4.063816667 | 4.422493333 | 3.80746 | 4.269506667 | 4.23732 |
| 7.10211 | 7.830173333 | 7.16213 | 7.30792 | 7.14528 | 7.307376667 |
| 9.488143333 | 9.56698 | 9.110906667 | 9.179713333 | 9.291986667 | 8.44235 |
| 3.912256667 | 4.485423333 | 4.006346667 | 3.785203333 | 4.10132 | 4.29558 |
| 6.276676667 | 6.193466667 | 6.064863333 | 6.295003333 | 6.177586667 | 6.015603333 |
| 5.633983333 | 5.384793333 | 5.015616667 | 5.639426667 | 5.241543333 | 5.238773333 |
| 4.707346667 | 4.45717 | 5.27338 | 6.059936667 | 4.8129 | 4.780483333 |
| 9.272316667 | 9.719583333 | 9.497036667 | 9.598096667 | 9.70401 | 9.591553333 |
| 5.32366 | 5.218653333 | 5.24848 | 5.163823333 | 5.365856667 | 5.28144 |
| 6.467153333 | 6.65691 | 6.59546 | 6.703743333 | 7.507256667 | 6.32305 |
| 5.45156 | 5.191743333 | 5.28674 | 5.347543333 | 5.41523 | 5.16909 |
| 6.243826667 | 6.144453333 | 6.26388 | 6.175486667 | 5.956916667 | 6.107686667 |
| 7.104473333 | 7.122293333 | 6.76642 | 7.02064 | 6.953533333 | 7.059053333 |

| | | | | | |
|---|---|---|---|---|---|
| 6.356276667 | 6.37835 | 6.059426667 | 5.821706667 | 5.73294 | 5.796293333 |
| 6.83695 | 6.707896667 | 6.858696667 | 6.81755 | 6.809153333 | 6.711286667 |
| 5.002346667 | 4.788493333 | 4.833763333 | 5.062396667 | 5.040236667 | 4.629736667 |
| 9.10084 | 9.158366667 | 9.453103333 | 9.396173333 | 9.11342 | 9.016543333 |
| 6.01653 | 6.08836 | 5.976306667 | 5.843786667 | 5.89547 | 6.051916667 |
| 5.786116667 | 5.712996667 | 5.684876667 | 5.570626667 | 5.622086667 | 5.548663333 |
| 5.764886667 | 5.768523333 | 5.415406667 | 5.682213333 | 5.539833333 | 5.675623333 |
| 7.844796667 | 8.133016667 | 7.640106667 | 8.09402 | 7.606936667 | 8.310326667 |
| 6.842926667 | 6.919793333 | 6.726033333 | 7.191256667 | 6.500093333 | 6.774103333 |
| 5.966456667 | 6.45611 | 6.16887 | 6.599613333 | 6.12743 | 6.062583333 |
| 6.794153333 | 6.78172 | 6.308503333 | 6.384833333 | 6.278813333 | 6.053926667 |
| 10.10761 | 10.639 | 10.61666667 | 10.79816667 | 10.80986667 | 10.9432 |
| 5.71253 | 6.057536667 | 5.72933 | 6.056066667 | 5.91876 | 6.244983333 |
| 8.618396667 | 9.04248 | 8.6372 | 8.885483333 | 8.737506667 | 9.201793333 |
| 9.249773333 | 9.643953333 | 9.060126667 | 9.375783333 | 9.245393333 | 9.737983333 |
| 6.190296667 | 5.916323333 | 6.14317 | 5.96555 | 5.664233333 | 5.994226667 |
| 6.390033333 | 6.483163333 | 7.40119 | 6.807253333 | 7.4704 | 7.30126 |

| zscore_roots | zscore_seedlings | zscore_expanding_leaves | zscore_senescing_leaves | zscore_stem | zscore_vegetative_shoot_meristem |
|---|---|---|---|---|---|
| 2.919067454 | -0.0461955 | -0.61884705 | -0.76276593 | 0.303582186 | 0.04810925 |
| -0.28443293 | 0.25786889 | 0.195781237 | -0.03097689 | 0.282203041 | -0.25288153 |
| 2.602797164 | -0.49893603 | -1.00519216 | -0.77987118 | -1.24831649 | -0.22671089 |
| 0.066700691 | 0.731428546 | 0.690062771 | -0.44983417 | -0.12427547 | -0.27177719 |
| 3.048957669 | 0.131354468 | -0.33171719 | -0.51733462 | -0.34290204 | -0.84091439 |
| -0.77907432 | -0.59398509 | -0.50338574 | -0.04952963 | -0.40707159 | -0.87070059 |
| 0.769333866 | 1.062071389 | 2.06995191 | -0.08325276 | 0.004798385 | 0.580367889 |
| -0.87152174 | -0.236054 | -1.65657575 | -0.62609971 | -1.01504979 | -0.27632034 |
| -0.99019288 | -0.1348388 | 1.195448722 | -1.00028598 | -0.39939298 | -0.11337587 |
| -1.63501131 | -0.33125074 | -0.53653027 | -0.01726989 | -0.84147245 | 1.993529465 |
| -0.04906218 | -0.35224176 | -0.41223194 | -0.36467089 | -0.3118426 | -0.4715386 |
| -0.37995425 | 0.721352758 | 0.578495912 | -0.31285865 | 1.334314768 | 0.762672056 |
| 0.183896612 | 0.193111403 | -0.7345419 | 1.019355468 | -0.47762919 | 0.940952181 |
| 1.154188093 | 0.280466605 | -0.11948436 | 1.603002461 | -0.35660889 | -0.99570485 |
| -0.34295684 | 0.031948138 | 0.751319199 | 0.765305849 | -0.64163756 | -0.73986539 |
| 0.238313033 | -0.09059761 | -0.35896626 | 0.688962307 | -1.09943147 | 1.838705016 |
| -0.57020176 | 0.201016796 | 0.958122257 | -1.15697233 | 0.968938544 | 1.472640039 |
| -0.32327429 | -0.43310199 | -0.37207448 | -0.13966897 | -0.35340441 | -0.49754457 |
| 2.18097072 | -0.34057014 | -0.69357741 | 0.221701598 | 1.021737771 | -0.72697258 |
| 0.316405382 | -0.21920297 | 0.162693772 | 2.205674285 | -1.35702474 | 0.768040735 |
| -0.46759982 | 0.222569073 | -0.02975928 | -0.61042434 | 0.003609079 | -0.75482989 |
| -0.87590994 | -0.97155393 | -0.25518093 | 0.797324649 | -0.0689452 | -0.83828164 |
| 0.20966206 | 0.735703287 | 0.821390303 | -0.51767852 | 0.140108292 | 0.453172107 |
| 1.195257123 | 0.259164834 | 1.113194882 | 1.452260417 | -0.90265184 | -0.49849397 |
| 0.517989642 | 0.63180856 | 0.367198672 | -0.64875357 | 0.317813484 | 0.745169147 |
| -0.38590206 | -0.16413485 | 0.214330392 | 1.03816956 | -0.52056842 | -0.25136305 |
| -0.68193897 | -0.36195134 | -0.67729005 | -0.71766479 | -0.29810267 | -0.20185955 |
| -0.23070686 | 0.209375629 | -0.82988149 | -0.298667 | -0.72484179 | -0.33713047 |
| -1.16686534 | -1.09663098 | -0.69467348 | -0.52714966 | 0.13143114 | -0.40419526 |
| 1.074525267 | 0.345958038 | 0.372527611 | 0.406464543 | 0.28674871 | -0.20337852 |
| 0.694138316 | 0.070794807 | 0.266963946 | -0.03739202 | 0.683770842 | 0.179698059 |
| -0.95283867 | 1.4163134 | 0.876376237 | 2.031069582 | -0.2822613 | 0.165874937 |
| 2.874176992 | -0.57059346 | -0.37671586 | -0.43355222 | -0.58478772 | -0.28877152 |
| 3.126326593 | -0.61689943 | -0.6167325 | -0.30151343 | -0.8987657 | -0.69822948 |
| -0.77054588 | 0.53968848 | 0.888856792 | 1.295317802 | -1.69447853 | -0.2087603 |
| -0.94278757 | -0.24314603 | 1.965092223 | 0.832924079 | 1.094492943 | -1.02058471 |
| 3.013923919 | 0.017559267 | 0.916681896 | -0.3796958 | -0.40277345 | -0.51411812 |
| -0.63668245 | -0.09132148 | 0.758841189 | 1.142785977 | -0.23598547 | 0.1620003 |
| -0.64577635 | -0.43346059 | -0.72725072 | -0.52084328 | -0.5463873 | -0.27725133 |
| 0.568236742 | 0.266414296 | 0.29005345 | -0.47710935 | 0.516602223 | 0.08371667 |
| -1.0480683 | 0.170015636 | -0.57247968 | 1.860321102 | 0.50659521 | 0.992414606 |
| 2.051353652 | -0.72831305 | -0.46453473 | 0.739660679 | -0.77033701 | -0.85828518 |
| 3.388542264 | 0.031552644 | -0.42667858 | -0.20908952 | -0.5405844 | -0.38801266 |
| 0.931589023 | 0.529070302 | -1.56686922 | 1.651463354 | -0.4531742 | 0.343614406 |
| 1.094134101 | 0.527714982 | -0.17060986 | -0.65758003 | -0.61003491 | 0.932034854 |

| | | | | | |
|---|---|---|---|---|---|
| -0.27014034 | 0.301487059 | 0.003162218 | -1.09986427 | -0.01124769 | 0.198993623 |
| -0.30774458 | -1.33353344 | -0.01732791 | -0.04325209 | -0.48238976 | 1.259780947 |
| -0.07834865 | -0.79775151 | 1.465478492 | 3.069768491 | -0.5973912 | -0.2013069 |
| -1.23711933 | 0.8184722 | 1.241658595 | 0.708184678 | 0.330376303 | 0.187785126 |
| 1.072909395 | 0.503979204 | -0.92180328 | -1.41855342 | 1.793866974 | -0.10309907 |
| 0.784208141 | -0.10801863 | -0.47410046 | -0.73525559 | -0.6145929 | -0.35475918 |
| -0.15600231 | -0.16253984 | -0.44927492 | 0.746405777 | -1.10108089 | -0.38873835 |
| 0.634217995 | 0.835991229 | 0.297598164 | -0.65860574 | 0.551056363 | 0.158874392 |
| 2.22980259 | 0.464730711 | -0.5000231 | -1.60384693 | -1.12172698 | -0.46157262 |
| -0.22655998 | -0.07148421 | -0.69986534 | -0.01858616 | 0.019272608 | -1.18069033 |
| -0.73786009 | 0.347506633 | -0.14078295 | -0.61421462 | -0.2605069 | -0.27567334 |
| 0.344694362 | 0.131578673 | 0.279160809 | 0.387011133 | 0.85599011 | -0.36529395 |
| -0.12229174 | 0.325348349 | -0.78206788 | -0.57207455 | -0.7295221 | -0.93058333 |
| 1.521901276 | 1.273717391 | 0.10944233 | -1.09859861 | -0.06028563 | -0.63219821 |
| 1.237538052 | 0.751226616 | 0.76010618 | -0.05052938 | 0.211191231 | -0.19118503 |
| -0.80882135 | -0.57636542 | 0.948244653 | 0.861877837 | -0.26236323 | 0.508522391 |
| 1.286927348 | -0.12232846 | -0.54471431 | -0.38801712 | 1.751836389 | -1.47195869 |

| zscore_inflorescence_shoot_meristem | zscore_whole_inflorescences | zscore_whole_inflorescences_(clv3-7) | zscore_flowers | zscore_fruits |
|---|---|---|---|---|
| -0.53556483 | -0.60595479 | -0.47485577 | -0.8225295 | -0.63332796 |
| 0.793509199 | 0.185918644 | 0.966041591 | 0.3012465 | 1.863626462 |
| -0.83580479 | -0.24500405 | -0.18948571 | 0.1198262 | -0.55128128 |
| 0.976439876 | 0.129524854 | 0.115019821 | -0.1699573 | 0.052921781 |
| -1.48652844 | 0.08812871 | 0.247648166 | 0.7817165 | 0.596672657 |
| -0.93320365 | -0.62216514 | -0.35051603 | 1.587265 | 2.912754348 |
| -0.80444727 | -0.9335912 | -0.60178933 | -0.6893503 | -2.15557344 |
| -0.46701079 | 0.46793742 | 0.415440079 | 1.6990291 | 1.976331886 |
| 0.324584052 | 1.190445306 | -0.49116253 | 1.2853147 | 2.037535124 |
| -0.29114164 | 0.940569429 | -0.04358372 | 0.8945852 | 1.369612568 |
| -0.4552902 | -0.37470473 | -0.40974971 | 1.9980296 | 2.989155068 |
| 0.656736045 | -0.02337672 | -0.11706005 | 0.2238353 | 0.026878683 |
| -0.49509696 | 0.14694437 | 1.656153362 | 0.3850086 | 0.77052509 |
| -1.08909103 | -0.85875779 | 0.166846753 | 1.0405006 | -0.96971913 |
| -0.05199418 | -1.07996811 | -1.16997729 | -1.131514 | -1.22691013 |
| -0.7204819 | 0.401096118 | 0.668454313 | -0.6155225 | 1.218156767 |
| 0.575563174 | -0.90865635 | 0.690518669 | 0.4950813 | -0.22234132 |
| -0.47556098 | -0.6895108 | -0.63430171 | -0.7127769 | -0.73686674 |
| 0.318580928 | 0.916528869 | -0.28359587 | 0.4361176 | 1.136506459 |
| 0.019924847 | -1.37049799 | 0.286859144 | -0.0276417 | 0.407920628 |
| 2.023824871 | 0.27429003 | 0.571188976 | 0.6310137 | 1.97440792 |
| 0.033591903 | -0.78911346 | -0.38890735 | -1.0389118 | -0.67232789 |
| 0.683812643 | 0.232740022 | 0.297351824 | 0.8260356 | 1.58490088 |
| -0.88538258 | -0.16705192 | -0.76924083 | 0.9654815 | -0.0159459 |
| 0.501222358 | 0.526888906 | 0.524215308 | 0.8148737 | 1.310367922 |
| -0.77356486 | -0.72593567 | -0.44001198 | -0.9471793 | -1.12019241 |
| -0.60659307 | -0.56469231 | -0.09179058 | 2.3690071 | 2.576828512 |
| 0.453206353 | -0.46797261 | 0.270667849 | -0.9561945 | -0.59773964 |
| 0.674768825 | 0.334527869 | 0.660572446 | 1.4048168 | 1.361781725 |
| 0.182583965 | -0.0269225 | 0.421845345 | 0.5359968 | 0.306186318 |
| 0.719178693 | 0.152566966 | 0.45713304 | 0.3054545 | 0.456216844 |
| -1.24218854 | -0.33554068 | -0.19553303 | -1.6006493 | -0.73657675 |
| -0.41483717 | -0.03919909 | -0.68331665 | -0.1994203 | -0.23312906 |
| 0.191039027 | -0.62492007 | -0.44684977 | -0.6455009 | -0.4475134 |
| -0.12723024 | -0.59888496 | -0.52772757 | -0.4116185 | -1.29028234 |
| 0.615171994 | -0.75206186 | -1.38318141 | -0.4810181 | 0.073379313 |
| -0.69214264 | -0.96728442 | -0.47490893 | -0.7261173 | -1.07267426 |
| -0.66190993 | -1.88253849 | 0.179997884 | -1.1355451 | -1.14470366 |
| -0.61627086 | 0.489792046 | 1.555671115 | -0.1342138 | -0.17814234 |
| 0.776271618 | 0.431676756 | 0.588159697 | 0.7521576 | 0.578027893 |
| 0.176490811 | 0.408250259 | -0.24954977 | 1.3202915 | 0.664356362 |
| -0.51564723 | -0.62660566 | -0.43108168 | 1.0197984 | -1.11848827 |
| -0.65977235 | -0.56040896 | -0.49681067 | -0.4260126 | -0.68346699 |
| -0.26508439 | 0.466448565 | -0.07499363 | -1.4138166 | -0.49029396 |
| 0.971108827 | 0.190784191 | 0.748213227 | 0.6010682 | 0.83244227 |

| | | | | |
|---|---|---|---|---|
| 0.251713177 | -0.50999759 | -1.07776377 | -1.2897725 | -1.13846052 |
| 0.551467239 | 1.379138283 | 1.153303372 | 1.107218 | 0.570073372 |
| -0.46688647 | -0.41066669 | -0.12673218 | -0.1542522 | -0.66404274 |
| 0.232359882 | 0.460737704 | 0.416625278 | 0.1975328 | 0.122467535 |
| 0.090143312 | -0.21131087 | -0.56782598 | -0.4287837 | -0.00789924 |
| -0.54421281 | -0.61707162 | -0.91309292 | -0.7797603 | -0.96999983 |
| -0.37832602 | -1.38935253 | -0.62544463 | -1.0331001 | -0.64431279 |
| 0.620642548 | -0.16906719 | 0.558164514 | -0.2222101 | 0.9047176 |
| -0.30964185 | -0.69261808 | 0.226918863 | -1.1391997 | -0.59760535 |
| -0.08702231 | -0.72858887 | 0.233500386 | -0.8211474 | -0.96598606 |
| -0.29081543 | -0.86712802 | -0.77416861 | -0.9032863 | -1.17716726 |
| 0.397829675 | 0.365757008 | 0.626407261 | 0.6432095 | 0.834688115 |
| -0.2969362 | -0.89972806 | -0.29963604 | -0.5518166 | 0.047332628 |
| 0.51444793 | -0.58135733 | 0.089956782 | -0.3101459 | 0.945202935 |
| 0.574153102 | -0.55940215 | 0.053475425 | -0.1996892 | 0.756721331 |
| -0.93224509 | 0.260693277 | -0.67337252 | -2.2579328 | -0.52256803 |
| -1.22654343 | 1.192631458 | -0.37250463 | 1.375013 | 0.9292969 |
