# Supplementary material for: Divergent Evolutionary and Expression Patterns between Lineage Specific New Duplicate Genes and Their Parental Paralogs in Arabidopsis thaliana
Source: PLoS One. 2013 Aug 29;8(8):e72362. doi: 10.1371/journal.pone.0072362 (PMC3756979; doi:10.1371/journal.pone.0072362)
Supplement: Table S8 — Tiling array data of 62 parental genes. (PDF) [file pone.0072362.s013.pdf]

Table S8 Tiling array data of 62 parental genes

parental_gene	roots	seedlings	expanding_leaves	senescing_leaves
AT1G14190	8.74196	7.347163333	7.044936667	6.529816667
AT3G55490	8.255903333	7.954186667	7.831433333	7.820413333
AT1G21540	6.70886	8.838243333	8.246966667	7.35238
AT1G07780	8.58239	8.833076667	8.37352	7.098073333
AT1G32720	5.443583333	5.428676667	5.544853333	5.278326667
AT1G29820	8.743543333	8.065953333	7.57209	7.573806667
AT1G30972	3.96034	3.79239	4.106836667	4.451833333
AT1G31690	6.55487	9.318946667	10.13540333	5.921453333
AT5G08055	5.505616667	4.807636667	5.177673333	5.417926667
AT3G11990	4.517766667	4.540013333	4.914476667	4.859183333
AT4G28310	9.644346667	9.757356667	9.167246667	7.11332
AT1G56000	8.7001	9.533993333	9.499703333	9.55629
AT1G61440	6.100963333	5.962566667	6.049726667	6.32833
AT1G62000	4.97684	5.487776667	5.384196667	5.668713333
AT1G68260	9.861983333	10.63576667	10.6995	9.279473333
AT1G55860	6.066063333	6.091693333	6.074193333	6.306993333
AT2G16530	11.60073333	9.528173333	10.00092667	10.2593
AT1G49715	6.198863333	5.517636667	5.539913333	6.133866667
AT1G74280	8.311173333	7.1772	7.298196667	7.60251
AT1G80980	7.826733333	8.06533	7.477433333	8.542516667
AT2G06904	5.219853333	4.938363333	4.975833333	5.257113333
AT5G04800	11.86726667	11.9524	11.59893333	10.9395
AT1G72510	10.41549667	9.58851	9.632796667	10.20626667
AT5G18380	12.7425	12.8101	12.6299	12.24753333
AT4G02000	5.854466667	6.072966667	6.09428	6.557206667
AT4G35165	4.412546667	4.4359	4.47659	4.5691
AT3G44713	5.00982	5.443323333	4.75161	4.722656667
AT4G04030	7.51361	5.734403333	5.252003333	5.8833
AT2G20120	10.15564333	9.815026667	9.79234	9.68022
AT2G30910	9.650683333	9.189093333	9.4028	9.3788
AT2G43445	6.68695	7.761533333	6.48536	6.728953333
AT3G02610	8.585613333	7.04808	7.148303333	7.883176667
AT3G05165	12.06536667	11.0652	11.0403	12.10536667
AT1G18330	8.00531	8.52559	9.09693	9.103046667
AT3G17740	8.17619	7.77923	7.87611	8.151643333
AT3G23530	7.626333333	8.324723333	8.223496667	7.04813
AT3G55650	5.491356667	5.599633333	5.384376667	5.780153333
AT2G14282	5.351266667	5.336756667	4.681563333	5.862216667
AT5G62950	10.8467	10.3714	10.3447	10.40236667
AT5G36150	5.916576667	5.910826667	6.24364	6.01023
AT3G29250	12.7586	7.879296667	7.758746667	8.558226667
AT3G45710	9.671793333	6.91398	6.372606667	6.097593333
AT3G47750	5.793236667	6.46299	6.094896667	6.247713333
AT5G01430	11.24903333	10.87366667	10.82383333	10.75786667
AT5G01630	6.530263333	6.503706667	6.422703333	5.694933333

AT5G59390	4.551703333	4.642206667	5.028383333	4.930776667
AT4G10880	5.622806667	5.677293333	5.853563333	5.07935
AT2G05310	6.38793	11.0924	11.42986667	11.16313333
AT4G12620	8.401596667	8.125873333	7.59405	6.679906667
AT4G15215	6.276206667	5.953196667	5.895036667	6.06338
AT4G19750	6.360586667	6.160266667	6.072393333	6.319043333
AT3G18240	8.78699	8.273556667	7.744236667	6.85711
AT4G23430	10.52446667	10.19626667	10.07893333	9.728916667
AT4G34890	7.619643333	7.406386667	7.30233	7.79242
AT4G37680	6.99906	7.053616667	7.308996667	7.444916667
AT1G01350	6.73763	7.018526667	6.637803333	7.101356667
AT5G25757	10.33975667	9.832916667	9.755113333	8.85914
AT5G28850	9.213196667	8.97268	9.042103333	8.795653333
AT3G42565	5.154433333	5.572886667	5.170873333	5.162636667
AT5G37230	5.968586667	5.733503333	5.8679	5.959576667
AT5G39200	4.216566667	4.319446667	4.9191	4.870743333
AT1G66500	8.30769	7.20655	7.14875	7.932256667
AT1G66500	8.30769	7.20655	7.14875	7.932256667

stem	vegetative_shoot_meristem	inflorescence_shoot_meristem	whole_inflorescences	whole_inflorescences_(clv3-7)	flowers	fruits
8.010843333	7.4828767	6.88672	6.71273	7.04831333	6.8261	7.078
7.783036667	7.7460767	8.34422333	8.157237	8.12980667	8.1397	8.199
6.536833333	6.9957333	8.83665667	10.25719	10.9945333	8.023	8.392
7.760656667	7.6105533	8.71843667	8.038563	8.33366	7.9602	8.413
5.347006667	5.6041933	5.54940333	5.06272	5.43226667	4.6981	5.621
7.969386667	7.1781433	7.43465	7.252113	7.79724333	7.8258	8.244
4.02185	4.0595267	4.07396	4.281327	4.02408667	5.8586	6.797
5.67947	6.4119767	5.39617333	5.60196	5.67094667	5.535	5.347
5.09832	4.1825667	4.80232667	4.833307	5.46591	5.8946	7.372
4.739666667	4.8577867	4.55572	4.864357	4.83044	7.5262	8.502
9.227316667	9.8887133	10.5487667	10.3184	10.1200333	10.039	10.61
9.43141	9.1901	9.19432	8.995963	9.04881333	8.7227	9.287
5.834973333	5.9675667	6.02958	5.83582	5.87859667	5.942	5.744
6.198826667	5.7303333	6.02870333	5.610653	5.39480333	5.2016	5.327
10.6449	10.209507	10.6806333	10.3717	10.5544333	10.385	10.39
6.11795	5.96696	6.02922667	5.76295	5.98574	5.8616	5.81
10.09964667	9.3255233	10.3691333	9.701343	9.85482667	10.717	9.985
6.18182	6.0630567	5.64315667	6.181	7.01019	12.008	12.14
7.029473333	7.0867767	7.29356333	6.877897	7.11596667	7.1583	7.375
8.014953333	7.7666733	8.41288	7.763187	8.19897333	8.0722	8.113
5.3053	4.8947533	5.26861667	4.963397	5.00621333	4.8491	5.014
11.6383	11.919467	11.8289	11.8412	11.8299333	11.809	12.33
8.939893333	9.0370067	8.47653667	8.814573	8.94452	9.9365	9.73
12.56656667	12.775867	12.8968667	12.72367	12.6772667	12.794	12.73
6.062856667	6.35177	6.13491667	5.93927	6.03339333	5.9532	5.912
4.241326667	4.41612	4.41290333	4.593557	4.58763333	6.619	7.66
5.39924	4.7898167	4.94196	4.812943	4.9032	4.8376	5.022
7.84065	5.76452	5.37644667	6.049707	5.76088	7.909	6.623
9.804053333	9.6343167	10.2154	9.755653	9.94244667	9.6082	9.972
9.60083	9.5067833	9.51487333	9.300877	9.48168333	9.2078	9.662
6.00115	7.1412	6.63004667	6.652937	6.42202333	6.1981	6.416
6.82453	6.83071	7.58592333	7.079793	7.24878	7.2183	7.585
10.53976667	11.263233	10.92	10.9207	11.0131333	12.015	10.99
8.377653333	8.0942267	8.61877	8.641223	8.63733333	8.0612	7.793
7.93861	7.6545067	8.25374333	7.521637	7.95303	7.6603	7.565
8.106616667	7.71968	7.76242333	7.464197	7.55075333	7.1578	7.62
5.430623333	5.39528	5.34725667	5.393233	5.43398	5.4285	5.3
5.456216667	6.17964	5.67876333	6.50902	9.67064333	8.8251	7.253
10.4671	10.26612	10.965	10.646	10.7553	10.749	11.05
5.805513333	5.9779333	6.11036667	6.494437	7.28857	5.9563	5.796
8.34266	7.9203333	7.19588	6.846957	6.83977	11.371	7.187
6.39755	6.0463033	5.83342	6.39189	6.74126	6.4645	6.205
5.812176667	6.3545167	5.91798333	6.047347	5.85253333	5.8997	5.965
10.92363333	11.001633	11.3334	11.10317	11.2906	11.186	11.29
6.577103333	6.5496067	6.53522	6.389107	6.36809333	6.0646	6.148

4.77542	4.68382	6.20866667	6.213493	6.62017	6.2501	6.681
5.413923333	5.3430767	5.68090667	5.95083	5.83566333	5.3923	5.649
10.559	10.316123	10.6032333	10.49827	10.6187667	10.612	10.32
8.351216667	8.14824	8.07546333	7.65827	7.54897	7.268	7.432
5.904086667	6.0381367	6.06698	5.943027	5.79331	5.7282	5.911
6.531426667	6.19561	6.04755333	5.85316	6.22684667	5.9968	6.04
8.03839	8.1477967	8.61465	7.809347	8.21250667	7.9049	8.474
9.09106	9.3421567	9.70063333	9.15336	9.48627333	9.4428	9.443
7.374836667	7.0182433	7.18107	6.851553	7.20550667	7.5609	6.802
7.640096667	6.9661867	7.28952	7.258847	7.57831	7.6156	7.475
6.81537	6.6846	6.74312667	6.621403	7.21919333	6.6057	6.926
9.318423333	9.4200933	9.70987	8.964347	9.54587667	9.248	9.449
8.95291	8.96625	9.14388667	8.665303	8.74120667	8.7013	8.724
5.810063333	5.40949	5.38931333	5.166283	5.07912667	6.7501	6.796
6.25623	6.3470267	5.96823333	6.504523	6.28916667	6.2503	6.352
4.79496	4.9616933	3.67893	4.197037	4.26221333	4.4941	4.914
8.1292	6.7359133	7.42607667	8.42655	7.37327	8.4583	7.92
8.1292	6.7359133	7.42607667	8.42655	7.37327	8.4583	7.92

zscore_roots	zscore_seedlings	zscore_expanding_leaves	zscore_senescing_leaves	zscore_stem	zscore_vegetative_shoot_meristem
2.392867469	-0.028371686	-0.5530095	-1.447211	1.12371596	0.20721422
0.764896587	0.142272322	-0.1110422	-0.133783	-0.2109138	-0.2871847
-1.30699701	0.189193376	-0.2262614	-0.854834	-1.4278699	-1.1054283
0.740153202	1.015649449	0.5106121	-0.891061	-0.1629042	-0.3278627
0.246507682	0.207237304	0.5132951	-0.188847	-0.0079155	0.66962142
2.21036936	0.64750065	-0.4916005	-0.487641	0.42476858	-1.4002428
-0.48361962	-0.691239907	-0.30252	0.1239659	-0.4075808	-0.3610048
0.096193778	2.138918702	2.7423015	-0.371918	-0.5507497	-0.0094081
0.073172528	-0.760989515	-0.3187554	-0.031627	-0.4135913	-1.5080162
-0.57548021	-0.55590538	-0.2264153	-0.275068	-0.3802305	-0.2762968
0.296992442	0.401643241	-0.1448171	-2.046818	-0.0891904	0.52328363
-1.79748542	1.2505522	1.1252158	1.3320507	0.87559074	-0.0064429
0.245158954	-0.394796949	0.008237	1.2965184	-0.9847975	-0.3716766
-1.85453663	-0.529116968	-0.7978136	-0.05975	1.31541625	0.10009874
-0.30890786	0.704085676	0.7875218	-1.071497	0.71604252	0.1460501
-0.39022188	-0.350428951	-0.3775993	-0.016156	-0.3096631	-0.5440889
1.590707086	-0.134262713	0.2592049	0.4742463	0.34136851	-0.3029262
-0.15905986	-0.461703263	-0.4518066	-0.187935	-0.1666316	-0.2193937
2.351729484	-0.002140813	0.2490207	0.8807059	-0.3087877	-0.1898391
-0.70960684	0.063896112	-1.8419973	1.6108804	-0.0994192	-0.9043145
0.055028586	-0.894022908	-0.7676917	0.1806517	0.34311444	-1.0410553
0.597817702	0.742604721	0.1414608	-0.980043	0.20841205	0.68659471
1.778782483	0.327017365	0.4047621	1.4114817	-0.8116214	-0.6411401
0.44650032	0.527808014	0.3110677	-0.148834	0.23489183	0.48663296
-1.21248058	-0.28631062	-0.1959684	1.7662685	-0.3291645	0.89547095
-0.42817662	-0.403422836	-0.3602927	-0.262235	-0.6096643	-0.424389
-0.42375236	0.576652178	-1.0196289	-1.086445	0.47492017	-0.9314586
1.552518859	-0.553772219	-1.1248553	-0.377503	1.93968098	-0.518119
0.666516322	0.247984794	0.2201086	0.0823416	0.23450135	0.02593802
1.055247184	-0.322854433	0.3151782	0.243525	0.90640739	0.62562604
0.172776852	2.879420535	-0.3349849	0.2785742	-1.5546055	1.31693464
2.825431738	-0.775762868	-0.5410208	1.1801919	-1.2993594	-1.2848846
0.848156453	-0.56825425	-0.603517	0.9048034	-1.3123596	-0.2878045
-0.94866521	0.054540749	1.1562008	1.167995	-0.2307113	-0.7772157
0.103976703	-0.722481175	-0.5207801	0.0528713	-0.3906572	-0.9821511
-0.57114928	0.612713524	0.441121	-1.55128	0.24299407	-0.4129144
-0.31928185	-0.069319231	-0.566251	0.3474211	-0.4594881	-0.5410801
-0.68602867	-0.694646276	-1.0837708	-0.382571	-0.623698	-0.1940511
0.704353586	-0.010158543	-0.0502963	0.0363932	0.13370599	-0.1684246
-0.64743415	-0.657814576	-0.0569894	-0.478363	-0.8479359	-0.5366675
2.231081761	-0.362506818	-0.426585	-0.001622	-0.1162065	-0.3406938
2.895814177	0.111269293	-0.4353516	-0.713031	-0.4101665	-0.7648178
-1.21352471	1.101758657	-0.1707104	0.3575645	-1.1480507	0.72677508
0.550354513	0.183671191	0.1349907	0.07055	0.23248198	0.30867761
-0.05584902	-0.10923523	-0.2720742	-1.735092	0.03831227	-0.0169636

-1.13009798	-1.035513218	-0.6319211	-0.73393	-0.8962924	-0.9920232
0.388867256	0.57207626	1.1647763	-1.438482	-0.3134937	-0.5517125
-2.70816053	0.717793511	0.9635481	0.7693039	0.32935361	0.15248261
1.149883974	0.619044189	-0.4048553	-2.164821	1.05288925	0.66210589
1.778664862	-0.38605056	-0.7758213	0.3523648	-0.7151709	0.18319166
0.002651767	-0.4307859	-0.6209197	-0.087237	0.37230278	-0.3543126
1.29246518	0.544973503	-0.2256471	-1.517187	0.20260164	0.36188342
2.056665407	1.279904355	1.0022079	0.1738127	-1.3358223	-0.7415441
-0.18516156	-0.411699013	-0.5222359	-0.001625	-0.4452138	-0.8240144
-0.39053616	-0.258246413	0.3610025	0.6905831	1.16385823	-0.4702479
-0.65048601	0.013783741	-0.886558	0.2096617	-0.466645	-0.7758924
1.672680591	0.70949667	0.5616415	-1.14104	-0.2682314	-0.0750207
0.889608623	0.423827878	0.558272	0.0810009	0.38554161	0.41137564
-0.74950277	-0.032975171	-0.7213522	-0.735456	0.37314808	-0.3127632
-0.06835918	-0.769552521	-0.3686817	-0.095234	0.78960716	1.06043035
-0.3110709	-0.098952739	1.1374136	1.0377117	0.88146148	1.22523256
1.176980058	-0.628040247	-0.7227877	0.561559	0.88439413	-1.3995214
1.176980058	-0.628040247	-0.7227877	0.561559	0.88439413	-1.3995214

zscore_inflorescence_shoot_meristem	zscore_whole_inflorescences	zscore_whole_inflorescences_(clv3-7)	zscore_flowers	zscore_fruits
-0.827659104	-1.1296898	-0.54714789	-0.93285	-0.4964478
0.947154252	0.561287481	0.504682774	0.525051	0.647030302
0.18807852	1.186200039	1.704289467	-0.38366	-0.12431533
0.88966393	0.142505916	0.466807265	0.056373	0.553504732
0.525281706	-0.75684522	0.216694861	-1.71748	0.713668877
-0.80860736	-1.22963017	0.02771811	0.093515	1.058397875
-0.343162245	-0.08681496	-0.40481581	1.863064	3.022779368
-0.760113353	-0.60803163	-0.55704868	-0.65752	-0.79664834
-0.767335543	-0.73031107	0.025718741	0.538002	2.303779374
-0.542085094	-0.27051588	-0.30035913	2.071653	2.93024135
1.134513683	0.921186936	0.737493191	0.662506	1.189550811
0.008982017	-0.71604906	-0.52287233	-1.71478	0.346660548
-0.084922591	-0.98088244	-0.78308013	-0.48981	-1.40524877
0.874099689	-0.21036288	-0.77029888	-1.27141	-0.94615277
0.762822588	0.358384475	0.597608659	0.375665	0.386967445
-0.447414188	-0.86083317	-0.51493123	-0.70766	-0.78749908
0.565659413	0.009864849	0.137607407	0.855102	0.246279439
-0.405939446	-0.16699587	0.201382077	2.421884	2.480896944
0.239402957	-0.62342635	-0.12924726	-0.04129	0.407803435
1.190613238	-0.91561782	0.497152427	0.086017	0.219657879
0.219435543	-0.80962232	-0.66526472	-1.195	-0.63743837
0.53256717	0.55348589	0.534324569	0.498156	1.376969145
-1.62503841	-1.03161912	-0.80349931	0.937921	0.575494979
0.632168925	0.423848028	0.368039197	0.508564	0.428137931
-0.023719182	-0.85301926	-0.45405265	-0.79403	-0.96887881
-0.427798565	-0.23631183	-0.24259038	1.910597	3.014252828
-0.580354274	-0.87808868	-0.66980152	-0.82116	-0.39615202
-0.977534645	-0.1805044	-0.52242816	2.020592	0.497672541
0.739942115	0.17503002	0.404551669	-0.0061	0.441291189
0.64977917	0.010880684	0.550688651	-0.26687	1.088366924
0.029449627	0.0871046	-0.49451629	-1.05847	-0.50910845
0.483968202	-0.70148423	-0.30568541	-0.37708	0.481985148
-0.773882812	-0.77289149	-0.64198974	0.776923	-0.66870824
0.234210795	0.277505402	0.270004688	-0.84094	-1.35892917
0.265440234	-1.25878216	-0.3606352	-0.97007	-1.16788345
-0.34045885	-0.84599223	-0.69926731	-1.36535	-0.58233715
-0.651944593	-0.54580494	-0.45173903	-0.46439	-0.76202418
-0.491525782	0.001570293	1.879283675	1.377109	0.443592259
0.882192393	0.402643962	0.566953189	0.557783	1.016636326
-0.297586681	0.39577168	1.829413949	-0.57578	-0.86486949
-0.725776232	-0.91124607	-0.91506613	1.493398	-0.73042374
-0.979764601	-0.41588134	-0.06312489	-0.34259	-0.60455258
-0.782285521	-0.33508689	-1.0085409	-0.84544	-0.61906131
0.632769528	0.407862175	0.590959619	0.488714	0.592750542
-0.045884756	-0.33961277	-0.38185536	-0.9919	-0.8233386

0.601589413	0.606633747	1.031650308	0.644849	1.095261878
0.584225934	1.491831189	1.104588372	-0.38607	0.477815418
0.361565813	0.285125539	0.372877711	0.368168	0.158267212
0.521991707	-0.28121496	-0.49164615	-1.03261	-0.71652964
0.37649096	-0.45420683	-1.45756269	-1.89371	-0.66687404
-0.674666711	-1.09528069	-0.286725	-0.78448	-0.6919044
1.041560715	-0.13085545	0.456092701	0.008297	0.836889798
0.106873674	-1.18837499	-0.40045874	-0.50327	-0.50348254
-0.651047513	-1.00108518	-0.62508903	-0.24762	-1.05377407
0.313775182	0.239398068	1.014037081	1.104386	0.762430347
-0.637487391	-0.92534103	0.488324073	-0.96237	-0.205971
0.475662381	-0.94110837	0.164014244	-0.4021	-0.02014435
0.755383981	-0.17143288	-0.02443969	-0.10181	-0.05716151
-0.347312158	-0.72921174	-0.87845217	1.982818	2.060734509
-0.069413086	1.530202545	0.887848799	0.771999	1.076199123
-1.419571121	-0.35133789	-0.21695653	0.261127	1.126609711
-0.268185831	1.371818783	-0.35474804	1.423892	0.540899212
-0.268185831	1.371818783	-0.35474804	1.423892	0.540899212
