# Supplementary material for: Divergent Evolutionary and Expression Patterns between Lineage Specific New Duplicate Genes and Their Parental Paralogs in Arabidopsis thaliana
Source: PLoS One. 2013 Aug 29;8(8):e72362. doi: 10.1371/journal.pone.0072362 (PMC3756979; doi:10.1371/journal.pone.0072362)
Supplement: Table S9 — The MPSS data of 100 new genes. (PDF) [file pone.0072362.s014.pdf]

Table S9 The MPSS data of 100 new genes

gene_name	CAF	INF	LEF	ROF	SIF
AT1G14185	0	0	2	29	0
AT1G19080	0	0	0	0	0
AT1G21530	0	0	0	1	0
AT1G24880	0	0	0	0	0
AT1G25112	0	0	0	0	0
AT1G29410	0	0	4	14	4
AT1G29620	N/A				
AT1G29830	0	0	0	4	0
AT1G30974	0	0	0	0	57
AT1G31670	0	0	0	0	0
AT1G33607	N/A				
AT1G34795	0	0	0	0	0
AT1G34820	0	0	0	0	0
AT1G34830	0	0	0	0	0
AT1G34850	0	0	0	0	0
AT1G34930	0	0	0	0	0
AT1G43100	N/A				
AT1G45190	N/A				
AT1G52270	0	0	4	8	0
AT1G53890	0	0	0	0	0
AT1G55980	0	0	0	0	0
AT1G59077	0	0	0	0	0
AT1G59406	0	0	0	0	0
AT1G61200	N/A				
AT1G61430	0	19	4	0	6
AT1G62080	N/A				
AT1G68280	0	0	0	0	0
AT1G70320	268	181	121	98	95
AT1G72590	N/A				
AT1G73607	0	0	0	0	0
AT1G74290	0	0	0	6	0
AT1G80700	71	61	11	142	92
AT2G02840	3	0	0	10	6
AT2G04390	0	0	0	0	0
AT2G07692	N/A				
AT2G07713	N/A				
AT2G07715	0	0	0	0	0
AT2G07725	0	0	0	0	0
AT2G07727	0	0	0	0	0
AT2G07741	0	0	0	0	0
AT2G07771	0	0	0	0	0
AT2G07776	0	0	0	0	0
AT2G09970	0	0	0	0	0
AT2G09990	0	21	4	7	2
AT2G13450	0	0	0	0	0
AT2G14378	N/A				
AT2G14800	0	0	0	0	0

AT2G19850	0	0	0	0	0
AT2G20130	54	74	20	74	42
AT2G31300	45	8	18	35	15
AT2G43440	0	0	0	0	0
AT3G02240	0	0	4	23	0
AT3G02620	2	0	2	5	7
AT3G05160	0	7	7	0	0
AT3G10113	0	0	0	0	0
AT3G17712	N/A				
AT3G23510	0	0	0	0	0
AT3G25960	0	0	0	0	0
AT3G27503	N/A				
AT3G28300	0	0	0	0	0
AT3G28956	0	0	0	0	0
AT3G29255	0	0	0	0	0
AT3G29260	0	0	0	0	0
AT3G45700	0	0	0	42	0
AT3G47760	0	0	0	0	0
AT3G49420	88	0	40	48	46
AT4G00020	0	0	0	0	0
AT4G01180	N/A				
AT4G10860	N/A				
AT4G13500	0	0	0	0	0
AT4G14700	0	10	0	2	1
AT4G15230	4	0	0	4	0
AT4G19760	N/A				
AT4G21460	20	88	20	18	62
AT4G23420	12	0	0	1	0
AT4G33320	N/A				
AT4G34900	0	0	0	0	0
AT4G38320	0	0	0	0	0
AT5G06420	0	0	0	0	0
AT5G25754	0	0	0	0	0
AT5G28900	25	51	36	2	26
AT5G36670	0	0	0	0	0
AT5G36710	0	0	0	0	0
AT5G36722	N/A				
AT5G36738	N/A				
AT5G36739	N/A				
AT5G36780	0	0	0	0	0
AT5G37270	N/A				
AT5G39140	0	0	0	0	0
AT5G39160	0	0	0	0	0
AT5G43620	0	0	0	0	0
AT5G50530	0	0	0	0	0
AT5G50600	0	0	0	0	0
ATMG00200	N/A				
ATMG00440	0	0	0	0	0
ATMG00550	N/A				

ATMG00620	0	0	0	0	0
ATMG01090	0	0	0	0	0
ATMG01140	0	0	0	0	0
ATMG01150	0	0	0	0	0

AP1	AP3	AGM	INS	ROS	SAP
0	2	0	0	18	0
0	3	5	0	8	0
0	0	0	2	4	0
0	0	0	0	0	0
0	0	0	0	0	0
0	4	0	0	0	0
0	0	0	0	0	0
0	0	0	0	0	0
0	0	0	0	0	0
0	0	0	0	0	0
0	0	0	0	0	0
0	0	0	0	0	0
0	0	0	0	0	0
0	0	0	0	0	0
0	2	0	0	0	4
0	0	0	0	0	0
0	0	0	0	0	0
0	0	0	0	0	0
0	0	0	0	0	0
1	2	0	0	2	0
0	0	0	0	0	0
351	189	296	129	178	125
0	0	0	0	0	0
0	0	0	0	0	0
4	31	9	7	12	15
0	0	0	0	0	0
0	0	0	0	0	0
0	0	0	0	0	0
0	0	0	0	0	0
0	0	0	0	0	0
0	0	0	0	0	0
0	0	0	0	0	0
0	0	0	0	0	0
0	0	0	0	0	0
3	13	6	7	0	2
0	0	0	0	0	0
4	0	0	0	0	0

0	0	0	0	0	0
0	0	0	0	0	0
4	0	2	0	0	0
0	0	0	0	0	0
0	0	0	0	0	0
4	3	1	0	0	0
4	0	0	0	0	2
0	0	0	0	0	0
0	0	0	0	0	0
0	0	0	0	0	0
0	0	0	0	0	0
0	0	0	0	0	0
0	0	0	0	0	0
0	0	0	0	0	0
0	0	0	0	1	0
0	0	0	0	0	0
0	4	5	10	18	6
0	0	0	0	0	0
0	0	0	0	0	0
2	3	0	2	0	0
0	0	0	0	0	0
16	26	0	14	0	0
3	0	0	0	63	9
0	0	0	0	0	0
0	0	0	0	0	0
0	0	0	0	0	0
0	0	0	0	0	0
46	35	17	23	32	33
0	0	0	0	0	0
0	0	0	0	0	0
0	0	0	0	0	0
0	0	0	0	0	0
0	0	0	0	0	0
0	0	0	0	0	0
0	0	0	0	0	0
0	0	0	0	0	0
0	0	0	0	0	0

0	0	0	0	0	0
0	0	0	0	0	0
0	0	0	0	0	0
0	0	0	0	0	0

S04	S52	LES	GSE	CAS	SIS
0	0	0	0	0	0
12	0	0	0	0	0
0	0	0	0	0	0
0	0	0	0	0	0
0	0	0	0	0	0
0	0	0	0	0	34
0	0	0	0	13	0
0	0	0	0	0	0
0	0	0	0	0	0
0	0	0	0	0	0
0	0	0	0	0	0
0	0	0	0	0	0
0	0	0	0	0	0
0	0	0	0	0	0
0	0	6	4	0	0
0	0	0	0	0	0
0	0	0	0	0	0
0	0	0	0	0	0
0	0	0	0	0	0
0	0	0	0	3	0
0	0	0	0	0	0
53	36	108	78	500	266
0	0	0	0	0	0
0	0	0	0	0	0
0	0	9	0	20	16
0	0	0	0	0	0
0	0	0	0	0	0
0	0	0	0	0	0
0	0	0	0	0	0
0	0	0	0	0	0
0	0	0	0	0	0
0	0	0	0	0	0
0	0	0	0	0	0
0	0	0	0	0	0
0	0	0	0	0	7
37	0	0	0	0	0
0	0	0	0	0	0

0	0	0	0	0	0
0	0	0	0	0	0
0	0	0	5	8	0
0	0	0	0	0	0
0	0	0	0	0	0
0	5	0	1	0	0
0	0	23	0	0	0
0	0	0	0	0	0
0	0	0	0	0	0
0	0	0	0	0	0
0	0	0	0	0	0
0	0	0	0	0	0
0	0	0	0	0	0
0	0	0	0	0	0
0	0	0	0	0	0
0	0	0	0	0	0
0	0	22	2	57	12
0	0	0	10	0	0
0	0	0	0	0	0
0	0	2	0	0	4
0	0	0	11	0	0
0	0	16	0	12	12
0	0	2	3	95	2
0	0	0	0	0	0
0	0	0	0	0	0
0	0	0	0	0	0
0	0	0	0	0	0
15	4	0	19	63	0
0	0	0	0	0	0
0	0	0	0	0	0
0	0	0	0	0	0
0	0	0	0	0	0
0	0	0	0	0	0
0	0	0	0	0	4
0	0	0	0	0	0
0	0	0	0	0	0
0	0	0	0	0	0

0	0	0	0	0	0
0	0	0	0	0	0
0	0	0	0	0	0
0	0	0	0	0	0
