# Supplementary material for: Divergent Evolutionary and Expression Patterns between Lineage Specific New Duplicate Genes and Their Parental Paralogs in Arabidopsis thaliana
Source: PLoS One. 2013 Aug 29;8(8):e72362. doi: 10.1371/journal.pone.0072362 (PMC3756979; doi:10.1371/journal.pone.0072362)
Supplement: Table S10 — The MPSS data of 100 old genes. (PDF) [file pone.0072362.s015.pdf]

Table S10 The MPSS data of 100 old genes

gene_name	CAF	INF	LEF	ROF	SIF
AT1G14190	0	0	4	7	19
AT3G55490	0	0	0	0	2
AT1G21540	0	0	0	0	0
AT1G25054	0	0	0	0	0
AT1G25025	0	0	0	0	0
AT1G07780	18	8	10	0	3
AT1G32720	N/A				
AT1G29820	N/A				
AT1G30972	0	0	0	0	76
AT1G31690	0	0	31	0	0
AT5G08055	N/A				
AT1G34815	0	0	0	0	0
AT1G34825	0	0	0	0	0
AT1G34815	0	0	0	0	0
AT1G34840	0	0	0	0	0
AT1G34825	0	0	0	0	0
AT1G43090	N/A				
AT3G11990	0	0	0	0	0
AT4G28310	0	0	0	7	0
AT1G53870	0	0	0	0	0
AT1G56000	0	0	0	0	0
AT1G58766	0	0	0	0	0
AT1G58725	0	0	0	0	0
AT1G20280	N/A				
AT1G61440	N/A				
AT1G62000	N/A				
AT1G68260	44	48	54	14	34
AT1G55860	86	99	22	0	15
AT2G16530	11	13	3	14	10
AT1G49715	0	0	0	0	0
AT1G74280	15	15	12	34	0
AT1G80980	0	0	2	9	17
AT2G06904	N/A				
AT5G04800	169	168	83	282	91
ATMG01300	N/A				
ATMG00540	N/A				
ATMG00560	0	0	0	0	0
ATMG00210	0	0	0	0	0
ATMG00220	0	0	0	0	0
ATMG00410	0	0	0	0	0
ATMG00900	0	0	0	0	0
ATMG00530	0	0	0	0	0
AT1G72510	N/A				
AT5G18380	3	24	1	43	2
AT4G02000	N/A				
AT4G35165	N/A				
AT3G44713	0	0	0	0	0

AT4G04030	0	0	0	0	0
AT2G20120	0	0	0	0	0
AT2G30910	67	37	21	44	52
AT2G43445	0	0	0	0	0
AT3G02242	N/A				
AT3G02610	0	0	0	0	0
AT3G05165	0	0	0	0	0
AT1G18330	0	0	0	0	0
AT3G17740	78	38	62	34	72
AT3G23530	2	164	168	16	82
AT3G55650	0	0	0	0	0
AT2G14282	N/A				
AT3G28290	0	0	0	0	0
AT5G62950	9	0	5	14	0
AT5G36150	0	0	0	0	0
AT3G29250	181	0	6	508	0
AT3G45710	0	7	2	70	2
AT3G47750	0	0	0	0	0
AT5G01430	0	0	0	0	0
AT5G01630	0	0	0	0	0
AT5G59390	0	0	0	0	0
AT4G10880	N/A				
AT2G05310	0	3	9	0	43
AT4G12620	22	24	2	10	3
AT4G15215	0	0	0	0	0
AT4G19750	N/A				
AT3G18240	0	0	0	0	0
AT4G23430	32	0	10	5	0
AT4G34080	0	0	0	0	0
AT4G34890	372	422	44	62	162
AT4G37680	0	0	10	0	0
AT1G01350	0	5	2	0	0
AT5G25757	80	76	95	192	50
AT5G28850	149	68	56	150	99
AT5G36740	0	0	0	0	0
AT5G36800	0	0	0	0	0
AT5G36810	N/A				
AT3G42565	N/A				
AT5G36662	N/A				
AT5G36690	0	0	0	0	0
AT5G37230	0	0	0	0	0
AT5G39200	0	0	0	0	0
AT5G39190	0	0	0	0	0
AT1G66500	268	6	6	4	50
AT5G50640	0	0	0	0	0
AT5G50700	0	0	0	0	0
AT2G07724	N/A				
AT2G07702	0	0	0	0	0
AT2G07714	N/A				

AT2G07722	0	0	0	0	0
AT2G07777	0	0	0	0	0
AT2G07702	0	0	0	0	0
AT2G07701	0	0	0	0	0

AP1	AP3	AGM	INS	ROS	SAP
9	5	0	4	43	0
0	0	0	6	0	0
0	7	0	3	0	0
0	0	0	0	0	0
0	0	0	0	0	0
4	12	15	7	4	31
0	0	0	0	0	0
0	0	0	0	0	0
0	0	0	0	0	0
0	0	0	0	0	0
0	0	0	0	0	0
0	0	0	0	0	0
0	0	0	0	0	0
4	6	0	3	0	0
3	7	0	0	15	0
0	0	0	0	0	0
0	0	0	0	0	0
0	0	0	0	0	0
0	0	0	0	0	0
4	14	8	12	0	2
38	45	113	74	31	30
5	8	0	1	63	0
0	0	0	0	0	0
11	2	0	0	5	0
11	27	4	13	11	3
486	497	300	433	844	405
0	0	0	0	0	0
0	0	0	0	0	0
0	0	0	0	0	0
0	0	0	0	0	0
0	0	0	0	0	0
0	0	0	0	0	0
56	29	15	42	23	5
0	0	0	0	0	0

0	0	0	0	0	0
0	0	0	0	0	0
23	45	23	10	21	40
0	0	0	0	0	4
0	0	0	0	0	0
0	0	0	0	0	0
0	0	0	0	0	0
6	18	16	8	5	12
0	4	0	4	0	0
0	0	0	0	0	0
0	0	0	0	0	0
0	7	6	1	0	8
0	0	0	0	0	0
2	0	0	0	132	0
0	0	0	4	38	3
0	0	0	0	0	0
0	1	3	0	0	0
0	0	0	0	0	0
32	3	0	8	0	1
0	5	0	0	0	4
17	7	9	9	17	26
0	0	0	0	0	0
0	0	0	0	0	0
3	12	0	5	43	5
0	2	0	0	0	0
27	32	26	30	44	50
0	0	8	0	0	0
0	0	0	0	0	4
80	14	44	28	98	104
52	37	32	27	55	0
0	0	0	0	0	0
0	0	0	0	0	0
0	0	0	0	0	0
0	0	0	0	0	0
0	0	0	0	0	0
0	0	0	0	0	0
0	8	0	10	0	0
0	0	0	0	0	0
0	0	0	0	0	0
0	0	0	0	0	0

0	0	0	0	0	0
0	0	0	0	0	0
0	0	0	0	0	0
0	0	0	0	0	0

S04	S52	LES	GSE	CAS	SIS
0	5	5	0	0	13
0	0	0	0	0	0
0	0	0	4	0	0
0	0	0	0	0	0
0	0	0	0	0	0
1	0	0	0	10	2
0	0	0	0	0	0
0	0	7	0	0	0
0	0	0	0	0	0
0	0	0	0	0	0
0	0	0	0	0	0
0	0	0	0	0	0
0	0	0	0	0	0
0	0	0	0	0	0
0	0	0	9	0	0
0	0	0	0	0	0
0	8	24	4	0	0
0	0	0	0	0	0
0	0	0	0	0	0
8	0	12	0	52	10
28	21	31	0	265	126
9	0	8	16	30	3
0	0	0	0	0	0
0	3	0	0	5	0
0	0	6	0	0	3
137	173	423	600	506	216
0	0	0	0	0	0
0	0	0	0	0	0
0	0	0	0	0	0
0	0	0	0	0	0
0	0	0	0	0	0
0	0	0	0	0	0
0	0	42	79	0	12
0	0	0	0	0	0

0	0	11	0	0	0
0	0	0	0	0	0
11	22	29	6	37	0
0	0	0	0	0	0

0	0	0	0	0	0
0	0	0	0	0	0
0	0	0	0	0	0
0	0	2	0	12	98
0	5	0	0	0	0
0	0	0	0	0	0

0	0	0	0	0	0
0	0	0	0	0	4
0	0	0	0	0	0
5	11	0	17	106	0
0	0	0	0	0	0
0	0	0	0	0	0
0	7	0	2	0	0
0	0	0	0	0	0
0	0	0	0	0	0

3	0	1	26	0	0
0	0	0	0	16	39
0	0	0	0	0	0

0	0	0	0	18	0
2	0	19	17	163	3
7	0	0	0	0	0
0	0	8	0	103	88
0	0	0	0	12	0
0	0	0	0	0	0
92	6	0	53	0	28
0	0	21	2	124	33
0	0	0	0	0	0
0	0	0	0	0	0

0	0	0	0	0	0
0	0	0	0	0	0
0	0	0	0	0	0
0	0	0	0	0	0
0	6	20	14	0	0
0	0	0	0	0	0
0	0	0	0	0	0

0	0	0	0	0	0

0	0	0	0	0	0
0	0	0	0	0	0
0	0	0	0	0	0
0	0	0	0	0	0
