# Supplementary material for: Divergent Evolutionary and Expression Patterns between Lineage Specific New Duplicate Genes and Their Parental Paralogs in Arabidopsis thaliana
Source: PLoS One. 2013 Aug 29;8(8):e72362. doi: 10.1371/journal.pone.0072362 (PMC3756979; doi:10.1371/journal.pone.0072362)
Supplement: Table S11 — Small RNA data of 100 new genes. (PDF) [file pone.0072362.s016.pdf]

Table S11 Small RNA data of 100 new genes

new_gene	FLR	RDR	SD1	SD2	SDC
AT1G14185	N/A				
AT1G19080	1	0	0	0	0
AT1G21530	N/A				
AT1G24880	81	3	0	0	0
AT1G25112	218	7	0	0	0
AT1G29410	0	0	97	203	153
AT1G29620	93	0	24	31	29
AT1G29830	23	0	10	7	9
AT1G30974	0	0	0	0	0
AT1G31670	N/A				
AT1G33607	32	0	0	0	0
AT1G34795	1	0	4	3	3
AT1G34820	1	0	4	3	3
AT1G34830	1	0	3	1	1
AT1G34850	1	0	3	1	1
AT1G34930	1	0	4	3	3
AT1G43100	13	0	2	3	3
AT1G45190	0	0	0	2	1
AT1G52270	N/A				
AT1G53890	22	0	0	0	0
AT1G55980	0	0	0	0	0
AT1G59077	12	0	0	0	0
AT1G59406	13	0	0	0	0
AT1G61200	7	0	0	36	18
AT1G61430	0	0	0	0	0
AT1G62080	0	0	0	0	0
AT1G68280	N/A				
AT1G70320	0	0	0	0	0
AT1G72590	N/A				
AT1G73607	N/A				
AT1G74290	17	0	0	0	0
AT1G80700	1	0	0	0	0
AT2G02840	9	45	0	1	1
AT2G04390	0	1	5	2	4
AT2G07692	0	0	0	0	0
AT2G07713	0	0	0	0	0
AT2G07715	5	2	0	0	0
AT2G07725	0	0	1	2	2
AT2G07727	7	0	0	0	0
AT2G07741	0	4	0	0	0
AT2G07771	6	0	1	0	1
AT2G07776	0	0	0	0	0
AT2G09970	0	0	0	0	0
AT2G09990	0	0	0	0	0
AT2G13450	20	0	2	3	3
AT2G14378	N/A				
AT2G14800	0	0	0	2	1

AT2G19850	24	0	10	9	10
AT2G20130	N/A				
AT2G31300	N/A				
AT2G43440	1	0	0	0	0
AT3G02240	N/A				
AT3G02620	N/A				
AT3G05160	N/A				
AT3G10113	N/A				
AT3G17712	N/A				
AT3G23510	3	0	0	0	0
AT3G25960	N/A				
AT3G27503	N/A				
AT3G28300	N/A				
AT3G28956	6	0	0	1	1
AT3G29255	1	0	0	0	0
AT3G29260	N/A				
AT3G45700	N/A				
AT3G47760	0	0	6	30	18
AT3G49420	0	0	0	0	0
AT4G00020	0	0	0	0	0
AT4G01180	0	0	0	0	0
AT4G10860	0	0	0	0	0
AT4G13500	0	0	0	0	0
AT4G14700	N/A				
AT4G15230	N/A				
AT4G19760	N/A				
AT4G21460	1	0	0	0	0
AT4G23420	0	16	15	19	17
AT4G33320	N/A				
AT4G34900	10	0	25	18	20
AT4G38320	N/A				
AT5G06420	N/A				
AT5G25754	0	0	2	0	1
AT5G28900	7	0	0	0	0
AT5G36670	0	0	0	0	0
AT5G36710	N/A				
AT5G36722	N/A				
AT5G36738	10	0	7	5	7
AT5G36739	3	0	4	5	5
AT5G36780	2	0	0	0	0
AT5G37270	0	0	0	0	0
AT5G39140	1	0	0	0	0
AT5G39160	1	0	0	0	0
AT5G43620	0	0	0	1	1
AT5G50530	0	0	0	1	1
AT5G50600	N/A				
ATMG00200	N/A				
ATMG00440	0	0	0	0	0
ATMG00550	0	0	0	0	0

ATMG00620	0	0	0	0	0
ATMG01090	6	1	0	0	0
ATMG01140	0	0	0	0	0
ATMG01150	0	0	0	0	0

AB1	AB2	ABC	FABAm	FABAp	FCol0
0	0	0	0	0	0
0	0	0	5	9	0
0	0	0	17	30	8
74	130	95	0	0	0
21	55	34	0	0	0
69	80	69	1	0	0
0	0	0	0	0	0
0	0	0	0	0	4
0	0	0	0	0	0
0	0	0	0	0	0
0	0	0	0	0	0
0	0	0	0	0	0
0	0	0	0	0	0
0	2	1	1	0	0
0	0	0	0	0	0
0	0	0	2	0	0
0	0	0	0	0	0
0	0	0	0	0	0
52	52	44	1	0	2
0	5	2	0	0	0
0	0	0	0	0	0
0	0	0	0	0	0
0	0	0	0	0	0
0	0	0	6	0	1
0	0	0	0	0	0
1	2	1	0	0	0
0	0	0	0	0	0
0	0	0	0	0	0
0	0	0	0	3	0
0	0	0	1	0	0
0	0	0	0	0	0
0	2	1	0	0	0
2	0	1	0	0	0
0	0	0	0	0	0
0	0	0	0	0	0
0	0	0	0	0	0
0	0	0	0	0	0
9	0	5	1	0	0
0	0	0	0	0	0

25	78	43	1	0	1
0	0	0	0	0	0
0	0	0	0	0	0
4	5	2	0	0	0
5	2	3	0	0	0
0	0	0	0	0	0
0	0	0	0	0	0
0	0	0	0	0	0
0	0	0	0	0	0
0	0	0	0	0	0
0	0	0	0	0	0
0	0	0	0	0	0
24	21	22	0	0	0
13	12	12	0	3	0
0	0	0	0	0	0
0	0	0	0	0	0
0	0	0	0	0	0
22	24	22	0	0	0
22	19	20	1	0	1
0	0	0	0	0	0
0	0	0	0	0	0
0	0	0	0	0	0
0	0	0	0	0	0
0	0	0	0	0	0
0	8	3	0	0	0
0	0	0	0	0	0
0	0	0	0	0	0

0	0	0	0	0	0
0	0	0	1	0	0
0	0	0	0	0	0
0	0	0	0	0	0

Fdcl17	Fdcl234	Frdr2	Frdr6	FBFL	FBLE
0	0	0	0	0	0
6	0	0	2	151	327
31	14	0	0	112	202
0	0	0	0	0	0
1	0	0	0	19	24
0	0	0	0	4	17
0	0	0	0	5	0
2	0	0	0	84	3
0	0	0	0	0	0
0	0	0	0	0	0
0	0	0	0	0	0
0	0	0	0	0	0
0	0	0	0	0	0
0	2	0	0	2	2
0	0	0	0	0	0
0	0	0	0	13	21
0	0	0	0	0	0
0	0	0	0	0	0
0	0	0	0	10	23
0	0	0	0	12	5
0	0	0	0	0	0
0	0	0	0	0	0
0	0	0	0	2	0
0	0	0	2	10	9
0	0	0	0	0	0
1	0	2	0	31	0
0	0	0	0	0	0
0	0	0	0	0	7
0	0	0	0	0	0
0	0	0	0	0	3
0	0	0	0	0	0
0	0	0	0	11	12
0	0	0	0	0	0
0	0	0	0	4	0
0	0	0	0	0	2
0	0	0	0	0	0
0	0	0	0	0	0
1	2	0	4	15	71
0	0	0	0	0	0

4	0	0	0	39	85
0	0	0	0	0	0
0	0	0	0	0	0
0	0	0	0	0	0
0	0	0	0	0	0
0	0	0	0	0	0
0	0	0	0	0	0
0	0	0	0	3	0
0	0	0	0	0	0
0	0	0	0	2	0
0	0	2	0	0	0
0	0	0	0	0	0
0	0	0	0	19	14
0	0	0	0	0	0
0	0	0	0	0	0
0	0	0	0	0	0
0	0	0	0	2	0
0	0	0	0	6	0
0	0	0	0	12	5
0	0	0	0	0	0
0	0	0	0	0	0
0	0	0	0	0	0
0	0	0	0	4	3
0	0	0	0	0	0
0	0	0	0	0	0
0	0	0	0	0	0
0	0	0	0	2	0

0	0	0	0	2	0
0	0	0	0	23	6
0	0	0	0	0	0
0	0	0	0	0	0

FBSE	FBSI	FHag1	FHag4	FHwe	FJWt
0	0	0	0	0	0
105	184	0	22	0	14
111	135	0	0	10	32
0	0	0	0	0	0
31	18	0	6	2	3
2	5	0	0	0	3
2	0	0	0	0	0
4	13	6	7	15	16
2	2	0	0	0	0
2	2	0	2	0	0
2	4	0	0	0	0
2	4	0	0	0	0
0	2	0	2	0	0
10	8	2	7	3	1
4	4	0	0	0	0
10	16	0	0	0	1
0	0	0	0	0	0
6	1	0	0	0	1
37	12	0	0	0	3
13	0	0	0	0	1
0	0	0	0	0	0
0	13	0	0	0	0
6	1	0	0	0	0
6	18	0	13	8	7
0	0	0	0	0	0
19	33	0	0	0	4
0	0	0	0	0	0
8	0	0	0	0	0
2	0	0	0	0	0
12	7	0	0	0	0
4	0	0	0	0	1
17	4	0	0	4	1
10	2	0	0	2	1
6	1	0	0	1	0
0	2	0	0	0	0
0	1	0	2	0	0
0	0	0	0	0	0
26	42	2	26	7	3
0	0	0	0	0	0

53	51	6	16	6	5
0	0	0	0	0	0
0	0	0	0	0	0
0	0	0	0	0	0
2	2	0	2	0	0
0	0	0	0	0	0
0	0	2	0	0	0
0	0	0	0	0	0
2	0	0	0	0	0
0	2	0	0	0	0
2	0	0	0	0	0
0	0	0	0	0	0
29	6	5	0	4	2
0	0	2	0	0	2
4	0	0	0	0	0
0	0	0	0	0	0
0	0	0	0	0	0
8	5	0	0	0	1
17	9	6	2	4	2
0	0	0	0	0	0
2	1	0	0	0	0
4	3	0	0	0	0
4	13	0	0	0	4
0	0	0	0	0	0
0	0	0	0	0	0
2	0	0	0	0	0
2	0	0	0	0	0

2	2	0	0	0	0
43	3	0	0	0	1
2	0	0	0	0	0
0	1	0	0	0	0

FJ1ab	FJ2ab	FJF1	JCWt	JC1hr	JC3hr
0	0	0	0	0	0
0	0	14	7	3	8
0	3	41	0	0	7
0	0	0	0	0	0
0	2	10	1	5	2
0	0	0	0	0	0
0	0	0	0	0	0
0	0	8	0	2	2
0	0	1	0	0	0
0	0	1	2	0	0
0	0	0	0	0	0
0	0	0	0	0	0
0	0	1	1	0	0
0	0	0	2	0	0
0	0	0	0	0	0
0	0	4	1	4	2
0	0	0	0	0	0
0	0	0	0	0	0
0	0	4	0	2	1
0	0	0	0	0	0
0	0	0	0	0	0
0	0	1	0	0	0
0	0	0	0	0	0
0	0	11	0	1	3
0	0	0	0	0	0
13	7	5	5	0	0
1	0	0	0	0	0
0	0	1	0	0	0
0	0	0	0	0	0
0	0	1	0	0	0
0	0	0	1	0	0
0	0	1	2	0	0
1	0	0	2	0	0
0	0	0	1	1	0
0	0	0	0	0	0
0	0	0	0	0	0
1	0	0	0	0	0
0	0	2	2	7	3
1	0	0	0	0	0

0	0	10	5	7	1
0	0	0	0	0	0
0	0	0	0	0	0
0	0	0	0	0	1
0	0	0	0	0	0
0	0	1	0	0	0
0	0	0	0	0	0
0	0	0	0	0	0
0	0	0	0	0	0
0	0	0	0	0	0
0	0	0	0	0	0
0	0	0	0	0	0
2	12	1	1	0	0
0	0	0	0	0	0
0	0	0	0	0	0
0	0	0	0	0	0
0	0	0	0	0	0
0	1	1	0	0	0
0	1	3	2	0	0
0	0	0	0	0	0
0	0	0	0	0	0
0	0	0	0	0	0
0	0	0	3	0	0
0	0	0	0	0	0
0	0	0	0	0	0
0	0	0	0	0	0
0	0	0	0	0	0

0	0	0	0	0	0
0	0	0	0	1	0
0	0	0	0	0	0
0	0	0	0	0	0

JCcmp	JCd1	JCd2	JCd3	JCd4	JCinf
0	0	0	0	0	0
11	11	5	2	8	29
24	0	0	0	1	15
0	0	0	0	0	0
2	0	1	1	4	8
1	0	2	0	2	1
0	0	0	0	0	0
10	7	17	11	14	51
1	0	0	0	1	3
0	0	1	0	0	2
1	0	0	0	1	3
1	0	0	0	1	3
0	0	0	0	0	1
0	0	0	0	0	4
0	0	0	0	0	0
0	5	4	2	4	4
0	0	1	0	0	0
0	0	0	0	0	2
0	1	1	2	0	3
0	1	0	0	0	0
0	0	0	0	0	0
0	0	0	0	0	0
0	0	1	0	0	2
4	2	0	1	0	2
0	0	0	0	0	0
5	0	0	0	0	3
0	0	0	0	0	0
0	0	0	0	0	1
0	0	0	0	0	2
1	0	0	0	0	0
0	0	0	0	0	0
1	0	1	1	0	3
1	1	1	0	1	0
0	0	0	0	0	0
0	0	0	0	0	0
0	0	0	0	0	0
0	0	0	0	0	0
3	1	0	3	5	5
0	0	0	0	0	0

4	4	1	2	3	10
0	0	0	0	0	0
0	0	0	0	0	0
0	0	0	0	0	0
0	0	0	0	0	0
0	0	0	0	0	0
0	0	0	0	0	0
0	0	0	0	0	0
0	0	0	0	0	0
0	0	0	0	0	0
0	0	0	0	0	0
0	0	0	0	0	0
7	1	0	0	0	1
0	0	0	0	0	0
0	0	0	0	0	1
1	0	0	0	0	0
0	0	0	0	0	0
1	0	0	0	0	3
0	0	0	0	1	4
0	0	0	0	0	0
0	0	0	0	0	0
1	0	0	0	0	0
2	1	0	0	0	0
0	0	0	0	0	0
0	0	0	0	0	0
0	0	0	0	0	0
0	0	0	0	0	0

0	0	0	0	0	0
1	0	0	0	0	0
0	0	0	0	0	0
0	0	1	0	0	0

JClef	JCr1	JCr2	JCr6a	JCr6b
0	0	0	0	0
21	4	1	7	15
0	0	0	10	1
0	0	0	0	0
1	3	0	2	2
0	0	0	1	0
0	0	0	0	0
0	18	3	1	21
0	0	0	0	1
0	0	0	0	1
0	0	0	0	1
0	0	0	0	1
0	0	0	0	1
0	1	0	1	0
0	0	0	0	0
3	6	0	1	4
0	0	0	0	0
0	1	0	0	1
0	1	0	3	1
1	0	0	1	0
0	0	1	0	0
0	0	0	0	0
0	2	0	0	0
0	0	0	2	2
0	0	0	0	0
0	0	3	1	0
0	0	1	0	0
0	0	0	1	0
0	0	0	1	0
0	1	1	0	1
0	0	0	1	1
0	2	0	0	0
0	0	0	0	1
0	0	0	2	0
0	0	0	0	0
0	0	0	0	0
0	0	0	0	1
2	0	0	0	5
0	0	0	0	0

2	3	1	3	6
0	0	0	0	0
0	0	0	0	0
0	0	0	0	0
0	0	0	2	0
0	0	0	0	0
0	0	0	0	0
0	0	0	0	0
0	0	0	0	0
0	0	0	0	0
0	0	0	0	0
0	0	0	0	0
1	0	4	0	0
0	0	0	0	0
0	0	0	0	0
0	0	0	0	0
0	0	0	0	0
0	0	0	0	0
0	1	0	1	0
0	0	0	0	0
0	0	0	0	0
0	0	0	0	0
0	1	0	1	1
0	0	0	0	0
0	0	0	0	0
0	0	0	0	0
0	0	0	0	0

0	0	0	1	0
0	1	0	1	0
0	0	0	0	0
0	0	0	0	0
