# Supplementary material for: Divergent Evolutionary and Expression Patterns between Lineage Specific New Duplicate Genes and Their Parental Paralogs in Arabidopsis thaliana
Source: PLoS One. 2013 Aug 29;8(8):e72362. doi: 10.1371/journal.pone.0072362 (PMC3756979; doi:10.1371/journal.pone.0072362)
Supplement: Table S12 — Small RNA data of 100 old genes. (PDF) [file pone.0072362.s017.pdf]

Table S12 Small RNA data of 100 old genes

parental_gene	FLR	RDR	SD1	SD2	SDC
AT1G14190	N/A				
AT3G55490	1	0	0	0	0
AT1G21540	N/A				
AT1G25054	81	3	0	0	0
AT1G25025	218	7	0	0	0
AT1G07780	0	0	235	417	329
AT1G32720	79	0	22	33	30
AT1G29820	N/A				
AT1G30972	0	0	0	0	0
AT1G31690	0	0	0	0	0
AT5G08055	21	0	0	0	0
AT1G34815	1	0	3	1	1
AT1G34825	1	0	4	3	3
AT1G34815	1	0	3	1	1
AT1G34840	1	0	3	1	1
AT1G34825	1	0	4	3	3
AT1G43090	13	0	4	5	5
AT3G11990	0	0	0	0	0
AT4G28310	N/A				
AT1G53870	21	0	0	0	0
AT1G56000	0	0	0	0	0
AT1G58766	12	0	0	0	0
AT1G58725	13	0	0	0	0
AT1G20280	6	0	0	37	19
AT1G61440	N/A				
AT1G62000	0	0	0	0	0
AT1G68260	0	0	0	0	0
AT1G55860	0	0	0	0	0
AT2G16530	0	0	0	0	0
AT1G49715	N/A				
AT1G74280	N/A				
AT1G80980	N/A				
AT2G06904	18	11	12	0	6
AT5G04800	2	0	0	0	0
ATMG01300	0	0	0	0	0
ATMG00540	0	0	0	0	0
ATMG00560	15	5	0	0	0
ATMG00210	0	0	0	0	0
ATMG00220	7	0	0	0	0
ATMG00410	0	4	0	0	0
ATMG00900	6	0	1	0	1
ATMG00530	0	0	0	0	0
AT1G72510	N/A				
AT5G18380	0	0	0	0	0
AT4G02000	8	0	2	3	3
AT4G35165	N/A				

AT3G44713	N/A				
AT4G04030	19	8	13	10	13
AT2G20120	N/A				
AT2G30910	0	4	0	0	0
AT2G43445	1	0	0	0	0
AT3G02242	N/A				
AT3G02610	23	0	71	82	78
AT3G05165	0	0	0	0	0
AT1G18330	N/A				
AT3G17740	9	0	0	0	0
AT3G23530	0	4	0	0	0
AT3G55650	0	0	0	0	0
AT2G14282	N/A				
AT3G28290	N/A				
AT5G62950	3	0	0	0	0
AT5G36150	36	4	89	120	104
AT3G29250	0	0	12	19	16
AT3G45710	9	1	0	0	0
AT3G47750	N/A				
AT5G01430	0	14	0	0	0
AT5G01630	0	0	1	1	1
AT5G59390	N/A				
AT4G10880	0	0	0	0	0
AT2G05310	0	0	0	0	0
AT4G12620	N/A				
AT4G15215	2	0	0	0	0
AT4G19750	N/A				
AT3G18240	0	0	0	0	0
AT4G23430	0	0	0	0	0
AT4G34080	N/A				
AT4G34890	11	0	25	18	20
AT4G37680	0	0	0	0	0
AT1G01350	N/A				
AT5G25757	0	0	2	0	1
AT5G28850	0	0	0	0	0
AT5G36740	0	0	0	0	0
AT5G36800	N/A				
AT5G36810	N/A				
AT3G42565	10	0	22	25	25
AT5G36662	3	0	4	5	5
AT5G36690	2	0	0	0	0
AT5G37230	N/A				
AT5G39200	1	0	0	0	0
AT5G39190	1	0	0	0	0
AT1G66500	0	0	0	1	1
AT5G50640	0	0	0	1	1
AT5G50700	N/A				
AT2G07724	N/A				
AT2G07702	0	0	0	0	0

AT2G07714	0	0	0	0	0
AT2G07722	0	0	0	0	0
AT2G07777	6	1	0	0	0
AT2G07702	0	0	0	0	0
AT2G07701	0	0	0	0	0

AB1	AB2	ABC	FABAm	FABAp	FCol0
0	0	0	0	0	0
0	0	0	5	9	0
0	0	0	17	30	8
410	580	467	0	0	0
44	83	51	0	0	0
0	0	0	0	0	0
0	0	0	0	0	0
0	0	0	1	0	0
0	0	0	0	0	0
0	0	0	0	0	0
0	0	0	0	0	0
0	0	0	0	0	0
0	0	0	0	0	0
0	7	3	1	0	0
0	0	0	0	0	0
0	0	0	2	0	0
0	0	0	0	0	0
0	0	0	0	0	0
52	52	44	1	0	2
0	5	2	0	0	0
0	0	0	0	0	0
0	2	1	0	0	0
0	0	0	0	0	0
0	0	0	0	0	0
1	5	3	0	0	0
0	0	0	0	0	0
0	0	0	0	0	0
0	0	0	0	3	0
0	2	1	1	0	0
0	0	0	0	0	0
0	2	1	0	0	0
2	0	1	0	0	0
0	0	0	0	0	0
0	0	0	0	0	0
0	0	0	0	0	0
11	0	6	1	0	0

40	64	45	1	0	1
0	0	0	0	0	0
0	0	0	0	0	0
65	61	65	1	0	3
0	0	0	0	0	0
0	0	0	0	0	0
0	0	0	0	0	0
0	0	0	0	0	0
0	0	0	0	0	0
144	124	137	0	3	1
0	0	0	0	0	0
0	0	0	0	0	0
0	0	0	0	0	0
0	0	0	0	0	0
1	2	1	0	0	0
0	0	0	0	0	0
0	0	0	0	0	0
0	0	0	0	0	0
0	0	0	0	0	0
13	12	12	0	3	0
0	0	0	0	0	0
0	0	0	0	0	0
0	0	0	0	0	0
0	0	0	0	0	0
23	22	22	1	0	1
22	19	20	1	0	1
0	0	0	0	0	0
0	0	0	0	0	0
0	0	0	0	0	0
0	0	0	0	0	0
0	8	3	0	0	0
0	0	0	0	0	0

0	0	0	0	0	0
0	0	0	0	0	0
0	0	0	1	0	0
0	0	0	0	0	0
0	0	0	0	0	0

Fdcl17	Fdcl234	Frdr2	Frdr6	FBFL	FBLE
0	0	0	0	0	0
6	0	0	2	151	327
31	14	0	0	112	202
0	0	0	0	0	0
3	0	0	4	21	34
0	0	0	0	8	4
0	0	0	0	0	0
1	0	0	0	26	11
0	0	0	0	0	0
0	0	0	0	0	0
0	0	0	0	0	0
0	0	0	0	0	0
0	0	0	0	0	0
0	2	0	0	4	4
0	0	0	0	3	0
0	0	0	0	13	21
0	0	0	0	0	2
0	0	0	0	0	0
0	0	0	0	10	23
0	0	0	0	12	2
0	0	0	0	0	0
0	0	0	0	0	0
0	0	0	0	2	0
0	0	0	0	0	0
0	0	0	0	9	2
0	0	0	0	0	0
0	0	0	0	0	7
0	0	0	0	0	0
0	0	0	4	2	5
0	0	0	0	0	0
0	0	0	0	8	5
0	0	0	0	0	0
0	0	0	0	4	0
0	0	0	0	0	2
0	0	0	0	2	0
0	2	0	0	6	20

2	0	0	2	54	103
0	0	0	0	4	0
0	0	0	0	0	0
0	0	0	4	9	4
0	0	0	0	0	0
0	0	0	0	7	0
0	0	0	0	0	0
0	0	0	0	0	0
0	0	0	0	5	0
2	2	0	0	20	2
0	0	0	0	0	0
0	0	0	0	0	0
0	0	0	0	0	0
0	0	0	0	0	0
0	0	0	0	2	0
0	0	0	0	0	0
0	0	0	0	0	0
0	0	0	0	3	0
0	0	0	0	0	0
0	0	0	0	0	0
0	0	0	0	2	0
0	0	0	0	0	0
0	0	0	0	0	0
0	0	0	0	2	0
0	0	0	0	20	5
0	0	0	0	12	5
0	0	0	0	0	0
0	0	0	0	0	0
0	0	0	0	9	3
0	0	0	0	0	0
0	0	0	0	0	0
0	0	0	0	0	0

0	0	0	0	2	0
0	0	0	0	2	0
0	0	0	0	23	6
0	0	0	0	0	0
0	0	0	0	0	0

FBSE	FBSI	FHag1	FHag4	FHwe	FJWt
0	0	0	0	0	0
105	184	0	22	0	14
111	135	0	0	10	32
0	0	0	0	0	0
31	37	0	6	2	3
0	2	0	0	0	4
2	0	0	0	0	0
10	18	2	9	6	2
2	4	0	0	0	0
2	2	0	2	0	0
2	4	0	0	0	0
2	4	0	0	0	0
2	2	0	2	0	0
19	8	2	7	4	0
10	4	0	0	0	0
6	16	0	0	0	0
0	0	0	0	0	0
6	1	0	0	0	1
37	12	0	0	0	3
13	2	0	0	0	0
0	14	0	0	0	0
0	0	0	0	0	0
6	4	0	0	0	0
0	0	0	0	1	0
10	5	0	0	0	0
0	1	0	0	0	0
2	0	0	0	0	0
2	0	0	0	0	0
24	8	0	0	1	0
2	0	0	0	0	1
17	4	0	0	4	1
10	3	0	0	2	1
6	1	0	0	1	0
0	2	0	0	0	0
2	0	0	0	0	0
10	8	0	4	0	0

64	66	10	20	8	7
0	1	0	0	0	0
0	0	0	0	0	0
4	11	0	5	5	3
2	0	0	0	0	0
0	7	0	0	0	0
0	0	0	0	0	0
2	0	0	0	0	0
0	0	0	0	0	0
10	12	0	4	0	5
0	0	0	0	0	0
2	0	0	0	0	0
0	0	0	0	0	0
0	0	0	0	0	0
5	1	0	0	0	0
0	0	0	0	0	0
0	0	0	0	0	0
0	0	0	0	0	0
5	0	0	0	0	0
0	0	2	0	0	2
0	0	0	0	0	0
6	0	0	0	0	0
0	0	0	0	0	0
0	0	0	0	0	0
20	11	6	11	5	2
17	9	6	2	4	2
0	0	0	0	0	0
4	3	0	0	0	0
4	13	0	0	0	4
0	0	0	0	0	0
0	0	0	0	0	0
2	0	0	0	0	0

2	1	0	0	0	0
2	2	0	0	0	0
43	4	0	0	0	1
2	0	0	0	0	0
0	1	0	0	0	0

FJ1ab	FJ2ab	FJF1	JCWt	JC1hr	JC3hr
0	0	0	0	0	0
0	0	14	7	3	8
0	3	41	0	0	7
0	0	0	0	0	0
0	1	10	0	4	2
0	0	1	1	0	0
0	0	0	0	0	0
0	0	1	0	6	4
0	0	0	0	0	0
0	0	1	2	0	0
0	0	0	0	0	0
0	0	0	0	0	0
0	0	1	2	0	0
0	0	0	3	0	0
0	0	0	0	1	0
0	0	4	1	4	2
0	0	0	0	0	0
0	0	0	0	0	0
0	0	4	0	2	1
0	0	0	0	1	0
0	0	1	0	0	0
0	0	0	0	0	0
0	0	0	0	0	0
0	0	0	0	0	0
5	2	4	0	0	0
0	0	0	0	0	0
0	0	0	0	0	0
0	0	0	0	0	0
1	0	0	3	0	0
0	0	0	0	0	0
0	0	1	1	0	0
1	0	0	2	0	0
0	0	0	1	1	0
0	0	0	0	0	0
1	0	0	0	0	0
0	0	2	1	2	1

0	0	11	6	7	1
0	0	0	0	0	0
0	0	0	0	0	0
0	0	0	1	1	0
0	0	0	0	0	0
0	0	0	0	0	0
0	0	0	0	0	0
0	0	0	0	0	0
0	0	0	0	0	0
0	0	3	1	1	0
0	0	0	0	0	0
0	0	0	0	0	0
0	0	0	0	0	0
0	0	0	0	0	0
0	0	0	0	0	0
0	0	0	0	0	0
0	0	0	0	0	0
0	0	0	0	0	0
0	0	0	0	0	0
0	0	0	0	0	0
0	0	0	0	0	0
0	0	0	0	0	0
0	0	0	0	0	0
0	0	0	0	0	0
0	0	5	1	2	1
0	1	3	2	0	0
0	0	0	0	0	0
0	0	0	0	0	0
0	0	0	3	0	0
0	0	0	0	0	0
0	0	0	0	0	0
0	0	0	0	0	0

0	0	0	1	0	0
0	0	0	0	0	0
0	0	0	0	1	0
0	0	0	0	0	0
0	0	0	0	0	0

JCcmp	JCd1	JCd2	JCd3	JCd4	JCinf
0	0	0	0	0	0
11	11	5	2	8	29
24	0	0	0	1	15
0	0	0	0	0	0
2	1	0	0	3	11
0	1	0	0	3	2
0	0	0	0	0	0
6	7	5	6	5	12
1	0	0	0	1	3
0	0	1	0	0	2
1	0	0	0	1	3
1	0	0	0	1	3
0	0	1	0	0	2
0	0	2	0	0	2
0	0	0	0	0	0
0	5	4	2	4	4
0	0	1	0	0	0
0	0	0	0	0	2
0	1	1	2	0	3
1	0	0	0	0	2
0	0	0	0	0	0
0	0	0	0	0	0
0	0	1	0	0	1
0	0	0	0	0	0
0	0	0	0	0	0
0	0	0	0	0	0
0	0	0	0	0	0
0	0	0	0	0	2
1	0	0	0	0	0
0	0	0	0	0	0
1	0	1	1	0	3
1	1	1	0	2	0
0	0	0	0	0	0
0	0	0	0	0	0
0	0	0	0	0	0
1	1	0	3	3	2

4	3	1	3	4	11
0	0	0	0	0	0
0	0	0	0	0	0
1	0	0	0	0	2
0	0	0	0	0	0
0	0	0	0	0	0
0	0	0	0	0	0
0	0	0	0	0	0
0	0	0	0	0	0
1	1	1	1	2	2
0	0	0	0	0	0
0	0	0	0	0	0
0	0	0	0	0	0
0	0	0	0	0	0
1	0	0	0	0	0
0	0	0	0	0	0
0	0	0	0	0	0
0	0	0	0	0	0
0	0	0	0	0	0
0	0	0	0	0	0
0	0	0	0	0	0
0	0	0	0	0	1
1	0	0	0	0	0
0	0	0	0	0	0
0	1	0	4	2	6
0	0	0	0	1	4
0	0	0	0	0	0
1	0	0	0	0	0
2	1	0	0	0	0
0	0	0	0	0	0
0	0	0	0	0	0
0	0	0	0	0	0

0	0	0	0	0	1
0	0	0	0	0	0
1	0	0	0	0	0
0	0	0	0	0	0
0	0	1	0	0	0

JClef	JCr1	JCr2	JCr6a	JCr6b
0	0	0	0	0
21	4	1	7	15
0	0	0	10	1
0	0	0	0	0
1	4	0	3	2
0	0	0	0	0
0	0	0	0	0
0	5	1	2	5
0	0	0	0	1
0	0	0	0	1
0	0	0	0	1
0	0	0	0	1
0	0	0	0	1
0	1	0	2	0
0	0	0	0	0
3	6	0	1	2
0	0	0	0	0
0	1	0	0	1
0	1	0	3	1
1	0	0	1	0
0	0	0	0	0
0	0	0	0	0
0	2	0	0	0
0	0	0	0	0
0	0	0	0	0
0	0	0	0	0
0	0	0	0	0
0	0	0	1	0
0	1	1	0	4
0	0	0	0	0
0	2	0	0	0
0	0	0	1	1
0	0	0	2	0
0	0	0	0	0
0	0	0	0	1
2	0	0	0	1

4	4	1	4	6
0	0	0	0	0
0	0	0	0	0
0	1	0	0	1
0	0	0	0	0
0	0	0	0	0
0	0	0	0	0
0	0	0	0	0
0	0	0	0	0
2	1	0	2	1
0	0	0	0	0
0	0	0	0	0
0	0	0	0	0
0	0	0	0	0
0	0	0	1	1
0	0	0	1	0
0	0	0	0	1
0	0	0	0	0
0	0	0	0	0
0	0	0	0	0
0	0	0	0	0
0	0	0	0	0
0	0	0	0	0
0	0	0	0	0
0	2	1	3	2
0	1	0	1	0
0	0	0	0	0
0	0	0	0	0
0	1	0	1	1
0	0	0	0	0
0	0	0	0	0
0	0	0	0	0

0	0	0	0	0
0	0	0	1	0
0	1	0	1	0
0	0	0	0	0
0	0	0	0	0
