# Supplementary material for: Divergent Evolutionary and Expression Patterns between Lineage Specific New Duplicate Genes and Their Parental Paralogs in Arabidopsis thaliana
Source: PLoS One. 2013 Aug 29;8(8):e72362. doi: 10.1371/journal.pone.0072362 (PMC3756979; doi:10.1371/journal.pone.0072362)
Supplement: Table S13 — RNA-seq data for 100 duplicated gene pairs. (PDF) [file pone.0072362.s018.pdf]

Table S13 RNA-seq data for 100 duplicated gene pairs

new_gene	old_gene	new_seedling:root:fbud	old_seedling:root:fbud
AT1G14185	AT1G14190	1.31155:25.4665:1.22169	7.72487:20.3881:3.9649
AT1G19080	AT3G55490	10.0137:9.20446:10.3212	5.3992:9.81773:5.09297
AT1G21530	AT1G21540	0:0.126146:0.373556	10.3067:0.111466:33.9706
AT1G24880	AT1G25054	140.328:113.533:137.326	17.299:19.3308:25.322
AT1G25112	AT1G25025	0.500353:0:0	0
AT1G29410	AT1G07780	13.7979:8.77877:11.2621	35.2196:32.1865:33.4348
AT1G29620	AT1G32720	0	0
AT1G29830	AT1G29820	0.943935:3.59975:3.07213	17.4528:30.7282:20.8067
AT1G30974	AT1G30972	0	8.64583E-06
AT1G31670	AT1G31690	0.0915719:0:0	41.2126:0:0
AT1G33607	AT5G08055	0	3.67291:0:0
AT1G34795	AT1G34815	0	0
AT1G34820	AT1G34825	0	0
AT1G34830	AT1G34815	0	0
AT1G34850	AT1G34840	0	0
AT1G34930	AT1G34825	0	0
AT1G43100	AT1G43090	0	0
AT1G45190	AT3G11990	3.54745E-05	8.8588E-05
AT1G52270	AT4G28310	22.6755:4.65394:15.5729	122.35:79.6843:92.6394
AT1G53890	AT1G53870	32.9633:0:6.90001	31.9949:0:3.65719
AT1G55980	AT1G56000	2.54233:1.14527:3.6418	19.308:11.9617:17.5269
AT1G59077	AT1G58766	1.05324E-06	1.05324E-06
AT1G59406	AT1G58725	0.430692:0:0.187924	0.430692:0:0.375848
AT1G61200	AT1G20280	0.000155903	0.000318461
AT1G61430	AT1G61440	7.30392:6.35831:5.02934	0.0953651:1.09461:0.323736
AT1G62080	AT1G62000	0	5.27778E-06
AT1G68280	AT1G68260	0	14.3549:7.27507:12.3209
AT1G70320	AT1G55860	50.4595:50.8233:40.5112	33.6434:31.526:27.9259
AT1G72590	AT2G16530	7.605134919	5.46488:22.8418:5.44571
AT1G73607	AT1G49715	0	1.49537E-05
AT1G74290	AT1G74280	6.90159:3.84949:4.56161	14.6881:34.8002:13.1726
AT1G80700	AT1G80980	41.1044:39.4574:39.0612	15.559:15.3993:17.3664
AT2G02840	AT2G06904	0:0.130136:0.467187	0
AT2G04390	AT5G04800	227.833:193.609:173.357	141.278:146.975:110.943
AT2G07692	ATMG01300	1.91991:1.10013:2.14154	10.28548483
AT2G07713	ATMG00540	0	1.69097E-05
AT2G07715	ATMG00560	8.9127:8.13294:10.1929	6.35144:4.07579:11.7896
AT2G07725	ATMG00210	6.91822:6.56375:7.55168	8.23121:7.48048:7.44845
AT2G07727	ATMG00220	5.63646:8.1358:6.2809	2.81721:5.39516:3.95378
AT2G07741	ATMG00410	5.41557:7.14984:8.21016	5.8401:6.26225:8.06663
AT2G07771	ATMG00900	0.662592:0.590027:0	0.662592:0.88504:0
AT2G07776	ATMG00530	2.34586:1.5739:1.81905	0:1.83519:1.90532
AT2G09970	AT1G72510	0:0.308211:0.298332	51.7221:82.6068:48.9175
AT2G09990	AT5G18380	162.965:146.37:128.971	282.382:237.23:223.161
AT2G13450	AT4G02000	1.548369606	0.902292:0.769864:2.71301

AT2G14378	AT4G35165	0.000125787	0.314803:0.267748:2.5347 1
AT2G14800	AT3G44713	6.85921:3.38482:6.16844	6.50175:6.47596:13.3976
AT2G19850	AT4G04030	0:0.476181:1.85379	0
AT2G20130	AT2G20120	13.5383:24.4345:16.8999	24.0812:23.0846:28.9644
AT2G31300	AT2G30910	5.20241:8.6094:6.79791	7.67485:10.1149:8.39836
AT2G43440	AT2G43445	4.00306:0.156947:1.84799	4.81662:5.8327:3.35552
AT3G02240	AT3G02242	0.073916725	0.068958993
AT3G02620	AT3G02610	1.24716:21.5884:3.10561	1.12994:11.0004:8.56174
AT3G05160	AT3G05165	8.77574:3.66629:4.6501	19.7187:41.6861:19.2937
AT3G10113	AT1G18330	21.8897:7.46883:12.1323	20.9588:9.25265:11.9538
AT3G17712	AT3G17740	18.4673:21.5861:18.3758	17.2797:16.8404:16.1658
AT3G23510	AT3G23530	3.55728:27.744:1.93132	33.1458:35.9188:23.1907
AT3G25960	AT3G55650	0	0.49609:0:0.525782
AT3G27503	AT2G14282	0.58514:0:52.6936	0.001743449
AT3G28300	AT3G28290	22.492:8.21997:11.3556	22.492:7.77161:13.1486
AT3G28956	AT5G62950	12.8838:14.2345:11.3966	21.8006:36.0892:29.1516
AT3G29255	AT5G36150	2.2037E-05	0:4.33037:1.21777
AT3G29260	AT3G29250	0.620388137	1.62573:177.7:1.18703
AT3G45700	AT3G45710	0.2594:17.5317:0.106694	1.49817:79.1155:1.52971
AT3G47760	AT3G47750	0.15143669	4.65453:0.128893:2.87778
AT3G49420	AT5G01430	22.3663:25.3407:23.0569	44.1366:44.8056:44.7055
AT4G00020	AT5G01630	10.9303:8.35818:7.06397	3.5916:4.3479:4.15706
AT4G01180	AT5G59390	0.170831:0.150905:0	0.53854:0.454873:30.0516
AT4G10860	AT4G10880	0.539944:4.42007:0	0
AT4G13500	AT2G05310	95.3573:0.733095:45.246	124.092:5.31995:70.5243
AT4G14700	AT4G12620	5.1261:7.60494:12.9562	31.017:31.0413:21.0543
AT4G15230	AT4G15215	0.565947:14.3115:0.438504	1.64705:3.06071:3.00909
AT4G19760	AT4G19750	0	0
AT4G21460	AT3G18240	56.0093:49.9803:48.2605	68.6465:78.7351:63.878
AT4G23420	AT4G23430	3.82178:32.2568:3.69432	29.7369:58.8478:16.973
AT4G33320	AT4G34080	3.18677:2.10792:5.2933	5.89071e-266:4.61388:6.12154e-212
AT4G34900	AT4G34890	17.4873:1.81676:7.86386	26.9607:47.2803:30.267
AT4G38320	AT4G37680	15.611:24.2674:22.9355	34.2159:41.8352:47.971
AT5G06420	AT1G01350	16.4678:27.0054:18.7697	55.3847:64.1393:58.3855
AT5G25754	AT5G25757	51.741:54.6304:36.0351	30.8496:34.2244:19.3257
AT5G28900	AT5G28850	28.0545:23.3891:22.9313	16.2165:18.2507:15.31
AT5G36670	AT5G36740	7.34158:8.43149:13.6018	8.13947:6.71067:14.0668
AT5G36710	AT5G36800	30.0824:25.1761:22.0342	32.8451:24.6726:18.9869
AT5G36722	AT5G36810	0	0
AT5G36738	AT3G42565	4.47106E-05	1.44907E-05
AT5G36739	AT5G36662	2.97569E-05	4.46412E-05
AT5G36780	AT5G36690	1.5162E-06	1.5162E-06
AT5G37270	AT5G37230	0	4.262457963
AT5G39140	AT5G39200	0	0
AT5G39160	AT5G39190	2.10002:5.75315:5.98736	2.09231:8.60044:6.22808
AT5G43620	AT1G66500	8.87914:8.71858:11.1073	17.5519:16.6642:22.3314
AT5G50530	AT5G50640	12.6237:9.72921:15.2125	13.8638:10.4498:12.6408

AT5G50600	AT5G50700	0:0.340857:0.331868	0:0.668587:0.651513
ATMG00200	AT2G07724	1.97801E-05	2.07307:1.70227:1.68964
ATMG00440	AT2G07702	0.865083:0:0.725083	0.865083:0:1.45017
ATMG00550	AT2G07714	0	0.518655:0:0
ATMG00620	AT2G07722	13.8522:10.9861:13.9223	15.1445:13.1134:11.5222
ATMG01090	AT2G07777	7.6104:6.56382:5.65338	3.42652:2.18712:2.08371
ATMG01140	AT2G07702	0	0.865083:0:1.45017
ATMG01150	AT2G07701	3.78486:0:1.59128	1.89243:1.63739:0
