# Supplementary material for: Divergent Evolutionary and Expression Patterns between Lineage Specific New Duplicate Genes and Their Parental Paralogs in Arabidopsis thaliana
Source: PLoS One. 2013 Aug 29;8(8):e72362. doi: 10.1371/journal.pone.0072362 (PMC3756979; doi:10.1371/journal.pone.0072362)
Supplement: Table S14 — Gene expression sources of 100 new genes. (PDF) [file pone.0072362.s019.pdf]

Table S14 Gene expression sources of 100 new genes

new gene	est	unigene	mpss	srna	tiling array	rna seq
AT1G14185	est:+	unigene:+	mpss:	srna:	tiling:+	rna_seq:+
AT1G19080	est:+	unigene:	mpss:+	srna:+	tiling:+	rna_seq:+
AT1G21530	est:	unigene:+	mpss:+	srna:	tiling:+	rna_seq:+
AT1G24880	est:+	unigene:+	mpss:	srna:+	tiling:	rna_seq:+
AT1G25112	est:	unigene:	mpss:	srna:+	tiling:	rna_seq:+
AT1G29410	est:+	unigene:	mpss:+	srna:+	tiling:+	rna_seq:+
AT1G29620	est:	unigene:+	mpss:	srna:+	tiling:	rna_seq:
AT1G29830	est:	unigene:+	mpss:+	srna:+	tiling:+	rna_seq:+
AT1G30974	est:	unigene:+	mpss:+	srna:+	tiling:+	rna_seq:
AT1G31670	est:+	unigene:+	mpss:	srna:	tiling:+	rna_seq:+
AT1G33607	est:	unigene:+	mpss:	srna:+	tiling:+	rna_seq:
AT1G34795	est:	unigene:+	mpss:	srna:+	tiling:	rna_seq:
AT1G34820	est:	unigene:	mpss:	srna:+	tiling:	rna_seq:
AT1G34830	est:	unigene:+	mpss:	srna:+	tiling:	rna_seq:
AT1G34850	est:	unigene:+	mpss:	srna:+	tiling:	rna_seq:
AT1G34930	est:	unigene:+	mpss:	srna:+	tiling:+	rna_seq:
AT1G43100	est:	unigene:+	mpss:	srna:+	tiling:+	rna_seq:
AT1G45190	est:+	unigene:	mpss:	srna:+	tiling:+	rna_seq:+
AT1G52270	est:	unigene:+	mpss:+	srna:	tiling:+	rna_seq:+
AT1G53890	est:	unigene:+	mpss:	srna:+	tiling:	rna_seq:+
AT1G55980	est:	unigene:+	mpss:	srna:+	tiling:+	rna_seq:+
AT1G59077	est:	unigene:	mpss:	srna:+	tiling:	rna_seq:+
AT1G59406	est:	unigene:+	mpss:	srna:+	tiling:	rna_seq:+
AT1G61200	est:	unigene:	mpss:	srna:+	tiling:+	rna_seq:+
AT1G61430	est:	unigene:+	mpss:+	srna:+	tiling:+	rna_seq:+
AT1G62080	est:+	unigene:+	mpss:	srna:+	tiling:+	rna_seq:
AT1G68280	est:	unigene:+	mpss:	srna:	tiling:+	rna_seq:
AT1G70320	est:+	unigene:	mpss:+	srna:+	tiling:+	rna_seq:+
AT1G72590	est:+	unigene:+	mpss:	srna:	tiling:+	rna_seq:+
AT1G73607	est:+	unigene:	mpss:	srna:	tiling:	rna_seq:
AT1G74290	est:	unigene:+	mpss:+	srna:+	tiling:+	rna_seq:+
AT1G80700	est:+	unigene:+	mpss:+	srna:+	tiling:+	rna_seq:+
AT2G02840	est:	unigene:+	mpss:+	srna:+	tiling:+	rna_seq:+
AT2G04390	est:+	unigene:+	mpss:	srna:+	tiling:+	rna_seq:+
AT2G07692	est:	unigene:+	mpss:	srna:+	tiling:	rna_seq:+
AT2G07713	est:	unigene:	mpss:	srna:+	tiling:	rna_seq:
AT2G07715	est:+	unigene:+	mpss:	srna:+	tiling:	rna_seq:+
AT2G07725	est:	unigene:+	mpss:	srna:+	tiling:	rna_seq:+
AT2G07727	est:	unigene:+	mpss:	srna:+	tiling:	rna_seq:+
AT2G07741	est:	unigene:+	mpss:	srna:+	tiling:	rna_seq:+
AT2G07771	est:	unigene:	mpss:	srna:+	tiling:	rna_seq:+
AT2G07776	est:	unigene:+	mpss:	srna:+	tiling:	rna_seq:+
AT2G09970	est:	unigene:+	mpss:	srna:+	tiling:+	rna_seq:+
AT2G09990	est:+	unigene:+	mpss:+	srna:+	tiling:+	rna_seq:+
AT2G13450	est:	unigene:+	mpss:+	srna:+	tiling:+	rna_seq:+
AT2G14378	est:+	unigene:+	mpss:	srna:	tiling:+	rna_seq:+
AT2G14800	est:	unigene:+	mpss:+	srna:+	tiling:+	rna_seq:+
AT2G19850	est:	unigene:+	mpss:	srna:+	tiling:+	rna_seq:+
AT2G20130	est:+	unigene:	mpss:+	srna:	tiling:+	rna_seq:+
AT2G31300	est:+	unigene:	mpss:+	srna:	tiling:+	rna_seq:+

AT2G43440	est:	unigene:+	mpss:	srna:+	tiling:+	rna_seq:+
AT3G02240	est:	unigene:	mpss:+	srna:	tiling:+	rna_seq:+
AT3G02620	est:+	unigene:+	mpss:+	srna:	tiling:+	rna_seq:+
AT3G05160	est:+	unigene:+	mpss:+	srna:	tiling:+	rna_seq:+
AT3G10113	est:	unigene:+	mpss:	srna:	tiling:+	rna_seq:+
AT3G17712	est:+	unigene:+	mpss:	srna:	tiling:	rna_seq:+
AT3G23510	est:+	unigene:+	mpss:	srna:+	tiling:+	rna_seq:+
AT3G25960	est:	unigene:+	mpss:	srna:	tiling:+	rna_seq:
AT3G27503	est:	unigene:	mpss:	srna:	tiling:+	rna_seq:+
AT3G28300	est:	unigene:	mpss:	srna:	tiling:	rna_seq:+
AT3G28956	est:+	unigene:+	mpss:	srna:+	tiling:+	rna_seq:+
AT3G29255	est:	unigene:+	mpss:	srna:+	tiling:+	rna_seq:+
AT3G29260	est:	unigene:+	mpss:	srna:	tiling:+	rna_seq:+
AT3G45700	est:	unigene:+	mpss:+	srna:	tiling:+	rna_seq:+
AT3G47760	est:	unigene:	mpss:	srna:+	tiling:+	rna_seq:+
AT3G49420	est:	unigene:+	mpss:+	srna:+	tiling:+	rna_seq:+
AT4G00020	est:+	unigene:	mpss:+	srna:+	tiling:+	rna_seq:+
AT4G01180	est:	unigene:+	mpss:	srna:+	tiling:+	rna_seq:+
AT4G10860	est:	unigene:+	mpss:	srna:+	tiling:+	rna_seq:+
AT4G13500	est:+	unigene:+	mpss:	srna:+	tiling:+	rna_seq:+
AT4G14700	est:+	unigene:	mpss:+	srna:	tiling:+	rna_seq:+
AT4G15230	est:	unigene:	mpss:+	srna:	tiling:+	rna_seq:+
AT4G19760	est:	unigene:+	mpss:	srna:	tiling:+	rna_seq:
AT4G21460	est:+	unigene:+	mpss:+	srna:+	tiling:+	rna_seq:+
AT4G23420	est:+	unigene:+	mpss:+	srna:+	tiling:+	rna_seq:+
AT4G33320	est:	unigene:+	mpss:	srna:	tiling:+	rna_seq:+
AT4G34900	est:	unigene:	mpss:	srna:+	tiling:+	rna_seq:+
AT4G38320	est:+	unigene:	mpss:	srna:	tiling:+	rna_seq:+
AT5G06420	est:+	unigene:+	mpss:	srna:	tiling:+	rna_seq:+
AT5G25754	est:+	unigene:+	mpss:	srna:+	tiling:+	rna_seq:+
AT5G28900	est:+	unigene:+	mpss:+	srna:+	tiling:+	rna_seq:+
AT5G36670	est:	unigene:+	mpss:	srna:+	tiling:	rna_seq:+
AT5G36710	est:	unigene:+	mpss:	srna:	tiling:	rna_seq:+
AT5G36722	est:	unigene:+	mpss:	srna:	tiling:	rna_seq:
AT5G36738	est:	unigene:+	mpss:	srna:+	tiling:	rna_seq:+
AT5G36739	est:	unigene:+	mpss:	srna:+	tiling:	rna_seq:+
AT5G36780	est:	unigene:	mpss:	srna:+	tiling:	rna_seq:+
AT5G37270	est:	unigene:+	mpss:	srna:+	tiling:+	rna_seq:
AT5G39140	est:	unigene:+	mpss:	srna:+	tiling:	rna_seq:
AT5G39160	est:	unigene:+	mpss:	srna:+	tiling:	rna_seq:+
AT5G43620	est:	unigene:+	mpss:+	srna:+	tiling:+	rna_seq:+
AT5G50530	est:	unigene:+	mpss:	srna:+	tiling:	rna_seq:+
AT5G50600	est:	unigene:	mpss:	srna:	tiling:	rna_seq:+
ATMG00200	est:	unigene:	mpss:	srna:	tiling:	rna_seq:+
ATMG00440	est:	unigene:	mpss:	srna:+	tiling:	rna_seq:+
ATMG00550	est:	unigene:	mpss:	srna:+	tiling:	rna_seq:
ATMG00620	est:	unigene:	mpss:	srna:+	tiling:	rna_seq:+
ATMG01090	est:	unigene:	mpss:	srna:+	tiling:	rna_seq:+
ATMG01140	est:	unigene:	mpss:	srna:+	tiling:	rna_seq:
ATMG01150	est:	unigene:	mpss:	srna:+	tiling:	rna_seq:+
