# Supplementary material for: Divergent Evolutionary and Expression Patterns between Lineage Specific New Duplicate Genes and Their Parental Paralogs in Arabidopsis thaliana
Source: PLoS One. 2013 Aug 29;8(8):e72362. doi: 10.1371/journal.pone.0072362 (PMC3756979; doi:10.1371/journal.pone.0072362)
Supplement: Table S15 — 17 gene pairs with low methylation conservation. (PDF) [file pone.0072362.s020.pdf]

Table S15 17 gene pairs with low methylation conservation

gene_pair	conserved cytosines	unconserved cytosine	percentage of conserved cytosine	binominal test P value
AT1G14185:19655197_AT1G14190:19657215	188	12	0.94	0.00445189
AT1G29830:19651569_AT1G29820:19650884	191	14	0.931707317	0.000784577
AT1G30974:19654545_AT1G30972:19651850	32	4	0.888888889	0.01200185
AT1G33607:19650640_AT5G08055:19666983	26	4	0.866666667	0.006551755
AT1G61430:19649916_AT1G61440:19656647	336	17	0.95184136	0.006924308
AT1G70320:19654219_AT1G55860:19652907	1590	59	0.96422074	0.00388735
AT1G72590:19654648_AT2G16530:19641588	141	10	0.933774834	0.004619855
AT2G02840:19642349_AT2G06904:19642640	47	10	0.824561404	1.95E-06
AT2G07715:19643353_ATMG00560:19643641	8	7	0.533333333	4.52E-08
AT2G13450:19643397_AT4G02000:19649061	112	21	0.842105263	4.89E-11
AT2G31300:19638866_AT2G30910:19642421	94	7	0.930693069	0.01220962
AT3G17712:19665241_AT3G17740:19664924	79	10	0.887640449	9.79E-05
AT3G23510:19661058_AT3G23530:19660122	345	29	0.922459893	2.32E-07
AT4G15230:19648099_AT4G15215:19646795	630	25	0.961832061	0.01659014
AT5G37270:19672510_AT5G37230:19665305	60	14	0.810810811	6.96E-09
AT5G39160:19668693_AT5G39190:19672438	5	2	0.714285714	0.01270747
ATMG01090:19643677_AT2G07777:19640185	12	5	0.705882353	5.65E-05
