# Supplementary material for: Divergent Evolutionary and Expression Patterns between Lineage Specific New Duplicate Genes and Their Parental Paralogs in Arabidopsis thaliana
Source: PLoS One. 2013 Aug 29;8(8):e72362. doi: 10.1371/journal.pone.0072362 (PMC3756979; doi:10.1371/journal.pone.0072362)
Supplement: Table S17 — Three new genes that not only switch their tissue expression specificity but also show selection signature. (PDF) [file pone.0072362.s022.pdf]

Table S17. Three new genes that not only switch their tissue expression specificity but also show selection signature

gene name	function
AT4G14700	encodes origin of replication complex 1a subunit
AT1G29410	encodes phosphoribosylanthranilate isomerase
AT1G21530	involved in metabolic process
